# The *Physarum polycephalum* Genome Reveals Extensive Use of Prokaryotic Two-Component and Metazoan-Type Tyrosine Kinase Signaling

**DOI:** 10.1093/gbe/evv237

**Published:** 2015-11-27

**Authors:** Pauline Schaap, Israel Barrantes, Pat Minx, Narie Sasaki, Roger W. Anderson, Marianne Bénard, Kyle K. Biggar, Nicolas E. Buchler, Ralf Bundschuh, Xiao Chen, Catrina Fronick, Lucinda Fulton, Georg Golderer, Niels Jahn, Volker Knoop, Laura F. Landweber, Chrystelle Maric, Dennis Miller, Angelika A. Noegel, Rob Peace, Gérard Pierron, Taeko Sasaki, Mareike Schallenberg-Rüdinger, Michael Schleicher, Reema Singh, Thomas Spaller, Kenneth B. Storey, Takamasa Suzuki, Chad Tomlinson, John J. Tyson, Wesley C. Warren, Ernst R. Werner, Gabriele Werner-Felmayer, Richard K. Wilson, Thomas Winckler, Jonatha M. Gott, Gernot Glöckner, Wolfgang Marwan

**Affiliations:** ^1^School of Life Sciences, University of Dundee, Dundee, United Kingdom; ^2^Magdeburg Centre for Systems Biology and Institute for Biology, University of Magdeburg, Magdeburg, Germany; ^3^The Genome Institute, Washington University School of Medicine, St Louis; ^4^Department of Biological Sciences, Graduate School of Science, Nagoya University, Furocho, Chikusaku, Nagoya, Aichi, Japan; ^5^Department of Molecular Biology and Biotechnology, University of Sheffield, Firth Court, Western Bank, Sheffield, United Kingdom; ^6^UPMC Univ Paris 06, Institut de Biologie Paris-Seine (IBPS), CNRS UMR-7622, Paris, France; ^7^Biochemistry Department, Schulich School of Medicine and Dentistry, Western University, London, Ontario, Canada; ^8^Department of Biology and Center for Genomic and Computational Biology, Duke University, Durham; ^9^Department of Physics, Duke University, Durham; ^10^Department of Physics and Center for RNA Biology, The Ohio State University, Columbus; ^11^Department of Chemistry & Biochemistry, The Ohio State University, Columbus; ^12^Division of Hematology, Department of Internal Medicine, The Ohio State University, Columbus; ^13^Department of Ecology & Evolutionary Biology, Princeton University, Princeton; ^14^Biological Chemistry, Biocenter, Innsbruck Medical University, Innsbruck, Austria; ^15^Genome Analysis, Leibniz Institute on Aging - Fritz Lipmann Institute (FLI), Jena, Germany; ^16^IZMB - Institut für Zelluläre und Molekulare Botanik, Universität Bonn, Bonn, Germany; ^17^Institut Jacques Monod, CNRS UMR7592, Université Paris Diderot Paris7, Paris, France; ^18^The University of Texas at Dallas, Biological Sciences, Richardson; ^19^Institute for Biochemistry I, Medical Faculty, University of Cologne, Cologne, Germany; ^20^Carleton University, Ottawa, Ontario, Canada; ^21^Institute for Anatomy III / Cell Biology, BioMedCenter, Ludwig-Maximilians-Universität, Planegg-Martinsried, Germany; ^22^Institut für Pharmazie, Friedrich-Schiller-Universität Jena, Jena, Germany; ^23^Department of Biological Sciences, Graduate School of Science and JST ERATO Higashiyama Live-holonics Project, Nagoya University, Furocho, Chikusaku, Nagoya, Aichi, Japan; ^24^Department of Biological Sciences, Virginia Polytechnic Institute and State University, Blacksburg; ^25^Center for RNA Molecular Biology, Case Western Reserve University, School of Medicine, Cleveland; ^26^Leibniz Institute of Freshwater Ecology and Inland Fisheries (IGB), Berlin, Germany

**Keywords:** Amoebozoa, tyrosine kinase receptor, two-component system, signaling, phytochrome

## Abstract

*Physarum polycephalum* is a well-studied microbial eukaryote with unique experimental attributes relative to other experimental model organisms. It has a sophisticated life cycle with several distinct stages including amoebal, flagellated, and plasmodial cells. It is unusual in switching between open and closed mitosis according to specific life-cycle stages. Here we present the analysis of the genome of this enigmatic and important model organism and compare it with closely related species. The genome is littered with simple and complex repeats and the coding regions are frequently interrupted by introns with a mean size of 100 bases. Complemented with extensive transcriptome data, we define approximately 31,000 gene loci, providing unexpected insights into early eukaryote evolution. We describe extensive use of histidine kinase-based two-component systems and tyrosine kinase signaling, the presence of bacterial and plant type photoreceptors (phytochromes, cryptochrome, and phototropin) and of plant-type pentatricopeptide repeat proteins, as well as metabolic pathways, and a cell cycle control system typically found in more complex eukaryotes. Our analysis characterizes *P. polycephalum* as a prototypical eukaryote with features attributed to the last common ancestor of Amorphea, that is, the Amoebozoa and Opisthokonts. Specifically, the presence of tyrosine kinases in *Acanthamoeba* and *Physarum* as representatives of two distantly related subdivisions of Amoebozoa argues against the later emergence of tyrosine kinase signaling in the opisthokont lineage and also against the acquisition by horizontal gene transfer.

## Introduction

**Fig. 1.—:**
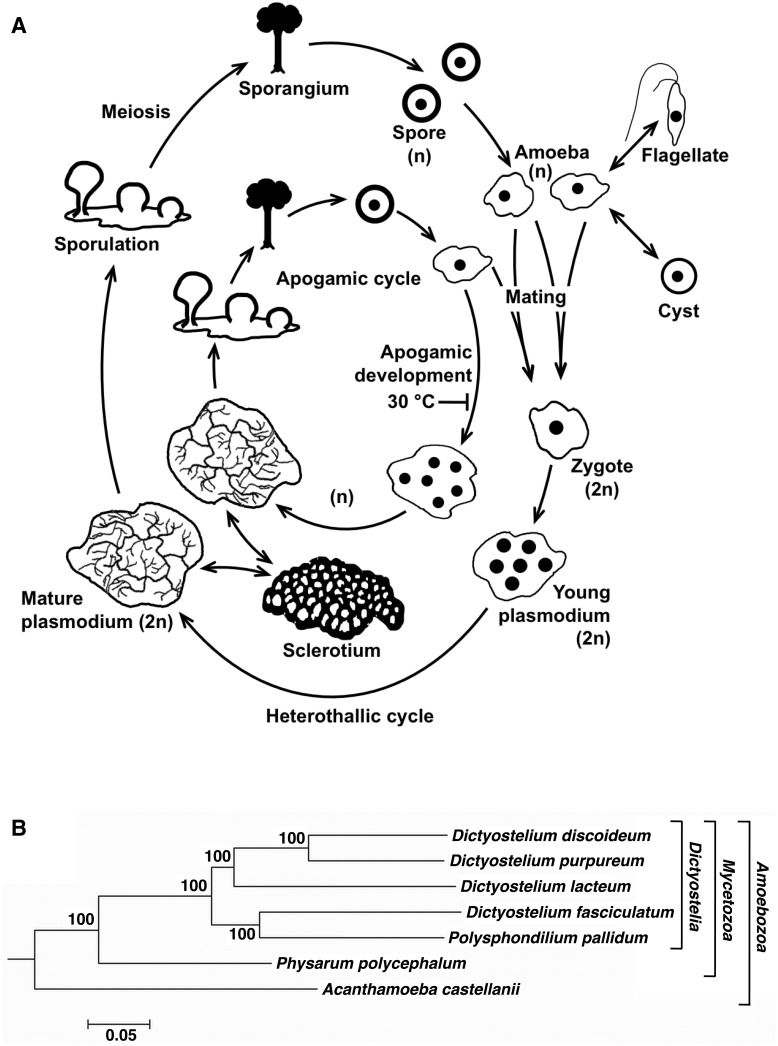
*P. polycephalum*, life cycle and relationship to other Amoebozoa. (*A*) Schematic representation of the different stages that form during the heterothallic or the apogamic life cycle, as represented by the outer or the inner circle, respectively. One haploid (n) mononucleate amoeba hatches from each germinating haploid mononucleate spore. Amoebae have four developmental options. They can propagate by cell division through an open mitosis or differentiate into a flagellate, a cyst or a multinucleate plasmodium. **Heterothallic cycle:** Two amoebae of different mating type mate with each other to form a mononucleate diploid (2n) zygote by karyogamy. The zygote develops into a diploid multinucleate plasmodium. In the plasmodium, nuclei divide synchronously, while the nuclear envelopes remain intact (closed mitosis). The plasmodium keeps growing as long as environmental conditions are favorable. A mature, multinucleate plasmodium can develop into a sclerotium to survive drought. Sporulation of a starving plasmodium is induced by visible light or heat shock. During sporulation, the protoplasmic mass develops into multiple fruiting bodies. Each sporangium contains hundreds of haploid (n), mononucleate spores that have been formed through meiosis. **Apogamic cycle:** A haploid, mononucleate amoeba that carries a *gadAh* mutant allele may develop into a multinucleate, haploid plasmodium without mating. Upon sporulation, the low number of diploid nuclei that have been formed within the plasmodium gives rise to viable spores. Apogamic development can be suppressed experimentally by elevated temperature, which allows propagation of amoebal clones or the formation of a diploid plasmodium by mating of two amoebae of different mating type. As mating of amoebal cells and plasmodium formation are controlled by different mating type genes, sophisticated approaches based on Mendelian genetics are possible (Dee 1987). (*B*) Phylogenetic subtree of some Amoebozoa species with completed genomes. The tree is based on 30 highly conserved genes and rooted with other eukaryotes. The complete tree including representatives from plants, animals, and fungi is shown in supplementary fig S22. The maximum likelihood method with the JTT matrix was used. *Entamoeba histolytica* genes were omitted for calculating the tree, because endoparasites are known to evolve at different rates as compared with free-living organisms. For details, see Supplemental Methods, Results, and Discussion.

*Physarum polycephalum* belongs to the Amoebozoa, the sister group to the Opisthokonts (i.e., fungi and animals) (Cavalier-Smith 2003) which both together form the supergroup Amorphea (Adl et al. 2012). In the course of its complex life cycle (fig. 1*A*), *P. polycephalum* is able to differentiate into various specialized cell types depending on environmental conditions (Burland et al. 1993). Macroscopic multinucleate plasmodial cells contain a naturally synchronous replicating and differentiating population of nuclei and can grow to tens or even hundreds of centimeters in size. Individual pieces cut off from a single plasmodium maintain synchrony, providing unique experimental options for single-cell biology. The occurrence of open and closed mitosis in amoebae and plasmodia, respectively, is another well-known aspect of its rich cell biology (Aldrich and Daniel 1982; Sauer 1982; Dove et al. 1986). Accordingly, *P. polycephalum* has served as a classical model organism in cell and developmental biology since the early 1960s and has been used extensively to study cell cycle regulation, cell differentiation (amoeba-flagellate transition, spherulation, sporulation), cell fusion, DNA replication, developmental gene expression, histone modification, sensing, and response (e.g., chemotaxis) (for reviews see Aldrich and Daniel 1982; Sauer 1982; Dove et al. 1986; Burland et al. 1993). More recently, *P. polycephalum* plasmodia have been used for studies ranging from cell biology and biophysics to unconventional computing for path finding, the biosensory control of robots, or the generation of music (Tsuda et al. 2007; Tero et al. 2010; Braund and Miranda 2014). Plasmodial cells also contain thousands of mitochondria, providing the means to study the highly unusual form of insertional RNA editing used to generate functional mitochondrial transcripts.

From an evolutionary biology point of view, *P. polycephalum* is interesting because of its placement into the Amoebozoa, where the genomes of several distantly related representatives (*Acanthamoeba castellanii*, *Entamoeba histolytica*, *etc.*) are already known (fig. 1*B*). The model organism most closely related to *P. polycephalum* for which a genome sequence has previously been determined is *Dictyostelium discoideum* (Eichinger et al. 2005). The two model species differ in several important aspects. *D. discoideum* is a cellular slime mold where differentiation occurs in developmentally interacting cells, whereas in the acellular slime mold *P. polycephalum,* a single, multinucleate plasmodial cell differentiates in a manner reminiscent of *Drosophila* embryos or other developing syncytia. A comparison of *P. polycephalum* and *D. discoideum* may help to elucidate the different evolutionary trajectories the members of the two branches, cellular and acellular slime molds, have taken in establishing sophisticated life cycles that are so different.

The established lab strains of *D. discoideum* do not form viable progeny after mating, which hinders genetic experiments, whereas mating of strains and segregation of meiotic progeny in *P. polycephalum* is under ready experimental control. The classical genetics are well-established in *P. polycephalum,* and the possibility to experimentally switch the life cycle between haploid and diploid stages (fig. 1*A*) is very useful for the generation and analysis of mutants (Dee 1982, 1987). Amoebal strains derived from different natural plasmodial isolates, such as Wis-1 and Wis-2 (see Appendix A of Supplemental Methods, Results, and Discussion), allow forward genetic approaches by mapping genes identified in phenotypic screens. Mendelian analysis based on the co-segregation of phenotype and single nucleotide polymorphisms (Schedl and Dove 1982) can be performed by sequencing pools of cDNAs of phenotype-carrying segregants (Barrantes and Marwan, unpublished results). These high-throughput methods combined with Mendelian genetics mitigate current difficulties of reproducibly making transgenic lines. Methods for knocking down gene expression have recently been developed for *P. polycephalum* (Haindl and Holler 2005; Itoh et al. 2011), opening up additional experimental avenues. Here, we present an overview of the whole genome of *P. polycephalum*, focusing on some of its peculiar features such as its surprisingly extended signaling system.

## Materials and Methods

### Assembly

Combined sequence reads from different libraries (including 3- and 8-kb mate pairs) were assembled using the Newbler software version 2.6 (Margulies et al. 2005), and the Newbler contigs were then assembled into scaffolds using Bambus version 2.3 (Pop et al. 2004), and a custom transcriptome-guided scaffolding algorithm. The 10.0 draft assembly is composed of 69,687 scaffolds with an N50 scaffold length of 54,474 kb and an N50 contig length of 2.2 kb. The assembled coverage is 54.6×, and the assembly spans over 220 Mb. We removed from the assembly all contaminating sequences, trimmed vectors (X), and ambiguous bases (N). Additionally, shorter contigs (≤200 bp) were removed before public release.

The draft genome sequence of *P. polycephalum* was deposited in the DDBJ/EMBL/GenBank database (Accession Number ATCM00000000.3). The genome data can also be downloaded from http://www.physarum-blast.ovgu.de/.

### Generation of a Reference Transcriptome

All transcript data were assembled according to their sample origin, as previous analyses showed that genes have a wealth of introns and thus alternative splicing could play a role (Glöckner et al. 2008). This initial analysis yielded 733,443 potential transcripts.

After clustering with usearch (Edgar 2010), we obtained 31,770 clusters, each represented by the largest sequence in a cluster. The mean length of transcripts in the thus constructed reference transcriptome is 1,264 bases. These transcripts were mapped onto the genome using blat (Kent 2002). Only 485 transcripts could not be mapped to the genome sequence, of which only 90 have a blast score of 250 or higher in a search against the reference sequence database (NCBI July 2013). Fifteen of these 90 appear to be of *P. polycephalum* mitochondrial origin and a further 13 are certainly contaminants from the sequencing process, as they have identity values higher than 85% to some bacteria and mouse sequences. Thus, only a minor fraction of transcripts is not represented by the assembled genome. Manual inspection of some transcript/genome alignments revealed that transcripts frequently span gaps in the scaffolds, which account for missing small exons. Inability to assemble genome junctions at these positions is likely caused by the low complexity of intron sequences and the small size of the missing exons. As contiguity of a sequence is required for accurate gene prediction, we relied heavily on the transcript data for annotation and analysis. The Transcriptome Reference Assembly has been deposited at DDBJ/EMBL/GenBank under the accession GDRG00000000. The version described in this paper is the first version, GDRG01000000. All transcriptome sequence data and files containing the gene loci-transcript conversion, the transcript map, and the automatic annotation of the reference transcriptome can be downloaded from http://www.physarum-blast.ovgu.de/.

### Gene Prediction and Construction of Gene Loci

For an *ab initio* gene prediction, we used Augustus (Stanke et al. 2004). The transcriptome data were used to train this program according to the manual, and to provide splice information for the algorithm. The program itself was run with default values. This way we predicted 47,779 protein coding genes. We noticed, however, that the fragmented nature of the genome sequence and missing contiguity within scaffolds led to an overprediction. To get a better estimate of the number of protein coding genes in the *P. polycephalum* genome, we tried to combine gene prediction and reference transcriptome for the definition of gene loci. To this end, we mapped the reference transcriptome data to the genome. We then fused predicted genes and reference transcripts to gene loci if predicted genes were covered by the same reference transcript and/or if predicted genes were present in the near vicinity (<50 bases apart) and at least one of them was covered or partially covered by a transcript sequence. Predicted genes without transcript coverage and no transcript in the near vicinity (<50 bp apart) were counted as independent gene loci. In this way, we defined 34,438 gene loci, of which 17,280 are not supported by transcript data (table 1).

**Table 1:** Assembly, transcriptome, and gene prediction

Scaffold assembly size (Mb)	188.75
Number of scaffolds	55,119
Mean scaffold length	3,424
N50 scaffold length	65,980
Contig size (Mb)	173.6
Number of contigs	138,064
Mean contig length	1,256
N50 contig length	2,197
Transcriptome contigs	733,443
Reference transcriptome after clustering	31,770
Predicted gene models	47,779
Predicted genes without transcript support longer than 40 aa (all)	17,199 (17,280)
All gene loci (overlapping reference transcripts defined as one gene locus)	34,438

### Small RNAs

For the definition of small RNAs, we used RNAseq transcript data from a previous experiment (Bundschuh et al. 2011). As this specific experiment was performed on mitochondrial preparations, we only expect the more abundant nuclear small RNAs to be observed. However, the absence of poly-A selection and the use of a small RNA kit in library preparation promised a view orthogonal to traditional RNA-Seq approaches.

For detailed information on strains used, preparation of nucleic acids, and for bioinformatic methods, see supplementary file 1.

## Results and Discussion

### Genome Properties and Reference Transcriptome

The *P. polycephalum* genome proved to be highly recalcitrant for assembly owing to abundant and frequently long (mononucleotide or pyrimidine-rich) repeat stretches present in intergenic regions or within its numerous and highly variable intron sequences. Construction of gene models therefore heavily relied on accompanying transcriptome data (see Materials and Methods).

The genetic material used for sequencing the *P. polycephalum* genome was derived from axenic, haploid amoebal cells (strain LU352). The assembly of 454 and paired end Illumina reads with an estimated coverage of 50 × (calculating with a genome size of 250 Mb) resulted in scaffolds covering 182 Mb. However, assembling the genome has been challenging owing to extremely long stretches of di-, tri-, and tetranucleotide repeats and large homopolymeric tracts, which likely lead to polymerase slippage and premature polymerase termination *in vitro.* The unresolvable sequence patches cause innumerable gaps in the final sequence, resulting in a mean scaffold size of only slightly larger than 2 kb. PacBio sequencing yielded only minimal improvement. We therefore tested whether our assembly captures most of the coding information of the genome by investigating the primary metabolism capacity encoded by the assembly. We found that all expected pathways were entirely present and, therefore, conclude that the current assembly is complete enough for further analysis.

#### Gene Content and Introns

To further evaluate gene content and completeness, we generated transcriptome data sets from multiple life-cycle stages and constructed a reference transcriptome (see Materials and Methods).

We next trained the gene prediction program Augustus (Stanke et al. 2004) with sequences derived from mapped transcripts. Gene predictions using the splicing information from all transcripts initially yielded more than 47,000 gene models (table 1). However, because in the current assembly some shorter introns and exons are missing in scaffolds owing to simple repeat sequences at both ends, we used the reference transcripts together with the gene predictions to define gene loci. Transcripts and predicted genes overlapping each other or being in close vicinity on the assembly (in the range of an intron length) were fused to a common gene locus. A total of 34,438 gene loci were defined in this way (table 1), half of which are represented by transcripts (17,158). The gene loci with corresponding transcripts have a mean length of 1951 bases, whereas those without transcript support have a mean length of only 579 bases. This suggests that the majority of unsupported gene loci may represent false-positive predictions, which is fairly typical of highly fragmented genome assemblies.

There are scores of complex repeats in the genome, which contains a minimum of 484 integrase domains (PF00665) and 1,014 reverse transcriptase domains (PF07727 and PF00078). This greatly exceeds the number present in other Amoebozoa species such as *A. castellanii* and *D. dis*c*oideum*, which contain only 38 and 118 reverse transcriptase domains, respectively. In total, *P. polycephalum* complex repetitive elements contribute approximately 3 Mb or 1.3% to the size of the genome (supplementary table S4). Most *P. polycephalum* genes contain multiple introns, many of which are composed of extended repeats flanked by splicing signals. The median size of the 160,988 introns confirmed by transcript data is 231 bases, and the total number of predicted introns is 676,387. Thus, the mean number of confirmed introns per gene is approximately 5. If only the genes with transcript data are counted, the number of introns per gene increases to well above 9. This intron frequency is higher than the estimated number of introns in the last common ancestor (LCA) of eukaryotes (Zhou et al. 2014). Thus, *P. polycephalum* has likely acquired introns during its species history.

#### Domain Analysis

Using iprscan (Zdobnov and Apweiler 2001), we defined the domain content for each gene locus. A comparison with *A. castellanii* and *D. discoideum* showed that *P. polycephalum* has the highest number of domain hits of the three organisms, reflecting the larger genome and the higher gene count. In terms of distinct domains, *A. castellanii* and *P. polycephalum* are similar, whereas fewer types of domains have been identified in *D. discoideum,* suggesting domain loss in the social amoebae (table 2). Further analysis of domains revealed that 304 domains were present at least once in the genomes of *P. polycephalum* and *A. castellanii*, but absent from *D. discoideum* (supplementary table S2). Among these are a considerable number of domains associated with signaling functions. Domains enriched compared with those of *A. castellanii* and *D. discoideum* include transposon domains and, again, domains associated with signaling functions (supplementary table S3).

**Table 2:** Domain distribution in three Amoebozoa

	*P. polycephalum*	*D. discoideum*	*A. castellanii*
All domains	19,611	11,307	17,560
Different domains	3,504	3,192	3,542

### Non-Coding RNAs

Small non-coding RNAs perform important functions in all organisms, but can be difficult to annotate *de novo* owing to their short length and their poorly defined sequence patterns. However, data obtained in a previous mitochondrial RNA-Seq experiment allowed us to annotate a number of small non-coding *P. polycephalum* RNAs encoded in the nuclear genome. This resulted in the confirmation of many known small *P. polycephalum* RNAs (Supplementary Spreadsheet 1, first sheet). In addition, we found 24 novel transfer RNAs, the spliceosomal U2 and U6 RNAs, 30 box C/D snoRNAs, 9 novel lncRNAs, which clustered into three groups by sequence similarity, as well as 24 RNAs shorter than 200 nucleotides clustering into three groups and 28 singleton RNAs shorter than 200 nucleotides (Supplementary Spreadsheet 1, second sheet). These latter RNAs and the box C/D snoRNAs have no detectable sequence similarity with any sequence in the non-redundant nucleotide database.

We ran tRNAscan-SE (PMID: 9023104) with default parameters on the genome, which resulted in 319 putative tRNA loci and 248 unique putative tRNA sequences (Supplementary Spreadsheet 1, third sheet). These included all but one (tRNA-Tyr1 in Supplementary Spreadsheet 1, second sheet) of the 56 new tRNAs identified from the sequencing experiment as well as all previously annotated *P. polycephalum* tRNAs with the exception of tRNA-Lys1, tRNA-Ser2, and tRNA-Asp1. A total of 201 putative tRNA loci with 156 unique putative tRNA sequences were novel. These included tRNAs for all 20 amino acids as well as 6 putative tRNA-SeC loci (each with a unique sequence) and four putative tRNA loci of undetermined type (each with a unique sequence).

### PPR Proteins

The *P. polycephalum* genome is particularly rich in genes encoding pentatricopeptide repeat (PPR) proteins. PPR proteins are sequence-specific RNA-binding proteins that act in diverse processes of RNA maturation, mainly in mitochondria and chloroplasts (Schmitz-Linneweber and Small 2008). The PPR motif is a loosely conserved 35-aa motif identifying individual ribonucleotides on a one-repeat-per-nucleotide basis (Barkan et al. 2012). As in other taxa, the *P. polycephalum* proteins contain PPR arrays of variable sizes ranging from only a few up to more than 20 tandem PPRs in individual proteins. With some 100 PPR protein genes, *P. polycephalum* features much more than tenfold the number found in other slime mold genomes such as *Polysphondylium pallidum* or *D. discoideum.* In fact, the set of PPR proteins present in *P. polycephalum* is significantly larger than in other Amoebozoa and, more broadly, within the Amorphea super-domain at large. Indeed, larger numbers of PPR proteins have hitherto only been observed in Dinoflagellates and in land plants, where large families of PPR proteins were initially discovered (Small and Peeters 2000). Two observations are particularly intriguing: In contrast to other Amorphea, *P. polycephalum* has a highly derived mitochondrial genome, the transcripts of which are affected by abundant and diverse types of RNA editing, including alterations of transcript sequences by nucleotide insertions and base conversions (Mahendran et al. 1991; Takano et al. 2001; Bundschuh et al. 2011). We speculate that some of the PPR proteins in *P. polycephalum* take part in these processes. Most notably, 16 of the *P. polycephalum* PPR proteins are “plant-like” in carrying a carboxyterminal DYW domain with cytidine deaminase similarity (supplementary fig. S21, Supplementary Material online) (Schallenberg-Rüdinger et al. 2013). Several such DYW-type PPR proteins have been characterized as site-specific C-to-U editing factors in plants. Intriguingly, the first discovery of PPR proteins with a DYW domain outside of land plants in the genome of *Naegleria gruberi* led to the subsequent discovery of C-to-U editing in the mitochondria of this heterolobosean protist (Knoop and Rüdinger 2010; Rüdinger et al. 2011). As in plants and *Naegleria*, the residues likely coordinating a zinc ion for deaminase activity are highly conserved in the *P. polycephalum* DYW domains. Given that in its natural environment there is direct physical contact with the slime mold growing on decaying plant materials, horizontal gene transfer (HGT) from plants may be a possible source of the *P. polycephalum* PPR genes. However, no evidence for particularly high similarity of any *Physarum* PPR protein to a homolog in another taxon indicating a recent HGT has been found. The DYW-type PPR protein families of *Physarum polycephalum, Naegleria gruberi,* and the moss *Physcomitrella patens,* for example, cluster taxon-wise without evidence for recent HGT (supplementary fig. S21C, Supplementary Material online). Moreover, whereas plant DYW-type PPR proteins feature characteristic “PLS-type” PPR arrays with alternating long (L) and short (S) variants of the classic (P) PPRs, most *Physarum* DYW-type homologs display “LS-type” repeats (supplementary fig. S21A, Supplementary Material online).

### Metabolism and the Cytoskeleton

#### Pteridine Metabolism

Pteridines comprise a group of molecules that contain pteridine (pyrimido [4,5-b] pyrazine), a bicyclic ring system, as a common structural element. Pteridines (e.g., folate, biopterin, riboflavin, etc.) function as important cofactors (or their precursors) of various enzymatic reactions. Pterines are synthesized from the common precursor GTP (guanosine 5′ triphosphate).

Analysis of the *P. polycephalum* genome for pteridine metabolic enzymes revealed that it is unusual in encoding enzymes for all common pteridine biosynthetic pathways (for the biosynthesis of molybdopterin, tetrahydrofolate, tetrahydrobiopterin, 7-aminomethyl deazaguanine, and riboflavin) and, in addition, the enzymes alkylglycerol monooxygenase and nitric oxide synthases. Alkylglycerol monooxygenase and nitric oxide synthase, in its full length form with oxygenase and reductase domains, are regularly found only in animals. Nitric oxide synthases are required for sporulation in *P. polycephalum* by acting via cGMP signaling (Golderer et al. 2001). This link between animal-type NO synthases and cyclic nucleotide signaling, which is widely used in *P. polycephalum* (see Section Signaling), is noteworthy and to date is unique in unicellular eukaryotes. This suggests an ancient origin of this typically animal-type signaling enzyme and its signal transduction mechanism via cGMP.

Another unique finding is that riboflavin biosynthetic enzymes are encoded as two tri-functional reading frames (supplementary fig. S10, Supplementary Material online) rather than by separate cistrons, a feature thus far not observed for sequences from any other species represented in GenBank.

*P. polycephalum* is much more diverse than *D. discoideum* in its pteridine metabolic equipment. It contains enzymes for the biosynthesis of 7-aminomethyl-7-deaza guanine (preQ1), a precursor for the special tRNA base queuosine (Q), as well as for the biosynthesis of riboflavin. The reading frames for nitric oxide synthases found in *P. polycephalum* do not occur in the *D. discoideum* genome. Furthermore, another surprising feature is the comparatively frequent presence of homologous enzymes that catalyze the same enzymatic reaction, whereas most species including *D. discoideum* use only one. These homologs share in the mean 72% sequence similarity, have the motifs required for enzymatic activity conserved, are expressed, and are therefore expected to be enzymatically active. This assumption has been experimentally confirmed for the two inducible nitric oxide synthase homologs NOS2a and NOS2b (Messner et al. 2009). The reasons for or advantages of this apparent redundancy are currently unknown. On the other hand, *D. discoideum* produces an additional unique pteridine, dictyopterin, an isomer of biopterin (Klein et al. 1990) that is missing in *P. polycephalum.*

For the 24 genes analyzed in supplementary table S5 encoding for 26 enzymes, closest orthologs for 12 were found in *Amoebozoa* (eight of these in *Dictyosteliida*), six in bacteria, four in animals, three in *Capsaspora owczarzaki*, an *Opisthokonta* of uncertain placing, and one in red algae (supplementary table S5).

#### Shikimate Pathway

The *P. polycephalum* genome also contains genes encoding enzymes for complete shikimate and aromatic amino acid synthesis pathways, which are not present in either the social amoebae or Entamoebae (Richards et al. 2006). In fungi, alveolates, and oomycetes, a pentafunctional polypeptide is formed (AROM). This organization is also observed in *P. polycephalum* (gene locus 13138), strongly suggesting that this is the ancestral state of the shikimate pathway protein domain structure. In contrast, it was recently shown that *A. castellanii* has an unusual arrangement of such pathway genes (Henriquez et al. 2015), which might represent a later rearrangement within the Amoebozoa clade. Like all other free-living Amoebozoa so far, *P. polycephalum* contains a phosphoenolpyruvate carboxylase (gene_locus_38419), which is primarily found in bacteria and plants. The purpose of this enzyme could be to supply the tricarboxylic acid cycle with C4 bodies when they are required for amino acid biosynthesis.

#### Actin Cytoskeleton and Motor Domains

The very intriguing oscillatory cytoplasmic streaming in *P. polycephalum* triggered extensive studies on the cytoskeleton. It was first reported by Noburo Kamiya (Kamiya 1940), that is, long before actin and myosin had been identified as driving forces in muscle and non-muscle cells. At up to 1,350 µm/sec, the rhythmic streaming in different developmental stages of *P. polycephalum* is among the fastest intracellular motilities known to date (Kamiya 1959; Wohlfarth-Bottermann 1979). For comparison, an average kinesin moves with a velocity of about 0.6 µm/sec along a microtubular track.

In the current study, many major families of actin-binding proteins have been found (Supplementary Spreadsheet 2). The profilins as members of G-actin sequestering proteins, fragmin as representative of the Ca^2+^-dependent F-actin severing proteins, the heterodimeric F-actin capping proteins (formerly “*Physarum* beta-actinin”), F-actin crosslinking proteins like alpha-actinin and filamin, myosins as major motor proteins, and formin-like proteins with FH1 and FH2 domains are present in comparable numbers as in *D. discoideum* and more complex organisms. Even peculiar actin isoforms like *D. discoideum* filactin, an actin with a large N-terminal extension (Joseph et al. 2008), could be identified in the *P. polycephalum* genome.

A seemingly unique and exciting feature of the actin cytokskeleton in *P. polycephalum*, however, is the presence of the actin-fragmin kinase (Contig 47652). Fragmin, homologous to severin in *D. discoideum* and gelsolin in more complex organisms (Yin et al. 1990), was first described by the Hatano group (Hasegawa et al. 1980) and its 1:1 complex with actin was further investigated by Vandekerckhove and colleagues (Gettemans et al. 1992; Eichinger et al. 1996). The latter group isolated a kinase that phosphorylated actin, but only in the 1:1 actin-fragmin complex. Upon phosphorylation, the activity of the actin-fragmin complex changed to an F-actin capping function and was, therefore, a putative regulator of fast changes in local viscoelasticities in the F-actin network, a prerequisite for cytoplasmic streaming. Surprisingly, the protein sequence of the purified protein did not show any of the well-known protein kinase domains (Hanks and Hunter 1995), and it could not be excluded that a minor contamination was responsible for actin phosphorylation. Only the crystallization of the protein finally showed that it was in fact a kinase whose structure was in its catalytic domain nearly identical to protein kinase A (Steinbacher et al. 1999). These data lead to major conclusions: The actin-fragmin kinase is clearly an example for convergent evolution. There is no common ancestor as in the case of divergent evolution of conventional protein kinases. Thus, the evolutionary pressure is on the structure of the molecule in determining its function. There is no need to keep a distinct amino acid sequence with a certain arrangement of protein domains, just structure/function relationships count. Consequently, this type of actin phosphorylation in *P. polycephalum* is possibly not unique as suggested above. One cannot exclude convergent evolution of kinases with similar substrate specificity also in other organisms, as these molecules may have completely unrelated sequences and are not easily detectable by sequence similarities. Perhaps most importantly, this example demonstrates the limitations of any whole-genome approach. In the end, only biochemistry and molecular cell biology will cross the border from “gene products of unknown function” to proteins with known activities.

*P. polycephalum* possesses a wealth of motor domains. We searched all defined gene loci for the presence of such domains. With this initial search, we found 43 gene loci with a kinesin domain (PF00225), 23 gene loci with a dynein heavy-chain domain (PF03028), and 53 gene loci with myosin motors (PF00063). As the genome assembly is fragmented and thus gene loci likely do not always represent a whole gene, we further investigated these loci making use of the reference transcriptome library. This approach enabled us to define eight complete dyneins, one N-terminal half, and two N-terminal fragments. Ten further fragments of varying length show homology to the middle and C-terminal parts of dyneins. Thus, the *P. polycephalum* genome likely encodes at least 11 dynein proteins. This number is in the same range as in other species.

With the same approach, we defined 15 complete or nearly complete myosin proteins and 16 fragments of varying lengths. Five of the full-length genes and seven of the fragments have the highest similarity to class VII unconventional myosins, indicating an amplification of this family in *P. polycephalum.*

Kinesins have a wider length range than the other motor proteins, and the regions outside the motor domain are highly variable. This makes it impossible to distinguish between full-length genes and fragments, as with the other motor domain proteins. Matching possible kinesin gene loci with transcript data yielded 39 defined loci. Three of these are small without transcript data. The mean length of the others is well above 700 amino acids. Thus, we conclude that at least 36 kinesin genes reside in the *P. polycephalum* genome. Strikingly, most of the gene loci we defined here are represented in our reference transcriptome library indicating their expression.

Dynein domains and, much more pronounced, kinesin domains are overrepresented in *P. polycephalum* as compared with two other organisms within the Amoebozoa, *D. discoideum* and *A. castellanii* (supplementary table S3). The enrichment of dynein domains is likely owing to the fact that *P. polycephalum* possesses flagella. It was shown previously that a plasmodial species, *Reticulomyxa filosa*, has amplified its complement of kinesin domain-bearing genes, which may be associated with the increased requirements of intracellular transport in a huge cell (Glöckner et al. 2014). We see this gene family expansion also in *P. polycephalum*, hinting at a common prerequisite for acquiring a plasmodial life stage.

#### Cell Cycle Regulation, Cellular Signaling, and Photoreceptors

The detection of external stimuli and the processing of these stimuli into an appropriate response is an essential property of all living organisms. For a free-living, motile, phagocytotic, unicellular organism like *P. polycephalum*, physiologically relevant stimuli may include environmental factors such as light, humidity, temperature, pH, osmolarity, mechanical stress, or chemical signals released by mates, prey, predators, competitors, or symbionts*.* As a true free-living species, *P. polycephalum* should have retained most, if not all, components of such signaling systems inherited from the LCA of Amoebozoa. As Opisthokonta and Amoebozoa form together the Amorphea (Adl et al. 2012), it might even be possible to trace inventions thought to have appeared only in Metazoa back to this LCA.

In eukaryotes including mammalian cells, much of the sensory input directly or indirectly feeds into the control of the mitotic or meiotic cell cycle as part of the sensory control of developmental decisions or programs. Therefore, genes encoding potential cell cycle regulatory proteins were also characterized.

#### Key Cell Cycle Regulators Are Conserved Between Amoebozoa and Metazoa

**Fig. 2.—:**
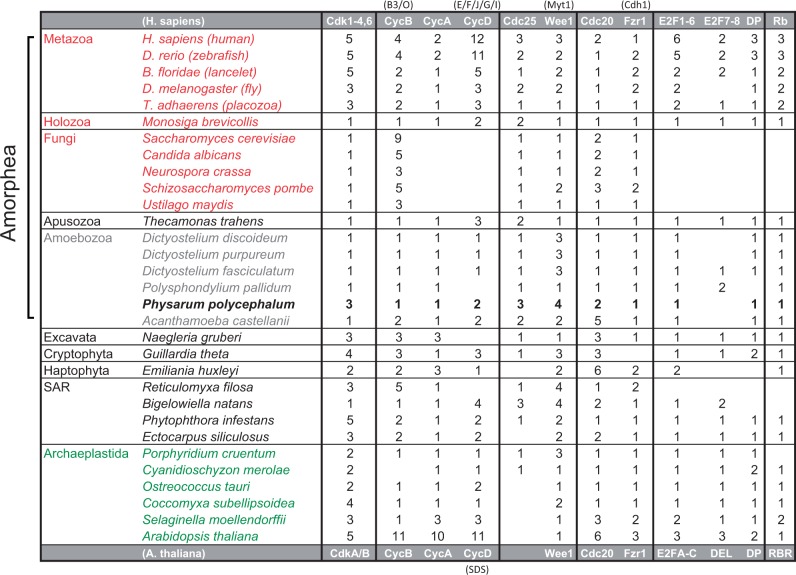
Analysis of key cell-cycle regulators across diverse eukaryotes. We searched each genome for cell division kinases (Cdk), cyclins (Cyc), G2/M regulators (Cdc25, Wee1), APC regulators (Cdc20, Fzr1), G1/S transcription factors (E2F/DP), and G1/S inhibitors (Rb); see Methods for details. Each entry is a conservative estimate of the number of family members in each genome. Top and bottom gray rows are common cell-cycle gene names from *Homo sapiens* and *Arabidopsis thaliana*, respectively. Sub-families or alternative names of cell-cycle genes are listed in parentheses. Metazoa (e.g., animals) and Fungi (e.g., yeasts) are members of the Opisthokonta (red), whereas Viridiplantae (e.g., land plants) are Archaeaplastida (green). *P. polycephalum* is a member of the Amoebozoa (gray), which are a sister group to the Opisthokonta. The last common ancestor of Amoebozoa and Opisthokonta is known as Amorphea (Adl et al. 2012).

Building on early, pioneering studies of eukaryotic cell-cycle regulation in *P. polycephalum* (Rusch et al. 1966; Sachsenmaier et al. 1972; Solnica-Krezel et al. 1991), the availability of its genome sequence now provides unique opportunities to study the functional dynamics of the regulation of mitosis, meiosis, and the coregulation of cell cycle and cell fate decision. By analyzing macroscopic samples taken at regular time intervals from a single plasmodial cell at subsequent points of a time series, one can exploit the remarkable natural mitotic synchrony of plasmodial nuclei (see Supplementary Results and Discussion) and resolve time-dependent regulatory events within individual cells at transcriptome and proteome-wide scales. In this context, the current finding that the control system of the mitotic cycle of *P. polycephalum* is typical of most eukaryotes, including plants and animals while different to that of the yeasts (fig. 2), makes an important point.

Much of our knowledge of the molecular mechanisms controlling progression through the eukaryotic cell cycle has come from studies of model organisms, notably Fungi (budding yeast and fission yeast) and Metazoa (frog and fruit fly embryos and mammalian cell lines). Although there are deep functional similarities across all these organisms, suggesting a “universal” control mechanism for eukaryotes (Nurse 1990, 2002), there are differences in the regulators that control the G1/S transition in yeast cells (“start”) and animal cells (“restriction point”). The key decision point for proliferation and development in animal cells occurs in G1 phase. Recent reviews have suggested that the metazoan regulatory proteins E2F, Rb, and Cyclin D may constitute the primitive G1/S control system found in most eukaryotes (Doonan and Kitsios 2009; Cross et al. 2011; Harashima et al. 2013). In support of this hypothesis, key cell-cycle regulators appear to be strikingly conserved across Amoebozoa, including *P. polycephalum.* The ancestral G1/S regulators were seemingly displaced in a fungal ancestor of the yeasts by unrelated but functionally analogous proteins (SBF, Whi5, and Cln3) (Medina *et al.*, unpublished results) (fig. 2). Thus, *P. polycephalum* offers an attractive model and alternative to yeasts for studying the functional dynamics of a mammalian cell-type mitotic cycle.

#### G-Protein Coupled Receptors and Heterotrimeric G-Proteins

The G-protein coupled receptors (GPCRs) are a large and diverse family of transmembrane proteins in Metazoa and other eukaryotes that detect a broad range of physical and chemical stimuli. Based on sequence similarity, GPCRs are subdivided into six families, where members of each family detect different sets of ligands. We found at least 146 GPCRs, with representatives from all families except family 4, the fungal pheromone receptors (supplementary fig. S13, Supplementary Material online). The largest number of GPCRs (42) is present in family 1, the rhodopsin-like receptors, which apart from light receptors also contains receptors for cytokines and neuropeptides. The number of GPCRs in *P. polycephalum* is considerably larger than that for other Amoebozoa (table 3*A*), such as *D. discoideum* with 55 GPCRs (Heidel et al. 2011) and *A. castellanii* with just 35 (Clarke et al. 2013).

**Table 3:** Signaling proteins in Amoebozoa

Class of signaling proteins	*Pp*	*Dd*	*Ac*
A) G-protein coupled receptors			
GPCR family			
1. Rhodopsin-like	42	0	9
2. Secretin-like	23	1	5
3. metabotropic glutamate	38	17	0
4. fungal pheromone	0	0	0
5. frizzled-like	28	25	16
6. cAR-like	14	13	5
All	146	55	35
G-protein subunits			
Alpha	26	12	6
Beta	1	1	n.d.
Gamma	1	1	n.d.
B) Sensor histidine kinases/phosphatases and response regulators			
SHKPs	51	16	48
Response regulators	27*)	5	5
C) Cyclic nucleotide signaling			
cNMP synthesis	64	5	67
Cyclase kinases	43	0	66
Cyclases (other)	21	5	1
cNMP detection	28	5	7
cNMP hydrolysis	11	8	10
D) Protein kinases			
Total	447	295	377
Tyrosine kinases	4	0	21
SH2 domain proteins	18	15	48

**A) G-protein coupled receptors.** Number of members in each of the six families of G-protein coupled receptors (GPCRs) and of subunits of heterotrimeric G-proteins in *Physarum polycephalum* (*Pp*), *Dictyostelium discoideum* (*Dd*), and *Acanthamoeba castellani* (*Ac*). See supplementary figs S13 and S14 for phylogenetic relationships between the *Physarum* proteins.

**B) Sensor histidine kinases/phosphatases and response regulators.** *) Note that the number of response regulators could be inflated by inclusion of sequence fragments that contain response regulators from incompletely assembled SHKPs. See fig. 4 for phylogenies and functional domain architectures of the proteins.

**C) Cyclic nucleotide signaling.** Abundance of nucleotidyl cyclases, cAMP or GMP binding proteins, and cyclic nucleotide phosphodiesterases. See supplementary figs S15 and S16 for phylogenies and functional domain architectures of cyclases and phosphodiesterases.

**D) Protein kinases.** Proteins with S/T, S/T/Y, or Y protein kinase domains were retrieved from an Interproscan of all transcribed coding regions by the presence of the Interpro IPR002290, IPR008271, IPR001245, IPR020635, and/or IPR008266 domains. A total of 29 proteins contained the IPR020635, IPR008266 identifiers for Y protein kinases, but only four of those showed full consensus with validated tyrosine kinase-specific sequences (see fig. 5).

The number of heterotrimeric G-proteins, which are activated by GPCRs as the next step in the signal processing cascade, is also large with 26 G-alpha subunits (supplementary fig. S14, Supplementary Material online), as compared with 12 in *D. discoideum* and 6 in *A. castellanii.* Thus, these gene families have been considerably expanded, possibly to cope with a wealth of different environmental conditions.

#### Blue Light Photoreceptors and Phytochromes

There are distinct classes of chromoproteins that act as photoreceptors in both prokaryotes and eukaryotes in sensing the visible part of the spectrum including near UV: cryptochromes, LOV-domain photoreceptors, rhodopsins, and phytochromes (Heintzen 2012). Photoreceptor domains that are light-sensitive through covalently or non-covalently bound chromophores are often attached to signaling domains that relay the photosensory output to the cellular signaling network. Whereas photobiochemical and physiological functions of quite a number of photoreceptors are well-studied in bacteria, plants, fungi, and animals, this is not the case for the members of the Amoebozoa clade.

**Fig. 3.—:**
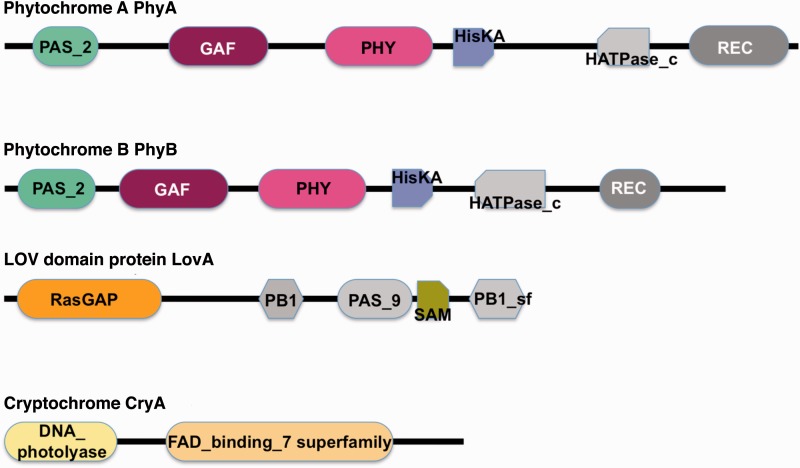
Domain architecture of the predicted photoreceptors encoded in the *P. polycephalum* transcriptome as determined by searching the NCBI Conserved Domain Database (Marchler-Bauer et al. 2015). A description of each domain can be retrieved from the database by searching for the name as it is displayed in this figure. See Supplemental Methods, Results, and Discussion for predicted chromophore-binding sites.

At least two photoreceptors, a phytochrome-like and a blue light photoreceptor, act synergistically in controlling sporulation in *P. polycephalum* in response to far-red and to blue light (Starostzik and Marwan 1995a, 1995b; Lamparter and Marwan 2001) (for details see Supplementary Methods, Results, and Discussion), but these receptors had not been identified at the molecular level. Five members of three classes of putative photoreceptors are expressed by *P. polycephalum*: one cryptochrome and one photolyase, one LOV-domain photoreceptor, and two phytochromes (fig. 3).

##### Cryptochrome and Photolyase

Phypoly-transcript_04617 (*cryA,* cryptochrome-like photoreceptor*; gene locus 1148*) encodes a protein with highest similarity to chicken cryptochrome-2 and other related animal cryptochromes. Downstream of the DNA-photolyase and FAD-binding domain of *cryA* is a short C-terminal part of approximately 100 amino acids encoded in the genomic sequence that is missing in Phypoly-transcript_04513 (gene locus 3553 and 3554), which shares greatest sequence similarity with DNA-photolyases and is designated as *plyA* (for photolyase A-type photoreceptor) and is presumably a DNA-photolyase rather than a sensory blue light photoreceptor. However, the physiological role of both *plyA* and *cryA* in *P. polycephalum* remains to be established.

##### LOV-Domain Photoreceptor

The Phypoly-transcript_01902 (*gene locus 13045*) encodes a protein with a domain architecture composed of RasGAP, PB1, PAS_9, SAM, and PB1 superfamily domains (fig. 3). BLAST analysis revealed the highest similarity to the phototropin-2 photoreceptor from *Arabidopsis thaliana* (P93025) and to other LOV-domain-carrying phototropins from plants and bacteria as well as to the fungal white-collar 1 (WC1) blue light photoreceptor from *Neurospora.* The PAS_9 domain of the predicted *P. polycephalum* LovA protein contains the highly conserved NCRFLQ motif with the cysteine residue serving as a potential chromophore-binding site embedded in additional highly conserved residues (supplementary fig. S18, Supplementary Material online) that are involved in chromophore binding and crucial for the photochemical properties of phototropin-type photoreceptors (Heintzen 2012). The domain composition of the predicted protein and the highly conserved residues for chromophore binding in the PAS_9 domain suggests that LovA may act as an unconventional blue light photoreceptor that integrates, modulates, and/or relays multiple signals and might even bind DNA through its PAS_9 domain.

##### Phytochromes

Originally considered to be plant-specific photoreceptors, phytochromes regulate many aspects of metabolism, motility, gene regulation, and development (Heintzen 2012). Even before prokaryotic genome projects uncovered the early evolutionary origin of phytochromes, phytochrome-like photoreceptors were discovered in *Aspergillus* (Mooney and Yager 1990) and *P. polycephalum* (Starostzik and Marwan 1995b; Lamparter and Marwan 2001). Two phytochrome genes, *phyA* and *phyB*, are expressed in *P. polycephalum*, partially encoded by transcripts 20261 (gene locus 28349) and 03416 (gene locus 5996), respectively. Although the sequence identity between PhyA and PhyB is only 33.8%, the two proteins share a similar domain architecture with the phytochrome-type PAS_2, GAF, PHY domain arrangement at the N-terminus, and a C-terminal hybrid kinase-like part, composed of HisK, HATPase, and REC domains (fig. 3). A BLAST search of the PAS_2, GAF, PHY domain portion of the proteins against the UniProt database revealed closest similarity to *Nostoc* PHYA (Q9LCC2) and four other bacterial phytochromes, followed by plant phytochromes. Sequence alignment with the most similar phytochromes suggests that the chromophore-binding site may be a cysteine close to the N-terminus that is conserved between *P. polycephalum* PhyA and PhyB and various bacterial phytochromes, whereas the cysteine of the CHxxYxxNMG motif that serves as a chromophore-binding site in plant phytochromes is replaced by valine in the two *P. polycephalum* proteins (supplementary fig. S20, Supplementary Material online). Whether the photochemistry of the two phytochromes is identical, or one of them may be specifically synthesized in the dark in its far-red light absorbing P_fr_ form (Lamparter and Marwan 2001) to trigger sporulation (see Supplementary Information) remains to be established. The deletion upstream of the conserved PASDIPPQARRL motif in PhyA, as compared with PhyB, could conceivably cause an according functional difference.

#### Sensor Histidine Kinases/Phosphatases

**Fig. 4.—:**
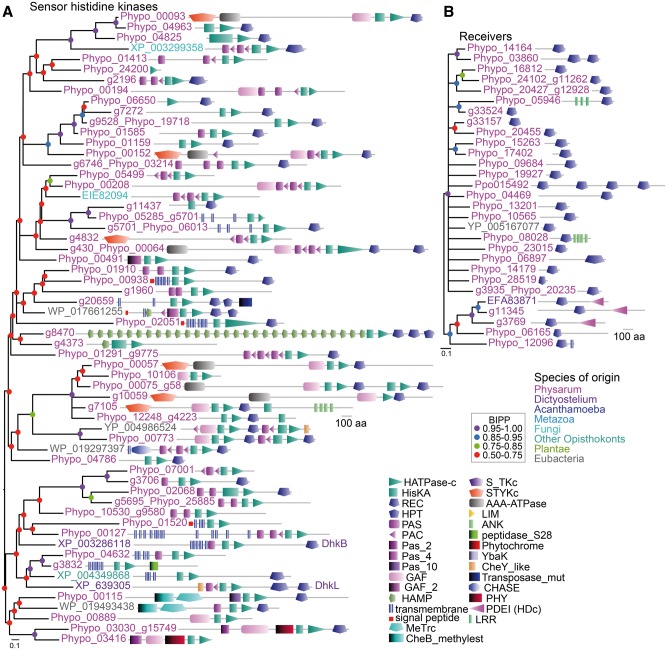
Sensor histidine kinases/phosphatases and phosphorelay receivers. (*A*) *Sensor histidine kinases/phosphatase.* The *P. polycephalum* genome was queried by BLAST with all 15 Dictyostelid SHKPs, plant and fungal SHKPs, and the most divergent prokaryote SHKPs, while transcribed proteins were queried for the presence of Interpro domains IPR003661 and IPR003594 for the HisKA and HATPase-c moieties of SHKPs, respectively. The retrieved sets largely overlapped, but the transcriptome (Phypo identifiers in the tree) contained both the largest number and more complete SHKPs. Sequences were first aligned with all their closest relatives in other organisms, as identified by BlastP of the GenBank non-redundant sequences. However, in a phylogeny constructed from this alignment, most of the outgroup sequences clustered together. Their contribution therefore was reduced to the ones with greatest identity to individual clusters of *P. polycephalum* SHKPs. A second phylogeny was constructed from these outgroup and *P. polycephalum* sequences, using only segments encompassing the HisKA, HATPase-c, and receiver domains, which are common to all or most retrieved SHKPs. The tree was annotated with the functional domain architecture of the proteins. The methods used for sequence alignment, alignment editing, and Bayesian phylogenetic inference are the same as described in the legend to supplementary fig S13. Protein tags are color-coded to reflect the species of origin as shown, and Bayesian posterior probabilities of tree nodes are represented by colored dots. (*B*) *Receivers.* Putative effectors for SHKP activated signaling were identified by query of genome and transcriptome sequences with receiver/response regulator protein sequences or the Interpro identifier IPR001789 for this domain, respectively, leaving out all proteins that also contained HisKA or HATPase-c domains. Query for outgroup sequences mainly retrieved SHKPs, which also contain a receiver domain, and *Dictyostelium* RegA. RegA and one other none-SHKP outgroup sequence (YP_005167077) were aligned with the *P. polycephalum* sequences, and a phylogenetic tree was constructed by Bayesian inference as described above. The tree was decorated with protein domain architectures.

Other important receptors for external stimuli are the sensor histidine kinases/phosphatases (SHKPs), which are very abundant in prokaryotes and also present in significant numbers in plants, fungi, and Amoebozoa, but not in Metazoa (Wolanin et al. 2002). SHKPs initiate forward or reverse relay, respectively, of a phosphoryl group to/from a conserved aspartate in a response regulator. The response regulator subsequently regulates the activity of an effector, such as an enzyme or transcription factor. The *P. polycephalum* genome contains ∼51 SHKPs with a large variety of different functional domain architectures (fig. 4*A*). Many of these SHKPs contain small molecule or light-sensing domains, such as the GAF, PAS/PAC, or phytochrome domains, which are likely to detect stimuli and activate phosphotransfer, whereas others contain protein kinase or AAA-ATPase domains. These domains could be downstream effectors, which are regulated by phosphorelay. The number of SHKPs in *P. polycephalum* is about equal to that in *A. castellanii* and three times larger than that in *D. discoideum* (table 3*B*), but there are up to five times more response regulators in *P. polycephalum* than in either *D. discoideum* or *A. castellanii.*

Whereas most *P. polycephalum* response regulators have no additional features, two have leucine-rich repeats and two have a phosphodiesterase domain and are closely related to the *D. discoideum* cAMP phosphodiesterase RegA (fig. 4*B*). In *Dictyostelids,* RegA is a negative regulator of encystation and sporulation, which both require activation of PKA by cAMP (Shaulsky et al. 1998; Du et al. 2014). In *P. polycephalum,* sporulation is induced by light and this induction was shown to be mediated by a phytochrome-type receptor (Starostzik and Marwan 1995b). The light-sensing part of this photoreceptor is likely to be the phytochrome domain that is present in two *P. polycephalum* SHKPs (see below; fig. 4*A*), which suggests that the stimuli that control sporulation in *P. polycephalum* also act on intracellular cAMP levels by regulating RegA activity.

#### Cyclic Nucleotide Signaling

The cyclic nucleotides cAMP, and to a lesser extent cGMP, are widely used as intracellular second messengers for a broad range of external stimuli in all domains of life. cAMP is particularly important for Dictyostelid development, where it regulates encystation, progression through multicellular development, maturation of spore and stalk cells, and maintenance of spore and cyst dormancy by acting on PKA (Schaap 2011). In addition, Dictyostelids use cAMP extracellularly as a chemoattractant to organize fruiting body morphogenesis in all species, and aggregation in a subset that contains the model organism *D. discoideum* (Alvarez-Curto et al. 2005). In contrast, there are only a few documented roles for cAMP or cGMP in *P. polycephalum.* cGMP plays a role in the induction of sporulation in *P. polycephalum* (Golderer et al. 2001), and there is sporadic evidence of roles for cAMP in cell division, motility, and gravity sensing (Kuehn 1972; Block et al. 1998; Matveeva et al. 2012).

To evaluate the prevalence of cAMP and cGMP signaling in *P. polycephalum*, we investigated the presence of the cyclases, binding proteins, and phosphodiesterases that, respectively, synthesize, detect, or degrade cyclic nucleotides.

To our surprise, the number of cyclase and cyclic nucleotide binding proteins in *P. polycephalum* by far surpasses that in *D. discoideum.* In fact, *P. polycephalum* has 64 nucleotidyl cyclases against 5 in *D. discoideum. A. castellanii* has even more (67), but 66 of these cyclases belong to a massively amplified family of transmembrane proteins that contain a cyclase domain, flanked by two protein kinase domains. *P. polycephalum* has a family of 43 of those proteins, suggesting a common requirement for amplification of these cyclases in some Amoebozoa. *P. polycephalum* has in addition 21 cyclases with highly variable functional domain architectures (supplementary fig. S16, Supplementary Material online). This set is dominated by proteins with two sets of six transmembrane domains that flank two cyclase domains. This is the configuration of most mammalian adenylate cyclases and of *D. discoideum* ACA and GCA. In addition, there are cyclases with GAF and PAS/PAC domains and with domains involved in actin remodeling, ubiquitination, or calcium export. *P. polycephalum* also has a close homolog of *D. discoideum* SGC, a guanylate cyclase that is involved in chemotaxis, and it has a close homolog of *D. discoideum* and *A. castellanii* AcrA, an adenylate cyclase that is essential for spore maturation in *D. discoideum* (Loomis 2014).

The number of cyclic nucleotide phosphodiesterases in *P. polycephalum* is about equal to that in *D. discoideum* and *A. castellanii* (supplementary fig. S15, Supplementary Material online). However, the number of proteins predicted to be capable of binding cAMP or cGMP is truly astonishing and exceeds the numbers in *D. discoideum* and *A. castellanii* by five- and fourfold, respectively (table 3). Whereas the majority of these proteins have only cNMP binding domain(s), there are also homologs of the *D. discoideum* cGMP binding proteins GbpC and GbpD, which additionally have protein kinase, RasGEF, and DEP domains (supplementary fig. S17, Supplementary Material online). There is also a homolog of *D. discoideum* PdeE, in which an intrinsic Lactamase_B domain acts as a cAMP phosphodiesterase. In addition, there are putative cNMP binding proteins with additional RhoGEF, VWA, and P2X receptor domains that are not present in other organisms. The multitude and variety of cNMP binding domains and nucleotidyl cyclases in *P. polycephalum* indicates that cyclic nucleotide signaling is likely to play a very dominant role in its physiology and development.

#### Protein Kinases

The protein kinases that modify the function of downstream effector proteins by phosphorylation of serine/threonine (S/T) or tyrosine (Y) residues are the most common intermediates of signal processing cascades. Humans have 518 protein kinases and even yeast has over a hundred (Hanks 2003). Inspection of the *P. polycephalum* transcriptome revealed the presence of a total of 447 proteins with S/T, Y, or S/T/Y (dual specificity) kinase domains (see Supplemental Methods, Results, and Discussion and table 3 for further details). This is 1.5- and 1.2-fold more than in *D. discoideum* and *A. castellanii*, respectively, and, therefore, a more modest increase than observed for the other signal transduction proteins described above.

**Fig. 5.—:**
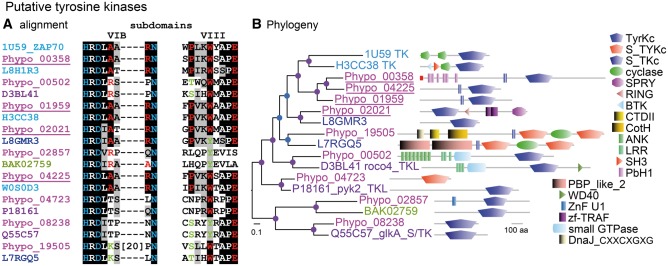
Alignment and phylogeny of putative tyrosine kinases. The sequences of the most consensual *P. polycephalum* tyrosine kinases were aligned with those of their closest homologs in other species. (*A*) Section of the alignment that is essential for peptide substrate recognition, with residues essential for all protein kinases in blue text, for tyrosine kinases in red text, and for S/T or S/T/Y kinases in green. The identifiers of *P. polycephalum* proteins with full tyrosine kinase consensus are underlined. (*B*) Bayesian phylogeny constructed from the full kinase domain alignment, annotated with protein domain architectures. Species protein tags and node probabilities are color coded as in fig. 4. The methods described in the legend to supplementary fig S13 were used for phylogenetic inference. For *D. discoideum* homologs with confirmed substrate specificity, gene names and kinase designation (TK and TKL:tyrosine; S/TK serine/threonine) follow the protein tags.

**Fig. 6.—:**
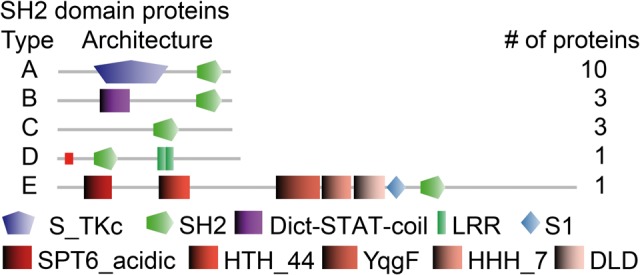
Proteins with SH2 domains. Proteins that harbored the Interpro IPR000980 SH2 domain were retrieved from an Interproscan of all transcribed coding sequences. The SH2 domain is too small for meaningful sequence alignment-based phylogeny reconstructions and proteins are therefore classified by their protein domain architecture. The identifiers of the proteins of each type are as follows: A: Phypo_00702, Phypo_02696, Phypo_03176, Phypo_05058, Phypo_06094, Phypo_06144, Phypo_06676, Phypo_06719, Phypo_6732, Phypo_07245; B: Phypo_02646, Phypo_02863, Phypo_01571; C: Phypo_10425, Phypo_14943, Phypo_03389; D: Phypo_05516; E: Phypo_00177.

The initial analysis based on Interpro identifiers for substrate specificity revealed the presence of 29 tyrosine kinases, but a more in-depth comparison with sequence signatures of validated tyrosine kinases revealed that only four of those contain all essential residues for tyrosine substrate recognition (fig. 5). Three of these proteins contain a single transmembrane domain that provides them with structural similarity to metazoan receptor tyrosine kinases (RTKs), which are the targets for a broad range of peptide growth factors, hormones, and cytokines. In Metazoa, tyrosine phosporylated substrates become binding sites for SH2 domain proteins, which further transduce the response (Liu and Nash 2012). *P. polycephalum* has 18 SH2 domain proteins (fig. 6), of which three are similar to *D. discoideum* and metazoan STAT transcription factors, whereas *D. discoideum* and *A. castellanii* have 15 and 48 SH2 domain proteins, respectively.

Tyrosine kinases play very dominant roles in metazoan embryogenesis and adult physiology, and were until recently considered to be a hallmark of metazoan evolution because they were present only in Metazoa and one of its unicellular allies, a choanoflagellate (King and Carroll 2001) and absent from yeast, plants, and *D. discoideum* (Lim and Pawson 2010). This notion was overturned by the identification of some tyrosine kinases outside of Opisthokonta (Suga et al. 2012) and the presence of 21 tyrosine kinases in *A. castellanii* (Clarke et al. 2013), a member of Lobosa, one of the two major subdivisions of Amoebozoa. *P. polycephalum* and *D. discoideum* reside in the other subdivision Conosa (Schilde and Schaap 2013). The presence of tyrosine kinases in both subdivisions of Amoebozoa, in their sister group Opisthokonta, and in other eukaryote divisions indicates that tyrosine kinases were likely present in the LCA to all eukaryotes and were selectively lost from many phyla. The more widely distributed tyrosine kinase-like enzymes, for example, *D. discoideum* Pyk2, which can also phosphorylate SH2 domain tyrosines (Araki et al. 2014), have probably taken over their role in these phyla.

## Conclusions

*P. polycephalum* has for long been a classic model organism in cell biology. The accessibility of both genome sequence and extensive transcriptome data sets strongly enhances its usefulness as a model system. The availability of the transcriptome of amoebae and plasmodia at different stages of development now permits reverse genetic approaches through identifying mutants in genes of interest in mutant libraries obtained by chemical mutagenesis of amoebal cells. In addition, the transcriptome sequences provide the basis for quantitative proteomic approaches by mass spectrometry. Our analysis highlights a wealth of interesting molecular features and will help in enabling work at the molecular level in combining the well-established classic genetics with second-generation sequencing approaches.

The results of the *P. polycephalum* sequencing project described here provide interesting information with respect to the evolution of signaling systems in the Amorphea branch of the eukaryotes. Virtually all aspects discussed here characterize *P. polycephalum* as an organism with higher molecular complexity than other sequenced Amoebozoa. This becomes most obvious for cellular signaling through the extensive use of two-component signaling systems in parallel with RTK signal transduction. Other important aspects such as cell cycle regulation, cytoskeletal motor proteins, and the enzymes of the pteridine metabolism point in the same direction. Together with recent findings on *A. castellanii* (Clarke et al. 2013), our results indicate that the molecular evolution of these features, notably signaling through RTKs, which has previously been considered a hallmark of multicellularity of animals, is deeply rooted in the Amorphea and has been secondarily lost in other Amoebozoa like *D. discoideum* and in the fungi. Another interesting feature of *P. polycephalum* is the occurrence of photoreceptors: two bacterial-type phytochromes, not found in other Amoebozoa, a phototropin-like LOV-domain blue light photoreceptor, and a cryptochrome. In conclusion, among the unicellular genetic model organisms, *P. polycephalum* displays many features of animal cells and, with respect to its molecular complexity, the cross-talk of signaling molecules, and the resulting dynamic behavior. As such, it is anticipated that it will serve as an interesting model system contributing complementary information for the study of mammalian and other animal cells. In combination with its ability to form multinucleate giant plasmodia, this provides unique options for investigating fundamental biological processes in individual cells.

## Supplementary Material

Supplemental Methods, Results, and Discussion with figures S10, S13–S18, S20, S21, table S2–S4 and Supplementary Spreadsheets including Supplementary Spreadsheets 1 and 2 are available at Genome Biology and Evolution online (http://www.gbe.oxfordjournals.org/).

Supplementary Data
