# Supplementary material for: The Physarum polycephalum Genome Reveals Extensive Use of Prokaryotic Two-Component and Metazoan-Type Tyrosine Kinase Signaling
Source: Genome Biol Evol. 2015 Nov 27;8(1):109–25. doi: 10.1093/gbe/evv237 (PMC4758236; doi:10.1093/gbe/evv237)
Supplement: Supplementary Data [file supp_8_1_109__index.html]

The Physarum polycephalum Genome Reveals Extensive Use of Prokaryotic Two-Component and Metazoan-Type Tyrosine Kinase Signaling — Supplementary Data 

# The *Physarum polycephalum* Genome Reveals Extensive Use of Prokaryotic Two-Component and Metazoan-Type Tyrosine Kinase Signaling

## Supplementary Data

files

- Supplementary Data - zip file
