# Supplementary material for: The Physarum polycephalum Genome Reveals Extensive Use of Prokaryotic Two-Component and Metazoan-Type Tyrosine Kinase Signaling
Source: Genome Biol Evol. 2015 Nov 27;8(1):109–25. doi: 10.1093/gbe/evv237 (PMC4758236; doi:10.1093/gbe/evv237)
Supplement: Supplementary Data [file supp_evv237_evv237-suppl_data.zip › evv237-Schaap_Supplementary_File_1.pdf]

Supplementary File 1 – Supplemental Methods, Results, and Discussion

Schaap *et al.*: The *Physarum polycephalum* genome reveals extensive use of prokaryotic two-component and metazoan-type tyrosine kinase signaling

Table of Contents

**1.**	**Preparation of DNA for Genome Sequencing**	5
	1.1 Strains and culture conditions	5
	1.2 Estimation of the nuclear DNA content of LU352 amoebe by flow cytometry	5
	1.3 Isolation of nuclear DNA for genome sequencing	5
**2.**	**Preparation of RNA for Transcriptome Sequencing**	7
	2.1 Isolation of amoebal RNA	7
	2.2 Isolation of plasmodial RNA, cDNA synthesis, and sequencing	7
	2.3 De novo assembly of sequences and generation of the reference transcriptome	9
**3.**	**Intron Size Distribution and Domains**	10
	3.1 Intron sizes	10
	3.2 Domains	10
**4.**	**Transposable Elements**	17
	4.1 DNA transposons	17
	4.2 LTR retrotransposons	17
	4.3 Non-LTR retrotransposons	19
**5.**	**Small Non-coding RNAs**	21
**6.**	**MicroRNA and Associated mRNA Targets**	22
	6.1 Overview	22
	6.2 Materials & Methods	24

7. Pteridine Metabolism 25

7.1 Background 25

7.2 Methods 25

7.3 Results 25

8. Cell cycle regulation 33

8.1 Methods 33

8.2 P. polycephalum *cell cycle regulation compared to other organisms* 33

9. G-protein Coupled Receptors and Heterotrimeric G-proteins 37

10. Sensor Histidine Kinases and Phosphatases 40

11. Cyclic Nucleotide Signaling 41

11.1 Cyclic nucleotide synthesis: adenylate- and guanylate cyclases 41

11.2 Cyclic nucleotide detection: cNMP binding domains 43

11.3 Cyclic nucleotide degradation: phosphodiesterases 45

12. Protein Kinases 46

13. Photoreceptors 48

14. DYW-type PPR Proteins 52

15. Phylogenetic Position of *P. polycephalum* Within the Eukaryotes 53

16. Appendix A: Origin of LU352 and Other Strains Used in this Study 55

17. Appendix B: Rules for *P. polycephalum* Genetic Nomenclature 61

18. References 67

Supplementary File 1 Figures List

**Figure S1**	Genomic DNA from *P. polycephalum* amoebae	6
**Figure S2**	Intron size distribution in *P. polycephalum*	10
**Figure S3**	Alignments of RT, RNH, and IN domains of *P. polycephalum* LTR retrotransposons	18
**Figure S4**	Phylogenetic analysis of *P. polycephalum* LTR retrotransposons	19
**Figure S5**	Phylogenetic analysis of *P. polycephalum* non-LTR retrotransposons	20
**Figure S6**	Depiction of mature miRNA sequence density	23
**Figure S7**	Identification of a novel P. *polycephalum* mature miRNA (novel-ppo-miR-8) predicted to bind Dynein Heavy Chain 1 (cytoplasmic) - like mRNA	23
**Figure S8**	Pathways from GTP to pteridine compounds	26
**Figure S9**	Transcripts for Molybdenum cofactor biosynthesis protein 2	27
**Figure S10**	Riboflavin biosynthesis with two trifunctional proteins in *P. polycephalum*	28
**Figure S11**	Molecular phylogeny of cyclins	34
**Figure S12**	Molecular phylogeny of cell division kinases	35
**Figure S13**	G-protein coupled receptor families	38
**Figure S14**	Heterotrimeric G-proteins	39
**Figure S15**	Cyclic nucleotide phosphodiesterases	41
**Figure S16**	Nucleotidyl cyclases	42
**Figure S17**	Cyclic nucleotide binding domains	44
**Figure S18**	Putative *P. polycephalum* phototropin PtnA chromophore binding region	49
**Figure S19**	Predicted sequence of *P. polycephalum* cryptochrome CryA	50
**Figure S20**	Alignment of the predicted *P. polycephalum* phytochromes PphA and PphB sequences	51
**Figure S21**	DYW-type PPR proteins in *P. polycephalum*	52
**Figure S22**	Alignment from a concatenated data set of 30 highly conserved proteins	53
**Figure S23**	Pedigree of *P. polycephalum* strain LU352	58
**Figure S24**	Pedigree of *P. polycephalum* albino strains LU897 and LU898	60

Supplementary File 1 Tables List

**Table S1**	Strains used, types of RNA samples prepared thereof, and summary of sequencing and transcriptome assembly results	8
**Table S2**	Domains not found in D. discoideum but in *P. polycephalum* (PP) and A. castellanii (AC)	11
**Table S3**	Domains overrepresented in or restricted to *P. polycephalum*	12
**Table S4**	Transposable elements comprise approximately 1.3% of the *P. polycephalum* genome	21
**Table S5**	Pteridine metabolic open reading frames in *P. polycephalum*	29
**Table S6**	Core cell cycle regulators across eukaryotic genomes	36
**Table S7**	Cell division kinases across eukaryotic genomes	36
**Table S8**	Set of *D. discoideum* genes highly conserved among the eukaryotes used for calculation of the phylogenetic tree shown in Fig. S22	54
**Table S9**	Amoebal parents of plasmodial strains used for transcriptome sequencing	56
**Table S10**	Genotype and origin of *P. polycephalum* progenitor strains of LU352	57
**Table S11**	Genotype and origin of *P. polycephalum* progenitor strains of the albino strains LU897 and LU898	59

Supplementary Spreadsheets

(available at Genome Biology and Evolution online)

**Supplementary Spreadsheet 1**	Small Noncoding RNAs
**Supplementary Spreadsheet 2**	Cytoskeleton Proteins
**Supplementary Spreadsheet 3**	MicroRNAs
**Supplementary Spreadsheet 4**	MicroRNA Targets
**Supplementary Spreadsheet 5**	MicroRNA Target Prediction
**Supplementary Spreadsheet 6**	Cell Cycle Regulators
**Supplementary Spreadsheet 7**	Kinases

1. Preparation of DNA for Genome Sequencing

1.1 Strains and culture conditions

Haploid, uninucleate amoebae of strain LU352 which were kindly provided by Roger Anderson, were first plated on a bacteria lawn, and then subcultured axenically in semi-defined liquid medium in the presence of antibiotics as described (Dee, et al. 1989b). Diploid, multinucleated microplasmodia of strain Tu291 were grown under standard conditions in liquid shaken cultures (Bénard, et al. 2007). Because of frequent genetic polymorphism in diploid plasmodial cells, haploid mononucleate, axenically grown amoebae of strain LU352 (Dee, et al. 1989b) (Fig. S1A) were chosen for genome sequencing. Axenic growth avoided the risk of contamination of amoebal DNA preparations with DNA from food bacteria on which amoebae may grow non-axenically.

1.2 Estimation of the nuclear DNA content of LU352 amoebe by flow cytometry

Nuclei were isolated from either amoebae or microplasmodia as previously described (Bénard, et al. 2007; Maric, et al. 2003). Nuclei were counted with a Mallassez cell and 10^7 nuclei were fixed with 3 volumes of ethanol, digested with 0.1 mg RNaseA and stained with 0.1 mg propidium iodide (Bénard, et al. 2007). Samples were analyzed with a FACSortTM (BD Biosciences, San Jose, CA, USA) (Fig. S1B). Cytometry analyses indicated a homogenous DNA content of amoebael nuclei (Fig. S1B, left panel). Without gating, the main peak was flanked by debris on its left and doublets on its right. To quantify the amount of amoebal DNA per nucleus, nuclei isolated from microplasmodia for which the DNA content had been previously quantified as 4C = 1.2 pg per nuclei in G2 phase (Kubbies and Pierron 1983) were added as an internal standard to the preparation of amoebal nuclei. Amoebal and plasmodial nuclei were mixed at different ratios prior to propidium iodide staining and analysed by flow cytometry (Fig. S1B right panels). Amoebal DNA contents were in the range of half the amount of DNA in microplasmodial nuclei, indicating that the main peak of amoebal DNA corresponds to the DNA content of the haploid, G2-phase genome of *P. polycephalum*. These data indicate that the genome of LU352 has a C-value of 0.3pg which corresponds to $3x10^8$ bp of DNA.

1.3 Isolation of nuclear DNA for genome sequencing

Approximately $2x10^9$ axenically grown amoebae of strain LU352 were pelleted. Nuclei were isolated, lysed and nuclear proteins digested with proteinase K for the isolation of genomic DNA which was performed as previously described (Maric, et al. 2003). Genomic DNA was purified on a CsCl equilibrium gradient in order to remove contaminating mitochondrial DNA, ad CsCl was removed by dialysis. DNA concentration was estimated by measuring optic density (260 nm) of the solution. DNA quality was tested by agarose gel electrophoresis (Fig. S1C) and Southern blot analyses revealed expected patterns of hybridization with specific probes (data not shown).

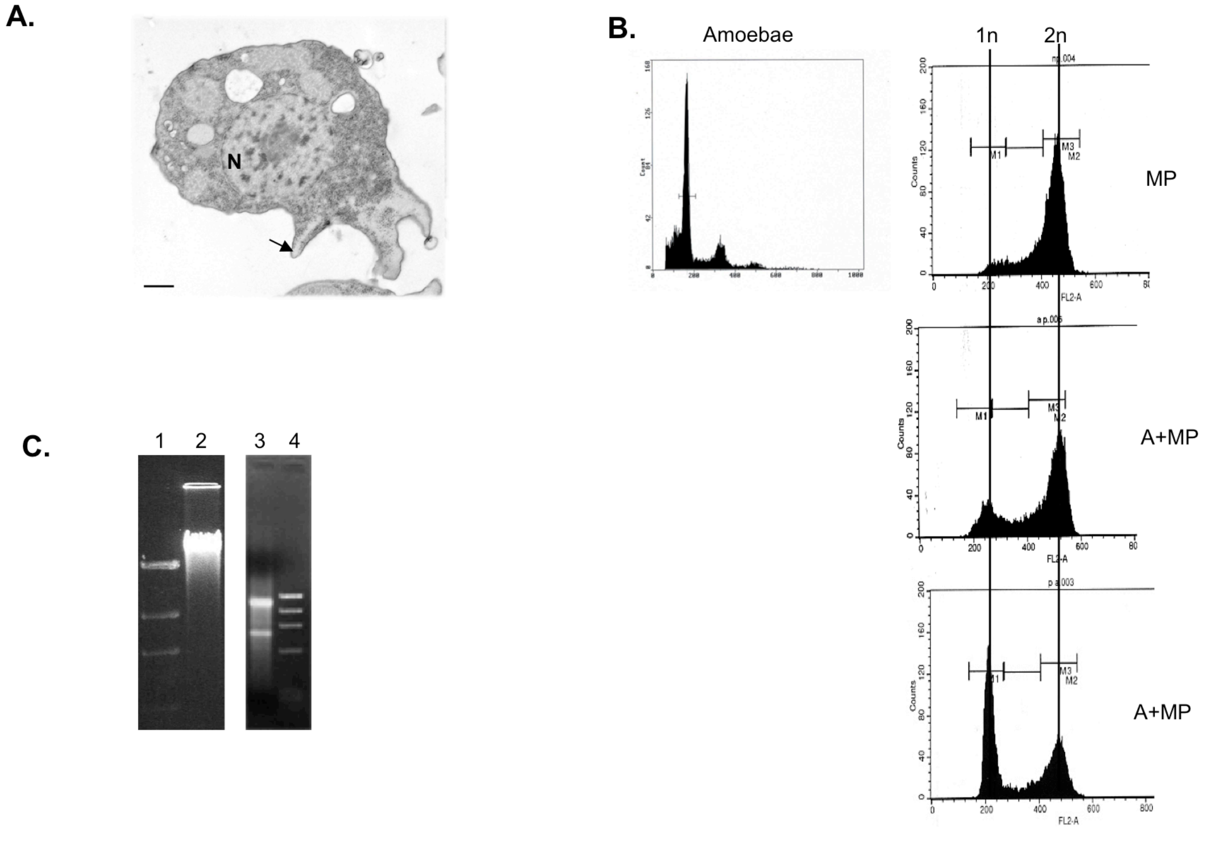

Figure S1. Genomic DNA from *P. polycephalum* amoebae.

A. Electron microscope micrograph of an amoeba of strain LU352. Amoebae were grown in axenic medium and an aliquot was harvested for fixation in 1.6% glutaraldehyde and embedded in Epon. Ultrathin sections were stained with 4% uranyl acetate. Amoebae are uninucleate cells that are able to move by using cytoplasmic extensions (arrow). N, nucleus. Bar, 1 µm.

B. Flow-cytometry analyses. Nuclei were isolated from amoaebae or microplasmodia and their DNA content was analyzed by flow cytometry. Left: amoebael nuclei represented a homogeneous population with respect to their DNA content. Right: histograms of nuclei from microplasmodia (MP, upper panel), or mixtures of nuclei from amoebae and microplasmodia (A+MP, lower amount of amoebael nuclei: mid panel). Microplasmodial nuclei were mostly in G2 phase. Amoebael nuclei with a haploid set of chromosomes displayed DNA contents of approximately half of the amount of DNA in microplasmodial nuclei.

C. Agarose gel analysis of *Physarum* genomic DNA and total RNA. Left,1.5 µg DNA of amoebae were loaded on a 0.8% agarose gel stained with ethidium bromide. The molecular weight marker is a *Hind*III digest of λ phage (lane 1). The genomic DNA displays high molecular weight (lane 2). This DNA preparation was used for genome sequencing. Right, a sample of RNA preparation was heated at 65°C for 3 min and loaded on a 1% agarose neutral gel. Ethidium bromide staining revealed two main bands corresponding to ribosomal RNA (lane 3). The molecular weight marker is *Hae*III-digested ΦX174 DNA (lane 4).

2. Preparation of RNA for Transcriptome Sequencing

2.1 Isolation of amoebal RNA

Total RNA was also prepared from axenically grown amoebae for cDNA sequencing. Total RNA was extracted from amoebae by solubilization in guanidium isothiocyanate and centrifugation through a CsCl cushion as described (Chirgwin, et al. 1979). Pellets of RNA were dried briefly and dissolved in 300 µl sterile water. Following addition of sodium acetate, RNA was ethanol precipitated. A sample of RNA preparation was pelleted by centrifugation, dissolved in water and used for control gel electrophoresis and quantification. The quality of RNA preparation was checked on agarose gel (Fig. S1C).

2.2 Isolation of plasmodial RNA, cDNA synthesis, and sequencing

Isolation of RNA from plasmodial cells was done as previously described (Barrantes, et al. 2012) or performed using the RNeasy Plant Mini kit (Qiagen). Strains used and types of RNA samples prepared thereof are given in Table S1. For deep sequencing of samples #1-9 (Table S1), synthesis of cDNA and Illumina sequencings were performed as a commercial service at vertis Biotechnologie (Freising-Weihenstephan, Germany). Briefly, poly-A RNA was prepared from the total RNA samples and fragmented using ultrasound (4 pulses of 30 seconds at 4°C). After treatment with tobacco acid pyrophosphatase (TAP), RNAs were rephosphorylated with a polynucleotide kinase (PNK). The RNA fragments were then poly(A)-tailed using a poly(A) polymerase, followed by the ligation of a RNA adapter to the 5' ends. First-strand cDNA synthesis was carried out with an oligo(dT)-adapter primer and the Moloney Murine Leukemia Virus (M-MLV) reverse transcriptase. The obtained cDNAs were amplified by PCR to about 30 ng/µl using a high fidelity DNA polymerase, and the PCR products were purified using the Agencourt AMPure XP kit (Beckman Coulter Genomics) and analyzed by capillary electrophoresis on a Shimadzu MultiNA microchip system. cDNA samples were pooled in approximately equivalent amounts, and fragments in the range of 200 to 500 bp were fractionated from preparative agarose gels for single-end sequencing using the Illumina HiSeq 2000 system (Bentley, et al. 2008). For deep sequencing of sample #10 (Table S1), we constructed cDNA libraries using a TruSeq RNA Sample Preparation kit (Illumina) and performed Illumina sequencing. Briefly, 1 µg of total RNA were used for polyA selections with RNA purification beads. The cDNA library was purified by AMPure (Beckman coulter) by using a magnetic stand. The amount of cDNA library was quantified using QuantiFluor dsDNA System (Promega). Both the 5’ and 3’ ends of the cDNAs were sequenced using an Illumina Genome Analyzer IIx with a paired end module for 64 cycles (Illumina). For deep sequencing of sample #11 (Table S1), synthesis of cDNA and 454-sequencing was performed as a commercial service at Filgen (Aichi, Japan). Briefly, first-strand cDNA was synthesized using poly-A RNA prepared from the total RNA and N6 randomized primer. Then 454 adapter A and B were ligated to the 5' amd 3' ends of the cDNA. The cDNA was finally amplified with PCR (18 cycles) using a proof reading enzyme. Normalization was carried out by one cycle of denaturation and reassociation of the cDNA, resulting in N1-cDNAs. Reassociated ds-cDNA was separated from the remaining ss-cDNA (normalized cDNA) by passing the mixture over a hydroxylapatite colum. After hydroxylapatite chromatography, the ss-cDNA was amplified with 11 PCR cycles. The cDNA in the range of 500 to 700 bp were fractionated from preparative agarose gels for Titanium sequencing using 454 GS FLX system (Roche).

Table S1. Strains used, types of RNA samples prepared thereof, and summary of sequencing and transcriptome assembly results. Strains PHO1, PHO3, and PHO26 are mutants with strongly reduced ability to sporulate in response to far-red light (spo^{-}) that were derived from WT31. MA275 (Kirouac-Brunet, et al. 1981; Schedl, et al. 1984) and DP246 (Moriyama and Kawano 2003) are Wis-2 progeny strains. AI35 is a progeny strain produced by crossing a and i strains (Moriyama and Kawano 2003). RNA-seq reads are indicated by millions of reads, and assemblies and clustering specify the number of generated cDNA sequences. Accessions specify to the European Nucleotide Archive study entries (Leinonen, et al. 2011) or DDBJ Sequence Read Archive. For further details on the origin of strains see Appendix A.

#	Strain	Cell type	Physiological state and sample composition	Study Accession(s)	Reads (millions)	*De novo* Assembly	cDNA Clustering	Re-Assembly	Total Mbp
1	WT31	plasmodia	Sporulation competent and committed to sporulation	ERP001220	77.07	909505	289896	82120	27.25
2	WT31 x MA275	plasmodia	Sporulation competent and committed to sporulation	ERP006235	146.39	438250	197124	75468	21.62
3	LU879 x LU898	plasmodia	Sporulation competent and committed to sporulation	SRP009381 ERP006235	115.24	860888	319306	84498	66
4	PHO1	plasmodia	Non-sporulating mutant of WT31; starved and far-red light-induced	ERP006235	60.94	591713	218979	70628	24.49
5	PHO26	plasmodia	Non-sporulating mutant of WT31; starved and far-red light-induced	ERP006235	134.26	358887	191916	110689	27.23
6	PHO3	plasmodia	Non-sporulating mutant of WT31; starved and far-red light-induced	ERP006235	4.6	25263	11291	10121	1.33
7	PHO1 x MA275 progeny clones	plasmodia	Pool of plasmodial RNAs of apogamic (haploid) sporulating segregants of the cross PHO1 x MA275	ERP006235	67.76	270386	118404	53148	17.76
8	PHO26 x MA275 progeny clones	plasmodia	Pool of plasmodial RNAs of apogamic (haploid) sporulating segregants of the cross PHO26 x MA276	ERP006235	93.37	718434	387419	126341	92.07
9	PHO3 x MA275 progeny clones	plasmodia	Pool of plasmodial RNAs of apogamic (haploid) sporulating segregants of the cross PHO3 x MA277	ERP006235	88.33	786825	417216	146625	103.71
10	AI35 x DP246	plasmodia	Mircoplasmodia grown in sumbersed culture	DRA004071	51.24	34630	-	-	32.77
11	AI35 x DP246	plasmodia	Mircoplasmodia grown in sumbersed culture	DRA004071	0.54	21951	-	-	13.91

2.3 De novo assembly of sequences and generation of the reference transcriptome

Outputs from the Illumina sequencing runs of samples #1-9 (Table S1) were decoded and trimmed for quality, employing the fastq and fastx toolkits respectively (Gordon 2008; Jones 2011). These quality-filtered RNA-seq datasets were then assembled *de novo* using velvet (version 1.1.05; (Zerbino and Birney 2008)), and the transcriptomes reconstructed with the oases tool (version 0.1.22; (Schulz, et al. 2012)). To avoid redundancy between samples, unique assemblies were created for each strain, first by clustering the oases outputs using usearch (version 5.2.32; (Edgar 2010)), and later carrying out a new assembly with the non-redundant outputs with CAP3 (version date 12/21/2007; (Huang and Madan 1999)). For *de novo* assembly of sample #10 (Table S1), nucleotides with low quality (less than 20) were masked by N and reads with more than 50 contiguous non-N nucleotide were selected. The selected RNA-seq dataset was assembled *de novo* using Trinity software released at 2013_08_14 (Grabherr, et al. 2011). The *de novo* assembly of the sample #11 RNA-seq dataset from the 454 run (Table S1) was performed by using GS De Novo Assembler (Rhoche). All RNA-seq outputs were deposited in the European Nucleotide Archive (ENA; (Leinonen, et al. 2011)), under the study accession numbers SRP009381 and ERP006235, except for the previously reported WT31 sequencing (Barrantes, et al. 2012) or DDBJ Sequence Read Archive (DRA), under the accession number DRA004071. A summary of sequencing and assembly results is given in Table S1.

3. Intron Size Distribution and Domains

3.1 Intron sizes

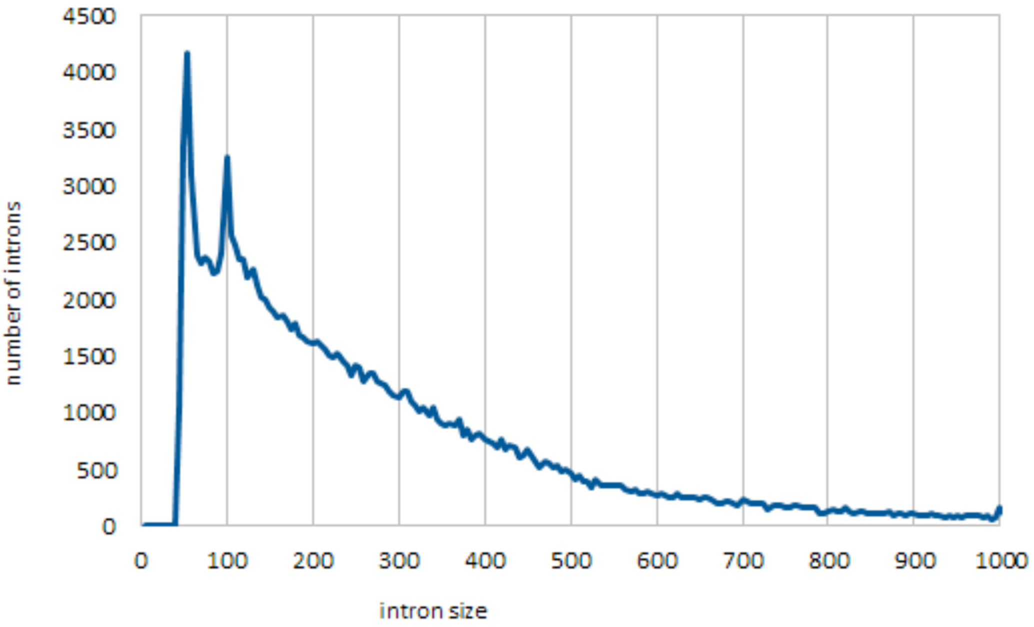

Figure S2. Intron sizes in *P. polycephalum*. Only introns supported by transcript data are shown.

3.2 Domains

Domains were determined using IPRscan (Zdobnov and Apweiler 2001) with default settings. Several pfam entries have overlapping functions so the lack of a domain does not mean necessarily lack of function. Domains not found in *D. discoideum* but in *P. polycephalum* and *A. castellanii* are given in Table S2 whereas domains overrepresented in or restricted to *P. polycephalum* as compared to *D. discoideum* and *A. castellanii* are listed in Table S3.

Table S2. Domains not found in *D. discoideum* but in *P. polycephalum* (PP) and *A. castellanii* (AC). Functions given are broad categories.

pfam ID	description	*PP*	*AC*	function
PF01436	NHL repeat	15	12	signalling
PF01590	GAF domain	14	10	signalling
PF02750	Synapsin, ATP binding domain	11	9	vesicle transport
PF02450	Lecithin:cholesterol acyltransferase	10	19	enzyme
PF01534	Frizzled/Smoothened family membrane region	8	16	signalling
PF07173	Protein of unknown function (DUF1399)	8	9	ND
PF12849	PBP superfamily domain	8	101	ND
PF02836	Glycosyl hydrolases family 2, TIM barrel do-main	7	4	carbohydrate metabo-lism
PF05050	Methyltransferase FkbM domain	7	5	enzyme
PF12854	PPR repeat	7	9	repeats
PF00079	Serpin (serine protease inhibitor)	6	7	protein bin-ding/degradation
PF00975	Thioesterase domain	6	4	enzyme
PF02140	Galactose binding lectin domain	6	11	carbohydrate metabo-lism
PF13360	PQQ-like domain	6	7	repeats
PF00641	Zn-finger in Ran binding protein and others	5	18	signalling
PF00701	Dihydrodipicolinate synthetase family	5	3	enzyme
PF02182	SAD/SRA domain	5	5	Nucleotides
PF02668	Taurine catabolism dioxygenase TauD, TfdA family	5	3	enzyme
PF06101	Plant protein of unknown function (DUF946)	5	5	ND
PF07676	WD40-like Beta Propeller Repeat	5	4	signalling
PF07995	Glucose / Sorbosone dehydrogenase	5	4	enzyme
PF13458	Periplasmic binding protein	5	10	bacteria signalling
PF13594	Amidohydrolase	5	7	enzyme
PF13598	Domain of unknown function (DUF4139)	5	4	ND
PF01483	Proprotein convertase P-domain	4	3	enzyme

Table S3. Domains overrepresented in or restricted to *P. polycephalum* (PP). Overrepresentation was defined as *P. polycephalum* having at least 5 different proteins with a given domain and the other species at most half of those (*D. discoideum*: DD; *A. castellanii*: AC). Functions given are broad categories.

pfam ID	description	*PP*	*DD*	*AC*	function
PF07727	Reverse transcriptase (RNA-dependent DNA polymerase)	827	0	20	transposon
PF00665	Integrase core domain	484	24	7	transposon
PF01068	ATP dependent DNA ligase domain	285	3	6	Nucleotides
PF13976	GAG-pre-integrase domain	171	0	0	transposon
PF00098	Zinc knuckle	101	8	16	transposon
PF13229	Right handed beta helix region	76	2	12	degradation
PF01535	PPR repeat	72	5	24	repeats
PF07691	PA14 domain	62	22	0	spore formation
PF13812	Pentatricopeptide repeat domain	58	2	29	repeats
PF13041	PPR repeat family	55	4	20	repeats
PF14227	gag-polypeptide of LTR copia-type	43	0	0	transposon
PF00225	Kinesin motor domain	43	13	20	intracellular move-ment
PF00550	Phosphopantetheine attachment site	43	12	7	enzyme
PF07645	Calcium-binding EGF domain	43	5	1	membrane
PF13573	SprB repeat	41	0	0	cell surface
PF00722	Glycosyl hydrolases family 16	41	4	6	enzyme
PF00385	Chromo (CHRromatin Organisation MOdifier) domain	39	13	19	signalling
PF13519	von Willebrand factor type A domain	39	11	16	signalling
PF00615	Regulator of G protein signaling domain	39	10	9	signalling
PF00027	Cyclic nucleotide-binding domain	30	5	4	enzyme
PF00041	Fibronectin type III domain	30	0	8	membrane
PF12947	EGF domain	28	0	1	membrane
PF01094	Receptor family ligand binding region	27	1	0	signalling
PF01822	WSC domain	26	1	0	carbohydrate metabo-lism
PF00702	haloacid dehalogenase-like hydrolase	26	12	12	degradation
PF07534	TLD	26	6	13	enzyme
PF08477	Miro-like protein	24	8	12	signalling
PF00075	RNase H	24	2	4	Nucleotides
PF13676	TIR domain	23	1	0	signalling
PF03028	Dynein heavy chain and region D6 of dynein motor	23	1	2	transport
PF13460	NADH(P)-binding	23	5	7	enzyme

PF00534	Glycosyl transferases group 1	22	8	7	carbohydrate metabo-lism
PF00732	GMC oxidoreductase	22	2	6	enzyme
PF07748	Glycosyl hydrolases family 38 C-terminal domain	22	3	6	enzyme
PF12708	Pectate lyase superfamily protein	21	0	0	cell surface
PF00172	Fungal Zn(2)-Cys(6) binuclear cluster domain	21	2	8	transcription
PF06367	Diaphanous FH3 Domain	20	6	9	cytoskeleton
PF13650	Aspartyl protease	20	9	0	protein bin-ding/degradation
PF14223	gag-polypeptide of LTR copia-type	20	0	2	transposon
PF06371	Diaphanous GTPase-binding Domain	19	8	7	cytoskeleton
PF00092	von Willebrand factor type A domain	19	4	6	signalling
PF03732	Retrotransposon gag protein	19	1	1	transposon
PF13847	Methyltransferase domain	18	8	9	enzyme
PF00685	Sulfotransferase domain	18	2	3	enzyme
PF00728	Glycosyl hydrolase family 20, catalytic domain	17	5	4	enzyme
PF00520	Ion transport protein	16	4	7	transport
PF00690	Cation transporter/ATPase, N-terminus	16	7	7	transport
PF01074	Glycosyl hydrolases family 38 N-terminal domain	16	7	6	carbohydrate metabo-lism
PF03283	Pectinacetylesterase	16	0	3	degradation
PF05183	RNA dependent RNA polymerase	16	3	3	Nucleotides
PF05729	NACHT domain	16	3	3	Nucleotides
PF02415	Chlamydia polymorphic membrane protein (Chlamydia_PMP) repeat	15	1	0	cell surface
PF08393	Dynein heavy chain, N-terminal region 2	15	1	0	intracellular move-ment
PF09337	His(2)-Cys(2) zinc finger	15	0	0	transcription
PF02816	Alpha-kinase family	15	6	2	kinase
PF03171	2OG-Fe(II) oxygenase superfamily	15	6	7	enzyme
PF00348	Polyprenyl synthetase	15	5	3	enzyme
PF01762	Galactosyltransferase	14	0	0	carbohydrate metabo-lism
PF00026	Eukaryotic aspartyl protease	14	5	7	protein bin-ding/degradation
PF00200	Disintegrin	13	0	0	signalling
PF13255	Protein of unknown function (DUF4046)	13	0	0	no
PF00689	Cation transporting ATPase, C-terminus	13	6	5	transport
PF00704	Glycosyl hydrolases family 18	13	6	4	carbohydrate metabo-lism
PF02771	Acyl-CoA dehydrogenase, N-terminal domain	13	5	6	enzyme
PF12775	P-loop containing dynein motor region D3	13	1	1	transport

PF12780	P-loop containing dynein motor region D4	12	1	0	intracellular movement
PF07728	AAA domain (dynein-related subfamily)	12	2	6	intracellular movement
PF09379	FERM N-terminal domain	12	3	5	
PF00668	Condensation domain	12	0	3	PKS
PF12777	Microtubule-binding stalk of dynein motor	12	1	1	transport
PF12781	ATP-binding dynein motor region D5	12	1	2	transport
PF13246	Putative hydrolase of sodium-potassium ATPase alpha subunit	12	3	4	enzyme
PF01823	MAC/Perforin domain	11	1	0	immunity
PF02263	Guanylate-binding protein, N-terminal domain	11	2	0	immunity
PF12774	Hydrolytic ATP binding site of dynein motor region D1	11	1	0	intracellular movement
PF00002	7 transmembrane receptor (Secretin family)	11	5	4	signalling
PF05199	GMC oxidoreductase	11	2	5	enzyme
PF14033	Protein of unknown function (DUF4246)	11	0	3	ND
PF08284	Retroviral aspartyl protease	10	0	0	transposon
PF08757	CotH protein	10	0	0	cell surface
PF00285	Citrate synthase	10	5	4	enzyme
PF00648	Calpain family cysteine protease	10	0	5	enzyme
PF02171	Piwi domain	10	5	3	Nucleotides
PF00884	Sulfatase	10	0	1	enzyme
PF01184	GPR1/FUN34/yaaH family	10	0	3	transport
PF07699	GCC2 and GCC3	10	0	1	signalling
PF14432	DYW family of nucleic acid deaminases	10	0	1	Nucleotides
PF02156	Glycosyl hydrolase family 26	9	0	0	carbohydrate metabolism
PF05426	Alginate lyase	9	0	0	carbohydrate metabolism
PF09362	Domain of unknown function (DUF1996)	9	0	0	no
PF00339	Arrestin (or S-antigen), N-terminal domain	9	4	1	signalling
PF11790	Glycosyl hydrolase catalytic core	9	1	4	carbohydrate metabolism
PF00191	Annexin	9	3	0	ND
PF00233	3'5'-cyclic nucleotide phosphodiesterase	9	3	2	Nucleotides
PF00614	Phospholipase D Active site motif	9	3	3	enzyme
PF01391	Collagen triple helix repeat (20 copies)	9	2	0	ND
PF05224	NDT80 / PhoG like DNA-binding family	9	2	0	Nucleotides
PF08447	PAS fold	9	3	3	signalling
PF00623	RNA polymerase Rpb1, domain 2	8	3	3	Nucleotides
PF00855	PWWP domain	8	4	2	signalling

PF01544	CorA-like Mg2+ transporter protein	8	3	2	transport
PF02225	PA domain	8	0	4	degradation
PF02738	Molybdopterin-binding domain of aldehyde dehydrogenase	8	1	4	enzyme
PF02837	Glycosyl hydrolases family 2, sugar binding domain	8	0	4	enzyme
PF04185	Phosphoesterase family	8	0	4	enzyme
PF12874	Zinc-finger of C2H2 type	8	4	4	zinc finger
PF13271	Domain of unknown function (DUF4062)	8	4	4	ND
PF03060	Nitronate monooxygenase	8	1	2	enzyme
PF00324	Amino acid permease	7	1	0	transport
PF00001	7 transmembrane receptor (rhodopsin family)	7	0	0	signalling
PF01150	GDA1/CD39 (nucleoside phosphatase) family	7	0	0	no
PF01697	Glycosyltransferase family 92	7	0	0	carbohydrate metabolism
PF02057	Glycosyl hydrolase family 59	7	0	0	carbohydrate metabolism
PF13313	Domain of unknown function (DUF4082)	7	0	0	no
PF00199	Catalase	7	2	3	enzyme
PF01453	D-mannose binding lectin	7	3	0	ND
PF01699	Sodium/calcium exchanger protein	7	3	3	transport
PF03016	Exostosin family	7	0	3	enzyme
PF04983	RNA polymerase Rpb1, domain 3	7	3	3	Nucleotides
PF00232	Glycosyl hydrolase family 1	7	0	1	enzyme
PF00703	Glycosyl hydrolases family 2	7	1	2	enzyme
PF02275	Linear amide C-N hydrolases, choloylglycine hydrolase family	7	0	2	enzyme
PF03629	Domain of unknown function (DUF303)	7	0	2	ND
PF05592	Bacterial alpha-L-rhamnosidase	7	0	2	Bacterial enzyme
PF08385	Dynein heavy chain, N-terminal region 1	7	1	1	transport
PF10443	RNA12 protein	7	2	1	Nucleotides
PF00840	Glycosyl hydrolase family 7	6	1	0	carbohydrate metabolism
PF01082	Copper type II ascorbate-dependent monooxygenase, N-terminal domain	6	0	0	no
PF05791	Bacillus haemolytic enterotoxin (HBL)	6	0	0	immunity
PF07940	Heparinase II/III-like protein	6	0	0	immunity
PF01042	Endoribonuclease L-PSP	6	3	3	Nucleotides
PF01798	Putative snoRNA binding domain	6	3	3	Nucleotides
PF08060	NOSIC (NUC001) domain	6	3	3	ND
PF08953	Domain of unknown function (DUF1899)	6	3	3	ND
PF13207	AAA domain	6	3	3	enzyme

PF14295	PAN domain	6	1	3	ND
PF00190	Cupin	6	0	1	enzyme
PF01031	Dynamin central region	6	2	1	endocytosis
PF01229	Glycosyl hydrolases family 39	6	1	1	enzyme
PF01541	GIY-YIG catalytic domain	6	0	1	Nucleotides
PF02010	REJ domain	6	1	2	ND
PF02898	Nitric oxide synthase, oxygenase domain	6	0	1	enzyme
PF02900	Catalytic LigB subunit of aromatic ring-opening dioxygenase	6	1	2	enzyme
PF03659	Glycosyl hydrolase family 71	6	0	1	enzyme
PF08016	Polycystin cation channel	6	2	1	transport
PF10021	Uncharacterized protein conserved in bacteria (DUF2263)	6	0	2	ND
PF10551	MULE transposase domain	6	0	1	transposon
PF00295	Glycosyl hydrolases family 28	5	0	0	carbohydrate metabolism
PF01359	Transposase (partial DDE domain)	5	0	0	transposon
PF02055	O-Glycosyl hydrolase family 30	5	0	0	carbohydrate metabolism
PF02074	Carboxypeptidase Taq (M32) metallopeptidase	5	0	0	protein binding/degradation
PF02128	Fungalysin metallopeptidase (M36)	5	0	0	protein binding/degradation
PF03160	Calx-beta domain	5	0	0	transport
PF03639	Glycosyl hydrolase family 81	5	0	0	carbohydrate metabolism
PF03866	Hydrophobic abundant protein (HAP)	5	0	0	cell surface
PF06990	Galactose-3-O-sulfotransferase	5	0	0	carbohydrate metabolism
PF07143	Hydroxyneurosporene synthase (CrtC)	5	0	0	no
PF07944	Putative glycosyl hydrolase of unknown function (DUF1680)	5	0	0	carbohydrate metabolism
PF11369	Protein of unknown function (DUF3160)	5	0	0	no
PF12138	Spherulation-specific family 4	5	0	0	spore formation
PF00205	Thiamine pyrophosphate enzyme, central domain	5	2	2	enzyme
PF01738	Dienelactone hydrolase family	5	1	2	enzyme
PF02698	DUF218 domain	5	1	2	ND
PF02729	Aspartate/ornithine carbamoyltransferase, carbamoyl-P binding domain	5	1	2	enzyme
PF04030	D-arabinono-1,4-lactone oxidase	5	0	2	enzyme
PF04261	Dyp-type peroxidase family	5	2	2	enzyme
PF05903	PPPDE putative peptidase domain	5	2	2	degradation
PF07522	DNA repair metallo-beta-lactamase	5	1	2	Nucleotides

PF08156	NOP5NT (NUC127) domain	5	2	2	Nucleotides
PF11894	Protein of unknown function (DUF3414)	5	2	1	ND
PF12348	CLASP N terminal	5	2	2	transport
PF13401	AAA domain	5	2	0	enzyme
PF01496	V-type ATPase 116kDa subunit family	5	1	1	enzyme
PF01555	DNA methylase	5	0	1	Nucleotides
PF04616	Glycosyl hydrolases family 43	5	0	1	enzyme
PF05786	Condensin complex subunit 2	5	1	1	Mitosis
PF12831	FAD dependent oxidoreductase	5	0	1	enzyme
PF13304	AAA domain	5	0	1	enzyme
PF14624	VWA / Hh protein intein-like	5	0	1	ND

4. Transposable Elements

4.1 DNA transposons

The *P. polycephalum* genome appears to be essentially free of DNA transposons. Only one incomplete copy of a DNA transposon containing a Tc1/mariner-related transposase was detected.

4.2 LTR retrotransposons

The *P. polycephalum* genome was searched for reverse transcriptase domains related to *D. discoideum* LTR retrotransposons DGLT-A and Skipper using tblastn. The *P. polycephalum* genome contains a family of 10 related LTR retrotransposons with recognizable ORFs (Fig. S3) and many fragments that hampered the determination of exact copy numbers. The total genome content of LTR retrotransposons is estimated at 0.51%. Five elements have intact open reading frames (ORFs) and may be retrotransposition-competent. The *P. polycephalum* elements are related to the Ty3/Gypsy group of retrotransposons and share 40-45% amino acid sequence identity with *Saccharomyces cerevisiae* Ty3 in the concatenated reverse transcriptase (RT), ribonuclease H (RNH), and integrase (IN) core domains. Phylogenetic analysis suggests that the *P. polycephalum* LTR retroransposons had a common ancestor with the *D. discoideum* DGLT-A and Skipper elements, with which they share betweeen 30 and 41% sequence identity (Fig. S4). The *P. polycephalum* LTR elements do not display any integration preference for tRNA genes as observed for the *D. discoideum* DGLT-A element.

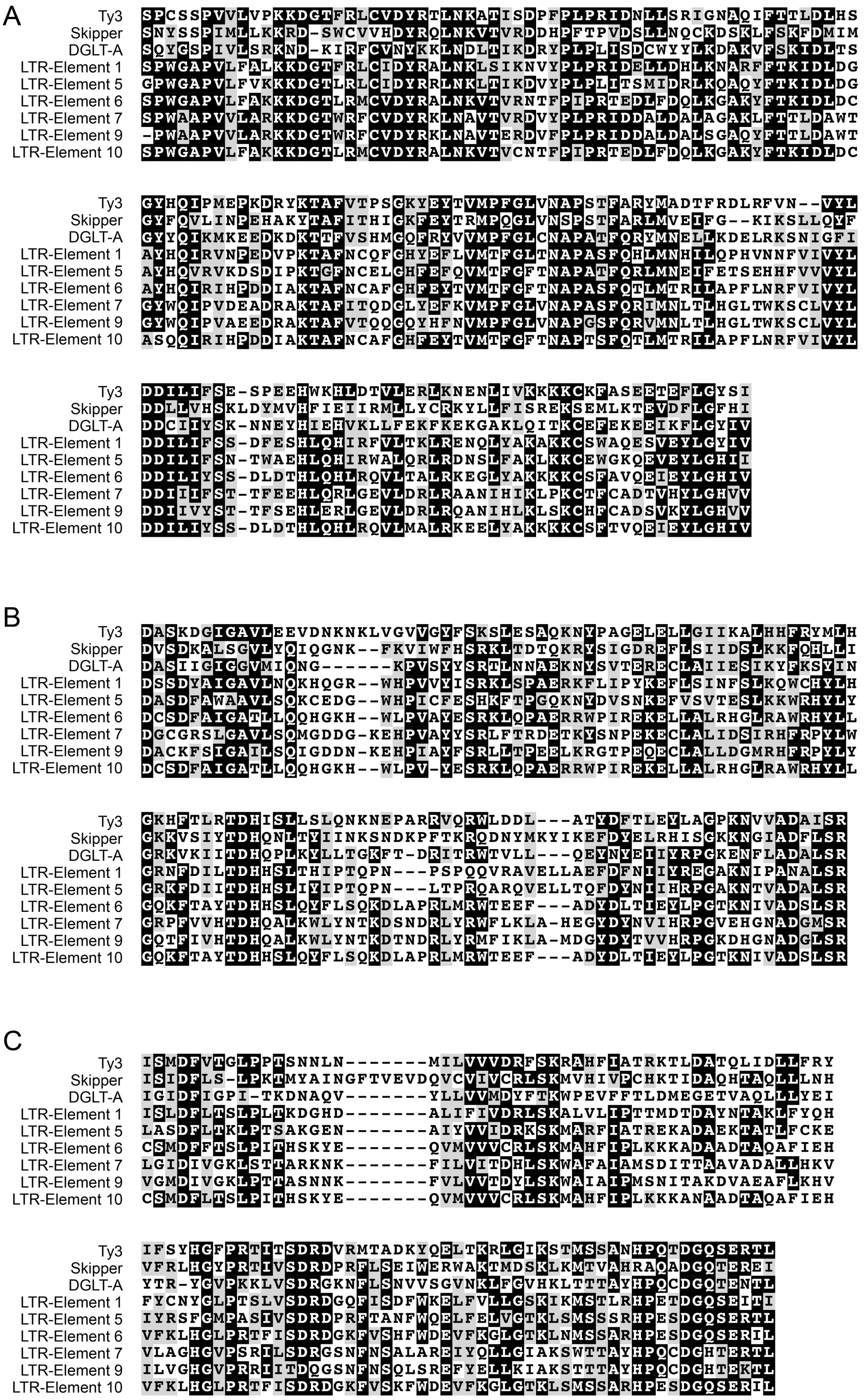

Figure S3. Alignments of RT, RNH, and IN domains of *P. polycephalum* LTR retrotransposons. Core domains of reverse transcriptase (RT), ribonuclease H (RNH), and integrase (IN) were determined by searching the Conserved Domain Database (Marchler-Bauer, et al. 2015). *P. polycephalum* LTR retrotransposons 1-6 are compared with *D. discoideum* DGLT-A and Skipper (Glöckner, et al. 2001) and *S. cerevisiae* Ty3 (M23367). Alignments were generated with CLUSTALX (Thompson, et al. 1997) and BOXSHADE (http://www.ch.embnet.org/software/BOX_form.html). **(A)**: RT domain; **(B)**: RNH domain; **(C)**: IN domain.

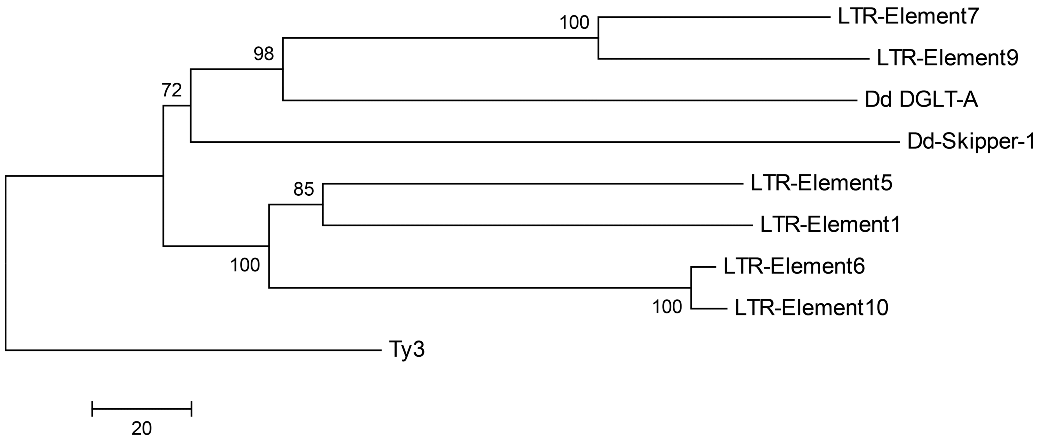

Figure S4. Phylogenetic analysis of *P. polycephalum* LTR retrotransposons.The evolutionary history was analyzed using the Neighbor-Joining method (Saitou and Nei 1987). The optimal tree with the sum of branch length = 719.3125 is shown. The percentage of replicate trees in which the associated taxa clustered together in the bootstrap test (100 replicates) are shown next to the branches (Felsenstein 1985). The tree is drawn to scale, with branch lengths in the same units as those of the evolutionary distances used to infer the phylogenetic tree. The evolutionary distances were computed using the number of differences method (Nei and Kumar 2000) and are in the units of the number of amino acid differences per sequence. All positions with less than 100% site coverage were eliminated. A total of 385 positions in the final dataset represented the concatenated RT, RNH, and IN core domains. Evolutionary analyses were conducted in MEGA5 (Tamura, et al. 2007).

4.3 Non-LTR retrotransposons

The *P. polycephalum* genome was searched for reverse transriptase domains related to *D. discoideum* non-LTR retroransposons TRE5-A and TRE3-A using tblastn. Appoximately 0.8% of the *P. polycephalum* genome are non-LTR retrotransposons. All these elements are highly degenerated and no complete structures could be assembled. Some of these elements seem to contain RNH domains downstream of the RT, but the elements could not be reliably assigned to a particular familiy of non-LTR retrotransposons as defined by Malik and Eickbush (Malik and Eickbush 1999, 2001). In *D. discoideum*, all non-LTR retrotransposons form a monophyletic family that displays a strong integration preference either upstream or downstream of tRNA genes (TRE5 and TRE3 elements, respectively). The *D. discoideum* TRE elements belong to the L1 class of non-LTR retrotransposons (Malik, et al. 1999). Phylogenetic analysis of *P. polycephalum* non-LTR retrotransposons suggests that they form a separate clade that is rather distantly related to *D. discoideum* TRE elements (Fig. S5). No signs of tRNA gene-targeted integration of *P. polycephalum* non-LTR retrotransposons were observed. For a quantitative overview on transposable elements in *P. polycephalum* see Table S4.

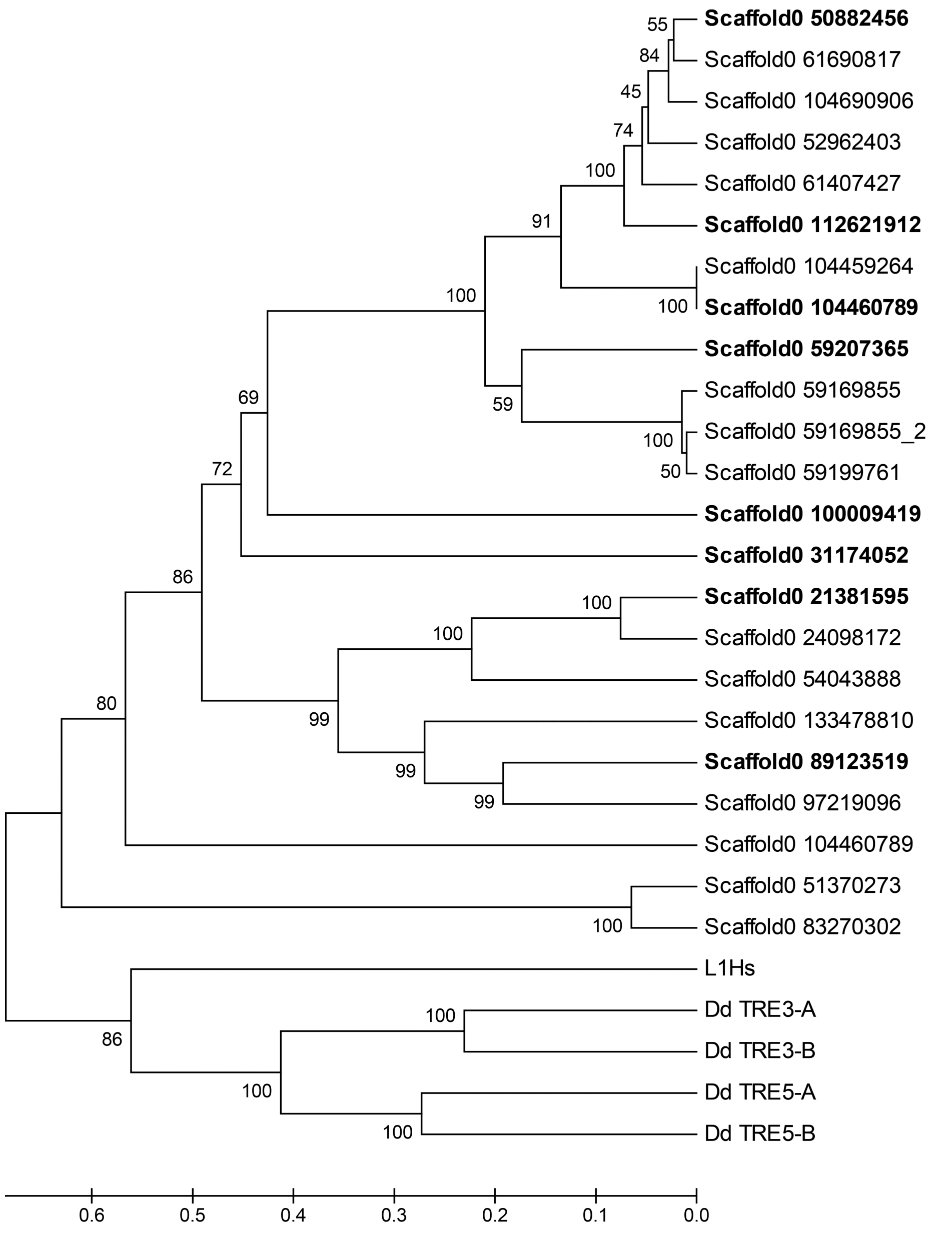

Figure S5. Phylogenetic analysis of *P. polycephalum* non-LTR retrotransposons. The evolutionary history was inferred using the UPGMA method (Sneath and Sokal 1973). The optimal tree with the sum of branch length = 7.3580 is shown. The percentage of replicate trees in which the associated taxa clustered together in the bootstrap test (100 replicates) are shown next to the branches (Felsenstein 1985). The tree is drawn to scale, with branch lengths in the same units as those of the evolutionary distances used to infer the phylogenetic tree. The evolutionary distances were computed using the Poisson correction method (Zuckerkandl and Pauling 1965) and are in the units of the number of amino acid substitutions per site. All positions with less than 100% site coverage were eliminated. There were a total of 157 positions in the final dataset. Evolutionary analyses were conducted in MEGA5 (Tamura, et al. 2007). Elements highlighted in bold contain RNH domains.

Table S4. Transposable elements comprise approximately 1.3% of the *P. polycephalum* genome.

	sequence (bp)	max. E-value	genome content (%)
LTR retrotransposons	1,074,799	1,00E-30	0.513
Non-LTR-retrotransosons	1,677,256	1,00E-30	0.801
DNA transposons	2,779	1,00E-10	0.001
∑	2,754,834		1.315

5. Small non-coding RNAs

We used the mitochondrial small RNA sequencing data from ((Bundschuh, et al. 2011), SRA accession SRX03323) to annotate small RNAs in the nuclear *P. polycephalum* genome. We first mapped its reads to the mitochondrial *P. polycephalum* genome (Takano, et al. 2001) using a custom mapper that allows reads to be mapped even in the presence of the prevalent RNA editing known to exist in the *P. polycephalum* mitochondrion (Bundschuh, et al. 2011). We then map all reads that did not map to the mitochondrial genome to the nuclear genomic contigs using BLAST (Altschul, et al. 1997) at an E-value threshold of 0.0001. We count the reads mapping to every contig and only kept contigs for further processing if at least 100 reads were mapped to them.

For each of these contigs we calculated the coverage by our (stranded) sequencing reads in both orientations and chose the orientation with the higher total coverage. We then separated the contig into individual putative small RNA sequences by identifying the point of highest coverage in the contig and requiring this coverage maximum to be at least 50, extending the putative small RNA in both directions until the end of the contig was reached or unless the coverage at least 5 consecutive positions was below 1/1000 of the coverage at the initial maximum, and repeating the process for each of the remaining parts of the contig until no more putative small RNAs were found. We discarded any putative small RNAs shorter than 15 nucleotides.

We annotated the resulting putative small RNAs using BLAST against the non-redundant (nr) nucleotide database. We manually curated a list of accession numbers of known *Physarum* RNAs and annotated a putative small RNA as known if its top BLAST hit was one of those accession numbers, has an E-value less than 0.001, and has a number of mismatches of at most 0 for tRNAs, 2-5 for other short RNAs, and 15 for ribosomal RNAs (Supplemental Spreadsheet 1, 1st sheet). We also manually identified several of the putative small RNAs as ribosomal based on their BLAST hits even though their top hits are not from *Physarum* and discard them.

All remaining putative small RNAs were classified as novel small RNAs (Supplemental Spreadsheet 1, 2nd sheet). Some of them had multiple highly significant BLAST hits in the

non-redundant database and could be identified as tRNAs or spliceosomal RNAs. In the case of tRNAs, the sequences were manually trimmed and/or extended and fitted into the standard tRNA sequence/structure template. None of the remaining RNAs had a similarity with E-value below 0.001 in the non-redundant database and were classified as unknown RNAs. If they contained the RUGAUGA/CUGA motif, they were classified as putative box c/d snoRNAs and if they were longer than 200 nucleotides, they were classified as unknown lncRNAs. Unknown RNAs were classified into groups if a BLAST search of one against the other using the size of the nr database to calculate E-values resulted in a significant (E<0.001) similarity. All unknown RNAs were compared using tblastx to the non-redundant protein database to identify possible protein coding sequences but none yielded a significant similarity.

6. MicroRNA and Associated mRNA Targets

6.1 Overview

It has been well established that in response to external stress stimuli, cellular modifications involve transcriptional, translational, post-translational, allosteric and microRNA (miRNA) regulation. MiRNA are short (18–23 nt), non-coding RNAs that are known to have central roles in regulating the post-transcriptional expression of mRNA transcripts. To date, more than 2500 human mature miRNA species have been predicted, many of which are conserved throughout evolution. Currently, there are only 17 precursors and 20 mature miRNA currently deposited in miRBase (v.21) for all Mycetozoa. Given the wide-spread importance of miRNA in the post-translational regulation of mRNA transcripts across the animal kingdom, the identification of miRNA in *P. polycephalum* is critical to fully understand its cellular function and its regulatory mechanisms. Our study utilizes a newly developed species-specific prediction pipeline to predict miRNA sequences in the *P. polycephalum* genome. Identification of these miRNAs may provide information on the conservation of miRNAs and their on influence translational regulation in this species.

To establish a repertoire of small regulatory RNAs within the *P. polycephalum* genome, we performed an *in silico* prediction of miRNAs within the genome scaffolds. Uniquely, we leverage a novel approach to miRNA classifier construction in which models are built dynamically and are targeted toward the species that are being studied. In order to generate a targeted model for a species, our precursor miRNA prediction approach generates data sets of real- and pseudo- miRNA from species that are closely related to the targeted species. From this approach, we were able to successfully identify 48 precursor (2 conserved and 46 novel) and associated mature miRNA from *P. polycephalum* (Supplemental Spreadsheet 3). Notably, the sequence density of Mycetozoa mature miRNA (including those found in this study) were most similar to that of Viridiplantae (i.e., plants), when compared with Metazoa and Virus groups from miRBase (v.21) (Fig. S6). Additionally, of the 17 currently annotated precursor miRNAs for *Dictyostelium discoideum* (Avesson, et al. 2012), no homology was found with any miRNA from the annotated *P. polycephalum* genome.

To identify possible biological contexts for annotated *P. polycephalum* miRNA, mRNA targets were predicted using miRanda (v.3.3a) (Supplemental Spreadsheet 4). A total of 4541 mRNA transcripts were predicted to be targets of the identified mature miRNAs. For example, a novel *P. polycephalum* miRNA (novel-ppo-miR-8) was predicted to target the Dynein heavy chain 1 (cytoplasmic) – like mRNA (Fig. S7). It is possible that such *P. polycephalum*-specific miRNAs may be involved in cellular pathways that contribute to slime

mold biology (such as cell motility). The presented newly identified miRNAs greatly enrich the repertoire of Mycetozoa miRNA, as well as future insight into evolution and biogenesis.

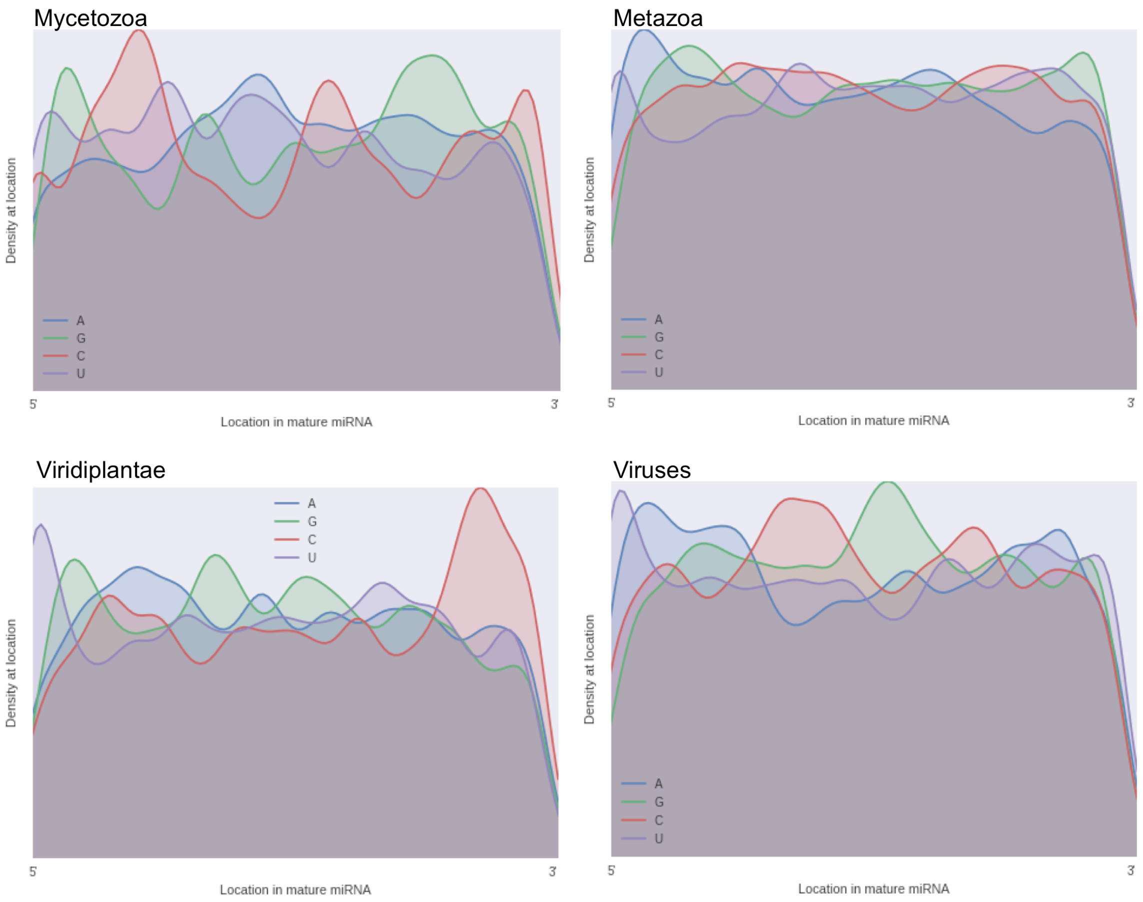

Figure S6. Depiction of mature miRNA sequence density. The abundance of nucleotide type (GCAU) are compared along the length of mature miRNA from Mycetozoa (including miRNA identified in this study), Metazoa, Viridiplantae, and Viruses deposited within the miRBase (v.21) database.

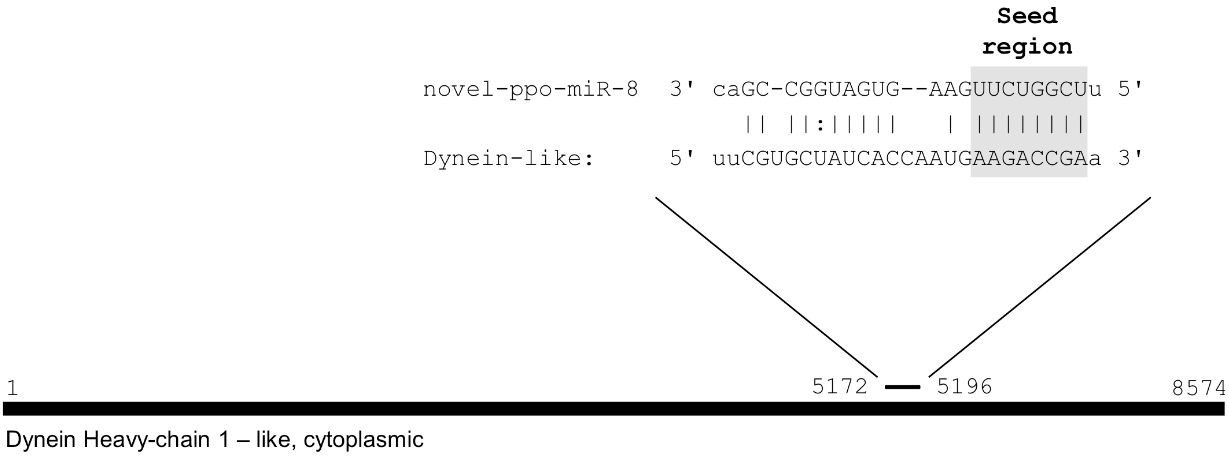

Figure S7. Identification of a novel *P. polycephalum* mature miRNA (novel-ppo-miR-8) predicted to bind Dynein Heavy Chain 1 (cytoplasmic) - like mRNA. The critical binding of the seed region between the bound miRNA and mRNA is shaded.

6.2 Materials & Methods

Species-specific precursor microRNA prediction. Novel precursor microRNA sequences were predicted from *P. polycephalum* using SMIRP, a species-specific microRNA prediction program that uses miRNA classifier construction in which prediction models are built dynamically and are targeted toward the species that are being studied (http://bioinf.sce.carleton.ca/SMIRP). The HeterMirPred feature set was used for classification of *P. polycephalum* miRNAs using our targeted positive and negative training sets (defined below), and consists of 20 distinct sequence and structural features. We have chosen to use a support vector machine (SVM) as our classifier as opposed to the ensemble classifier, due to the ability of a SVM to provide clear probability estimates along with its determinations of real or pseudo-microRNA (Chih-Chung and Chih-Jen 2011). Our positive training dataset for *P. polycephalum* miRNA classification was generated using a targeted sequence dataset from miRBase (v.21). Negative training datasets are generated from the reference transcriptome of *P. polycephalum* (http://www.physarum-blastovgu.de/transcriptome.html) and the *D. discoideum* coding region sequence data (http://dictybase.org/db/). From coding regions, sequences that resemble miRNA (Hofactor and Fontana 1994) were extracted as used to generate the negative training dataset.

Validation of novel precursor microRNA prediction and mature microRNA prediction. To help validate predicted precursor miRNA above the 0.95 confidence cut-off value, we used the available reference transcriptome data for *P. polycephalum*. We used Blast+ (v.2.2.28) to remove candidate precursor miRNA that were present within the poly-adenylate enriched reference transcriptome database for *P. polycephalum*. Additional validation was carried out on predicted precursor miRNA by ensuring that each sequence was capable of producing mature miRNA using MiRdup (v.1.2), with the programs default settings (Leclercq, et al. 2013).

Target prediction. Mature miRNAs were searched against the *P. polycephalum* reference transcriptome using miRanda (v.3.3a) (Enright, et al. 2003). To search the predicted miRNA target genes within *P. polycephalum* reference transcriptome the miRanda software was used within the following parameters and conditions: a gap opening penalty of − 9, a gap extension penalty of − 4; match with minimum score threshold 160, target duplex with maximum threshold free energy – 25 kCal/mol, scaling parameter 4 for complementary nucleotide match score, counting from the miRNA 5′ end, and demand strict 5′ seed pairing on between 2 and 9 nucleotides (Supplemental Spreadsheet 5).

7. Pteridine Metabolism

7.1 Background

The group of pteridines comprises a rich variety of low molecular weight coenzymes essential for metabolism that all are synthesized from the common precursor guanosine 5' triphosphate (GTP, Fig. S8). Pteridines contain as a common structural element the pteridine (pyrimido[4,5-b]pyrazine) bicyclic ring system (red in Fig. S8). Pteridine cofactors comprise molybdopterin, tetrahydrofolate, tetrahydrobiopterin, riboflavin. Biosynthesis of the modified nucleotide 7-aminomethyl-7-deazaguanine (preQ1) which proceeds via the central pteridine metabolite 7,8-dihydroneopterin triphosphate is also part of the pteridine metabolism.

Molybdopterin serves as cofactor for e.g. xanthine and sulfite oxidases (Schwarz, et al. 2009). Tetrahydrofolate is essential for one carbon metabolism e.g. biosynthesis of the pyrimidine base thymine required for DNA synthesis (Stover 2011). Tetrahydrobiopterin is an essential cofactor for monooxygenases such as phenylalanine hydroxylase and for nitric oxide synthases (Werner, et al. 2011). 7-Aminomethyl-7-deazaguanine (preQ1) is further converted to queuosine (Q) and incorporated as a special base into t-RNAs in most living organisms including humans, with the exception of e.g. archaea and yeast (Slany and Kersten 1994). Riboflavin is essential for all organisms as precursor of the flavin containing nucleotides FAD and FMN. Animals and humans have to take up riboflavin, tetrahydrofolate and queuosine or its precursors from the diet (Rakovich, et al. 2011). Tetrahydrobiopterin, in contrast, is formed mainly in animals and not found in plants, nor in most fungi or bacteria, which also do not need this cofactor for their metabolism.

7.2 Methods

Protein sequences for the respective pathways were taken from the KEGG metabolic schemes using sequences from *Homo sapiens*, *Saccharomyces cerevisiae*, *Dictyostelium discoideum* and *Arabidopsis thaliana*. Genomic and transcriptomic databases were searched with the tblastn program at the *P. polycephalum* genome server. All genes listed in Table S5 occur not only in the genomic sequence but also in one or more of the transcriptomic databases. Annotation of the reading frames found were confirmed using the conserved domain database of the National Institute of Biotechnology Information (NCBI). Deduced protein sequences were compared to the non-redundant protein database (nr) of NCBI using the blastp program for the search for closest orthologs.

7.3 Results

We compared the genome and transcriptomes of *P. polycephalum* for the occurrence of open reading frames for the pteridine pathways shown in Fig. S8. As reference organisms, we chose humans (H, *homo sapiens*), a yeast (Y, *Saccaromyces cerevisiae*), a plant (A, *Arabidopsis thaliana*), a bacterium (E, *Escherichia coli*), and the two molds P, *P. polycephalum* and D, *Dictyostelium discoideum.* We did not include an archaeon since current research likely is about to change our view on pterin metabolism in archaea due to the occurrence of novel GTP cyclohydrolases termed type 3 and 4 (Morrison, et al. 2008). Among the selected representative species, surprisingly, *P. polycephalum* is the only one containing all reading frames for

all the pathways (I) through (VI) shown in Fig. S8, (Table S5), which will be briefly detailed in the following paragraphs.

(I) Molydbopterin synthesis is conserved among all species investigated, and *P. polycephalum* contains one reading frame for all enzymatic steps required. As in humans (Stallmeyer, et al. 1999), the small and the large subunits of molybdopterin synthase (*i.e.* MOCS2A and MOCS2B) are encoded by two reading frames on the same messenger RNA. Interestingly, *Dictyostelium discoideum* expresses these two reading frames (MOCS2s and MOCS2l) separately from two different chromosomes (Fig. S9)

(II) GTP cyclohydrolase 1 is also conserved among the species shown in Fig. S8. Among these *P. polycephalum* is the only species to contain reading frames for two isoforms of this enzyme with related amino acid sequences, both of which are expressed, and, as judged from conservation of sequence motifs, are likely to be functional. A similar situation was found for calcium-independent nitric oxide synthases (NOS2a and NOS2b) *in P. polycephalum* (see below) which both are expressed and are enzymatically functional (Messner, et al. 2009).

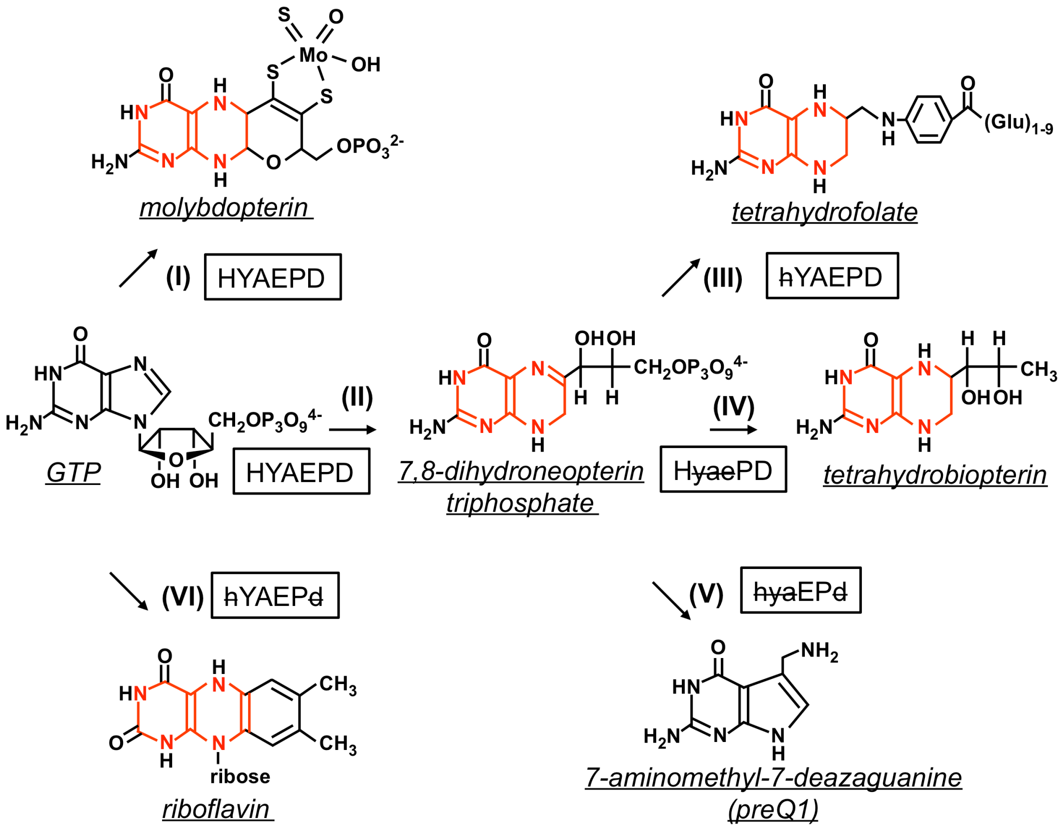

Figure S8. Pathways from GTP to pteridine compounds. (I), molybopterin synthesis; (II), GTP cyclohydrolase 1; (III), tetrahydrofolate synthesis; (IV), tetrahydrobiopterin synthesis (V) 7-aminomethyl-7-deazaguanin synthesis; (VI) riboflavin biosynthesis. Capital letters indicate the occurrence of open reading frames for these pathways in sequences from *Homo sapiens* (H), the yeast *Saccharomyces cerevisiae* (Y), the plant *Arabidopisis thaliana* (A), the bacterium *Escherichia coli* (E), and the molds *Physarum polycephalum* (P) and *Dictyostelium discoideum* (D). Small, crossed-out letters indicate the absence of these pathways in the respective species. The pteridine (pyrimido[4,5-b]pyrazine) bicyclic ring system is highlighted in red. For more details see text and Table S4.

COG1469, a form of GTP cyclohydrolase 1 with unrelated amino acid sequence detected in bacteria (El Yacoubi, et al. 2006) is absent in *P. polycephalum* sequences.

The GTP cyclohydrolase 1 reaction yields the common intermediate 7,8-dihydroneopterin triphosphate which is further converted by pathways (III), (IV) and (V) to tetrahydrofolate, tetrahydrobiopterin and 7-aminomethyl-7-deaza guanine, respectively. *Physarum* is unique in that it contains reading frames for all three pathways. *P. polycephalum* uses three different proteins to metabolize 7,8-dihydreoneopterin triphosphate to initiate the metabolism along pathways (III), (IV) and (V) using dihydroneopterin aldolase, 6-pyruvoyl tetrahydropterin synthase and 6-carboxy tetrahydropterin synthase enzymes, respectively.

(III) Tetrahydrofolate biosynthesis in *P. polycephalum* shows no special features, it is initiated by a trifunctional enzyme comprising the dihydroneopterin aldolase 6-hydroxymethyldihydropteridine diphosphokinase and dihydropteroate synthase activity, followed by a tetrahydrofolate synthase (folyl polyglutamate synthase) enzyme.

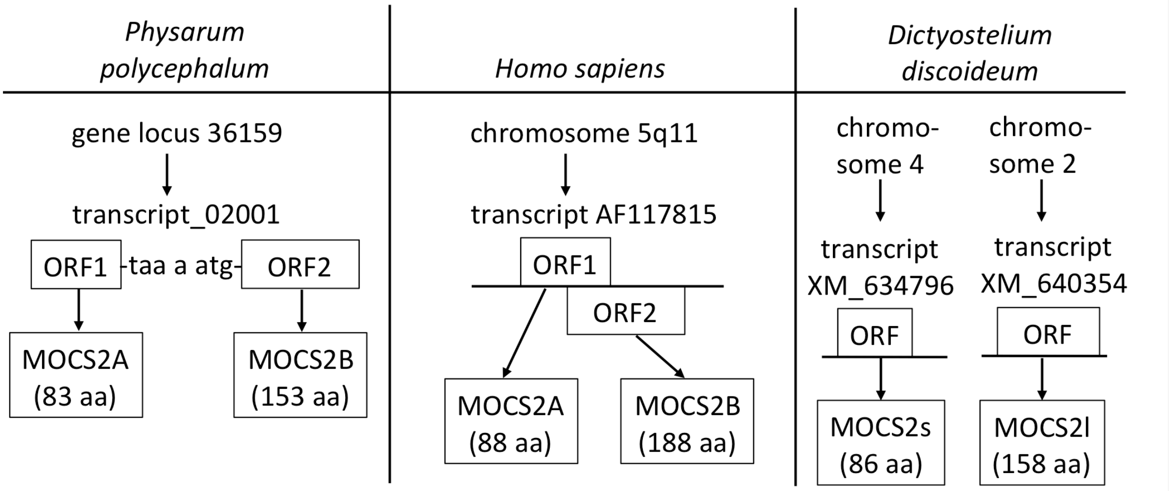

Figure S9. Transcripts for Molybdenum cofactor biosynthesis protein 2. *Physarum polycephalum* and *Homo sapiens* express bicistronic transcripts for the small and the large subunit, whereas *Dictyostelium discoideum* synthesizes two single reading frame messenger RNAs from two different chromosomes.

*(IV) Tetrahydrobiopterin biosynthesis*_from 7,8 dihydroneopterin triphosphate proceeds via two enzymes, 6-pyruvoyl tetrahydropterin synthase and sepiapterin reductase which are both present. Both enzymes required for recycling of the cofactor in its hydroxylation reactions, dihydropteridine reductase and 4a-hydroxytetrahydrobiopterin dehydratase, are also found.

*(V) 7-Aminomethyl deazaguanine biosynthesis:*_Reading frames for all four enzymatic steps are present. This is another remarkable finding since these biosynthetic enzymes are regularly found only in bacteria. 7-Aminomethyl 7-deazaguanine is a precursor of queuosine (Q), a special base incorporated into t-RNAs of most species, including humans who have to take up Q or Q precursors in the diet.

*(VI) Riboflavin biosynthesis:*_All genes required for this pathway are also found in *P. polycephalum,* including the key enzyme GTP cyclohydrolase 2. Riboflavin biosynthesis is different in plants and yeast. *Physarum* has reading frames only for the plant (*Arabidopsis thaliana*), not for the yeast (*Saccharomyces cerevisiae*) route. A remarkable feature so far unique among all sequences present in the non redundant protein database of NCBI by Febru-

ary 4, 2015 is that *P. polycephalum* has two trifunctional reading frames for enzymes to synthesize riboflavin, whereas other organisms have at least three or four separate though homologuous proteins to perform this task (Fig. S10). Again, like for nitric oxide synthases, GTP cyclohydrolases 1 and alkylglycerol monooxygenases, *P. polycephalum* encodes and expresses two additional apparently functional reading frames for lumazine synthase 1, although this enzyme is already encoded by one of the two trifunctional reading frames, Rib1 (see Table S5).

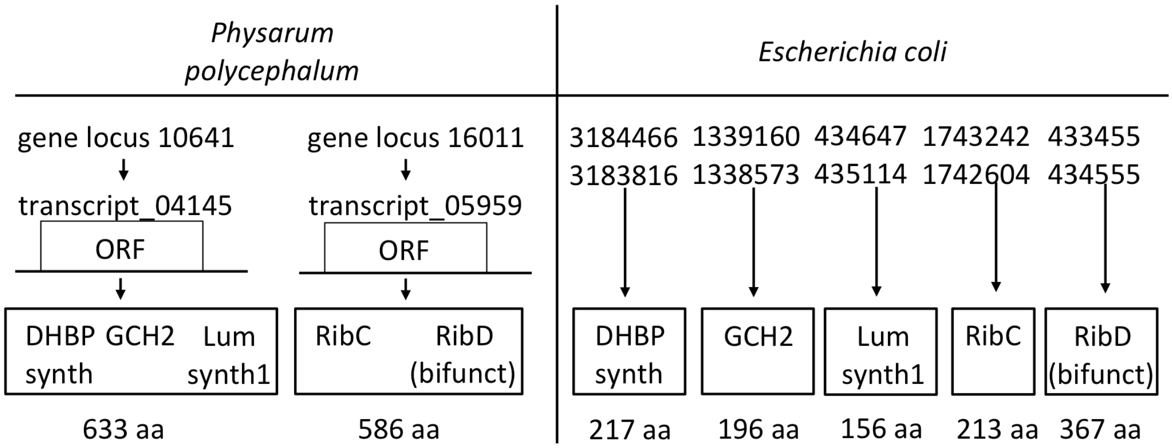

Figure S10. Riboflavin biosynthesis with two trifunctional proteins in *Physarum polycephalum*. DHBP synth, 3,4-dihydroxy-2-butanone 4-phosphate synthase (RibB); GCH2, GTP cyclohydrolase II (RibA); Lum synth1, lumazin synthase 1 (RibH). The numbers shown for *E. coli* refer to the K12-MG1655 genome, accession NC_000913.3.

Tetrahydrobiopterin-dependent enzymes, Reading frames for phenylalanine hydroxylase, two isoforms for calcium-independent nitric oxide synthase and two for alkylglycerol monooxygenases are present. No additional tyrosine and tryptophan hydroxylases are found. The occurrence of alkylglycerol monooxygenases as well as full length nitric oxide synthases comprising both reductase and oxygenase domains is quite remarkable, since both enzymes are regularly found only in animals (Gorren and Mayer 2007; Watschinger, et al. 2010).

Table S5. Pteridine metabolic open reading frames in *P. polycephalum*

Pathway (I) Molydbopterin synthesis

Gene	Description	EC-No	Evid ence (ref)	Phypoly_ Transcript number	Scaffold	Data-base	Closest Ortholog Species	Closest Ortholog Accession
MOCS1	GTP 8,9-lyase (cyclic pyranopterin mono-phosphate-forming) (MoaA + MoaC)	4.1.99.18		01245	3952	LULU	*Polysphondylium pallidum*	EFA85195
MOCS2 (2 ORFs in 1 mRNA)	mRNA 27-275; molybdopterin synthase, small subunit (MOCS2A; MoaD)<br>mRNA 277 - 735:molybdopterin synthase, large subunit (MOCS2B; MoaE)	2.8.1.12		02001	15260	3D6MA	*Acanthamoeba castel-lanii*<br>*Polysphondylium pallidum*	XP_004344729<br><br>EFA75022

Pathway (II) GTP cyclohydrolase 1

Gene	Description	EC-No	Evi-den-ce (ref)	Phypoly_ Transcript number	Scaffold	Data-base	Closest Ortholog Species	Closest Ortholog Accession
GCH1a*	GTP cyclohydrolase 1 isoform a	3.4.5.16	1	07412; 13920; 12199	9268	LULU	*Dictyostelium fascicu-latum*	XP_004361904
GCH1b*	GTP cyclohydrolase 1 isoform b	3.4.5.16		18235	43984	LULU	*Takifugu rubripes*	XP_003974407

Table S5, continued

Pathway (III) Tetrahydrofolate biosynthesis and recycling

Gene	Description	EC-No	Evi-den-ce (ref)	Phypoly_ Transcript number	Scaffold	Data-base	Closest Ortholog Species	Closest Ortholog Accession
FOL1 (trifunc-tional)	dihydroneopterin aldolase; 6-hydroxymethyldihydropteridine diphosphokinase; dihydropteroate synthase	4.1.2.25 2.7.6.3 2.5.1.15		05130	40396	LULU	*Polysphondylium pallidum*	EFA85424
FPGS	folyl polyglutamate synthase	6.3.2.17		06201	44132	LULU	*Polysphondylium pallidum*	EFA82253
DHFR	dihydrofolate reductase	1.5.1.3		15374	45368	N26B6 MA	*Psychroflexus gond-wanensis*	WP_003441104

Pathway (IV) Tetrahydrobiopterin biosynthesis and recycling

Gene	Description	EC-No	Evi-den-ce (ref)	Phypoly_ Transcript number	Scaffold	Data-base	Closest Ortholog Species	Closest Ortholog Ac-cession
PTS	6-pyruvoyl tetrahydropterin synthase	4.2.3.12		22227	13236	LULU	*Capsaspora owczar-zaki*	EFW43191.2
SR	sepiapterin reductase	1.1.1.153		13316	40857	LULU	*Capsaspora owczar-zaki*	XP_004348924
PCBD1	4a-hydroxytetrahydrobiopterin dehydratase	4.2.1.96		19752	15532	LULU	*Polysphondylium pallidum*	EFA77375
QDPR	quinoid dihydropteridine reductase	1.6.99.7	2	17368	36804	LULU	*Dictyostelium discoi-deum*	XP_644903

Table S5, continued

Pathway (V) 7-Aminomethy-7-deazaguanine (PreQ1) biosynthesis

Gene	Description	EC-No	Evi-dence (ref)	Phypoly_ Transcript number	Scaf-fold	Data-base	Closest Homologue Species	Closest Homologue Accession
QueD	6-carboxytetrahydropterin synthase	4.1.2.50		17564	S3S3K5_ 71144	3D6MA	*Cyanidioschyzon merolae*	BAM79613
QueE	7-carboxy-7-deazaguanine synthase	4.3.99.3		15658	13162	3D6MA	*Novosphingobium sp.*	YP_004534583
QueC	7-cyano-7-deazaguanine synthase	6.3.4.20		00668	39614	LULU	*Rhodospirillum rubrum*	YP_427044
QueF	7-cyano-7-deazaguanine reductase	1.7.1.13		10845	7455	LULU	*Moritella marina]*	WP_019442066

Pathway (VI) Riboflavin biosynthesis

Gene	Description	EC-No	Evi-dence (ref)	Phypoly_ Transcript number	Scaffold	Data-base	Closest Ortholog Species	Closest Ortholog Accession
Rib1 (trifunc-tional)	3,4-dihydroxy 2-butanone 4-phosphate synthase ;GTP cyclohydrolase 2 ;<br>lumazin synthase 1 isoform a	4.1.99.12<br>3.5.4.25<br>2.5.1.78		04145	8108	LULU	*Acanthamoeba castellanii*	XP_004340326
LUMS1b*	lumazin synthase 1 isoform b	2.5.1.78		21767	9142	LULU	*Acanthamoeba castellanii*	XP_004336543
LUMS1c*	lumazin synthase 1 isoform c	2.5.1.78		15152	33360	LULU	*Acanthamoeba castellanii*	XP_004336543
Rib2 (trifunc-tional)	riboflavin synthase;<br>riboflavin deaminase;<br>riboflavin reductase	2.5.1.9<br>3.4.5.26<br>1.1.1.193		05959	40361	LULU	aa 1-200: *Rhodanobacter thiooxydans*;<br>aa 200-583: *Parachlamydia acanthamoebae*	WP_008435929<br>YP_004652263

Table S5, continued

Tetrahydrobiopterin-dependent enzymes

Gene	Description	EC-No	Evi-ence (ref)	Phypoly_ Transcript number	Scaf-fold	Data-base	Closest Ortholog Species	Closest Ortholog Accession
PAH	phenylalanine hydroxylase	1.14.16.1		05554	3788	LULU	*Dictyostelium fascicu-latum*	XP_004366862
AGMO1a*	alkylglycerol monooxygenase isoform a	1.14.16.5		06381	2625	LULU	*Capsaspora owczar-zaki*	EFW43778.2
AGMO1b*	alkylglycerol monooxygenase isoform b	1.14.16.5		11597	5847	LULU	*Otolemur garnettii*	XP_003782767
NOS2a*	nitric oxide synthase, calcium independent, isoform a	1.14.13.39	3	18669	3435	LULU	*Gallus gallus*	NP_990292
NOS2b*	nitric oxide synthase, calcium independent, isoform b	1.14.13.39	3	00619	1699	LULU	*Crassostrea gigas*	EKC33784

*Genes annotated with a, b, c indicate protein isoforms which are expressed and highly likely to be functional as was experimentally confirmed for the *Physarum* nitric oxide synthase isoforms (NOS2a, NOS2b, Ref. 3)

Experimental evidence for the functional assignment

1. Golderer, G., Werner, E. R., Heufler, C., Strohmaier, W., Gröbner, P., Werner-Felmayer, G. (2001) GTP cyclohydrolase I mRNA: novel splice variants in the slime mold *Phy-sarum polycephalum* and in human monocytes (THP-1) indicate conservation of mRNA processing. *Biochem.J.* 355, 499-507

2. Wild, C., Golderer, G., Gröbner, P., Werner-Felmayer, G., Werner, E. R. (2003) *Physarum polycephalum* expresses a dihydropteridine reductase with selectivity for pterin substrates with a 6(1'2'-dihydroxypropyl) substitution. *Biol.Chem.* 384, 1057-1062

3. Messner, S., Leitner, S., Bommassar, C., Golderer, G., Grobner, P., Werner, E. R., Werner-Felmayer, G. (2009) *Physarum* nitric oxide synthases: Genomic structures and en-zymology of recombinant proteins. *Biochem.J.* 418, 691-700

8. Cell cycle regulation

8.1 Methods

For each protein family, we used HMMER3 (Eddy 2011) to create a profile Hidden Markov Model (HMM) with a training set taken from the cell cycle model organisms *Arabidopsis thaliana* and *Homo sapiens*. Sequences were first aligned with t_coffee using the mcoffee option (Wallace, et al. 2006), and the alignment was used to create a specific protein family profile HMM. Potential family members in each genome were uncovered using these profile HMMs with HMMER3. All putative hits in downloaded genomes were further validated (or rejected) using an online, iterative search algorithm (Jackhmmer) against the annotated SwissProt database (Finn, et al. 2011). The *P. polycephalum* cell cycle regulators found are assigned to transcript reference numbers and Gene ID numbers in Supplemental Spreadsheet 6. We classified sequences into Cyclin (Fig. S11) and Cdk (Fig. S12) sub-families by first aligning sequences with t_coffee using the mcoffee option, and using this alignment to build a phylogenetic tree with PhyML using LG+F+G options (Gouy, et al. 2010). We estimated the confidence of each branch node in Figs. S11 and S12 using the Shimodaira-Hasegawa approximate likelihood ratio test (SH-aLRT) (Anisimova and Gascuel 2006). The numbers of core cell cycle regulators and cell division kinases across eukaryotic genomes are compared in Tables S6 and S7, respectively.

8.2 **P. polycephalum** *cell cycle regulation compared to other organisms*

The natural synchrony of mitosis in large, multinucleate plasmodia has always been an attractive feature of *Physarum* cell biology. The degree of synchrony between distant nuclei and the rapid establishment of synchrony after fusing plasmodia in different cell cycle phases (Rusch, et al. 1966) is a consequence of vigorous shuttle streaming in macroplasmodia. In microplasmodia, before shuttle streaming is established, some degree of mitotic synchrony is maintained by traveling waves of mitosis (Haskins 2000), where a local accumulation of activated CycB/Cdk1 diffuses to neighboring regions of space and triggers the rapid activation of CycB/Cdk1 (hence, mitosis) in neighboring nuclei (Novak and Tyson 1993). Waves of nuclear division are also observed in fungal hyphae (Clutterbuck 1970) and in the shared cytoplasm of early animal embryos (Foe and Alberts 1983). Recent work on patterns of nuclear envelope breakdown in sperm nuclei dispersed in frog egg extracts maintained in long, thin capillary tubes provides convincing evidence for long-distance synchronization by mitotic trigger waves (Chang and Ferrell 2013).

Eukaryotes exhibit open mitosis (i.e. nuclear membrane breaks down during mitosis to allow spindle to capture chromosomes), partially open mitosis, or closed mitosis (i.e. nucleus stays intact and spindle forms inside the nucleus). Animal and plant cells usually have open mitosis, whereas fungi and unicellular eukaryotes have closed mitosis (Heath 1980). *Physarum* switches from open mitosis as a uninucleate amoeba to closed mitosis as a multinucleate plasmodium (Solnica-Krezel, et al. 1991). Closed mitosis is thought to be advantageous for syncytial nuclei with synchronous or parasynchronous mitosis to avoid attachment of chromosomes between the spindle of neighboring nuclei (De Souza and Osmani 2007). To this end, animal embryos with synchronous syncytial nuclei have evolved partially closed mitosis (Paddy, et al. 1996). This shows that regulation of nuclear envelope and spindle dynamics during mitosis is plastic. Over the coming decade, parallel studies between *Physarum* and

animal embryos should provide insights into mechanisms of mitotic synchrony, constraints of unicellular versus syncytial compartments, and the plasticity of open versus closed mitosis across the Unikonts.

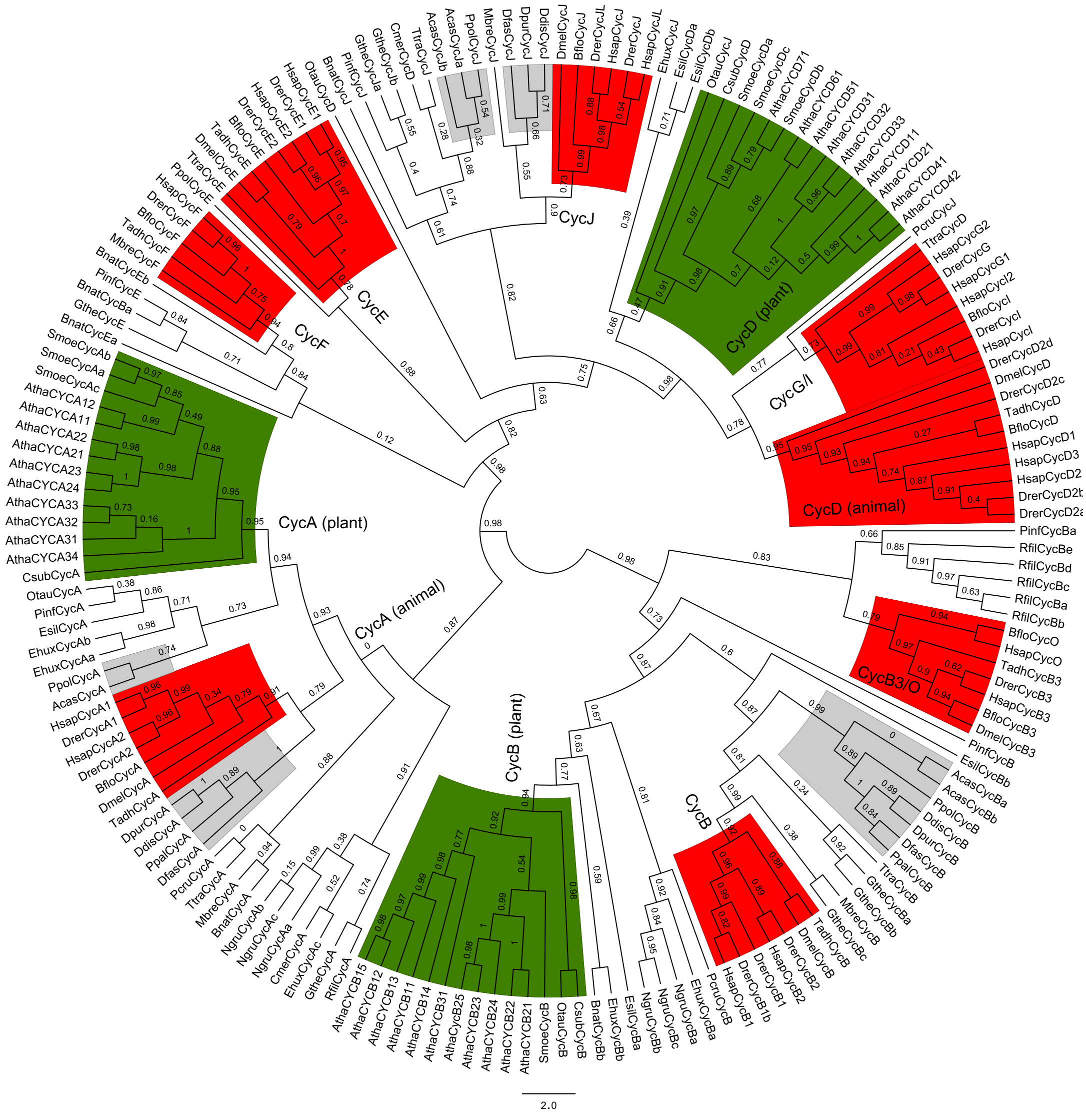

Figure S11. Molecular phylogeny of cyclins. We classified sequences into cyclin sub-families by first aligning sequences with t_coffee using the mcoffee option, and using this alignment to build a phylogenetic tree with PhyML using LG+F+G options. The confidence of each phylogenetic node was estimated using the Shimodaira-Hasegawa approximate likelihood ratio test (Gouy, et al. 2010). Opisthokonta (red), Amoebozoa (grey), Archaeplastida (green).

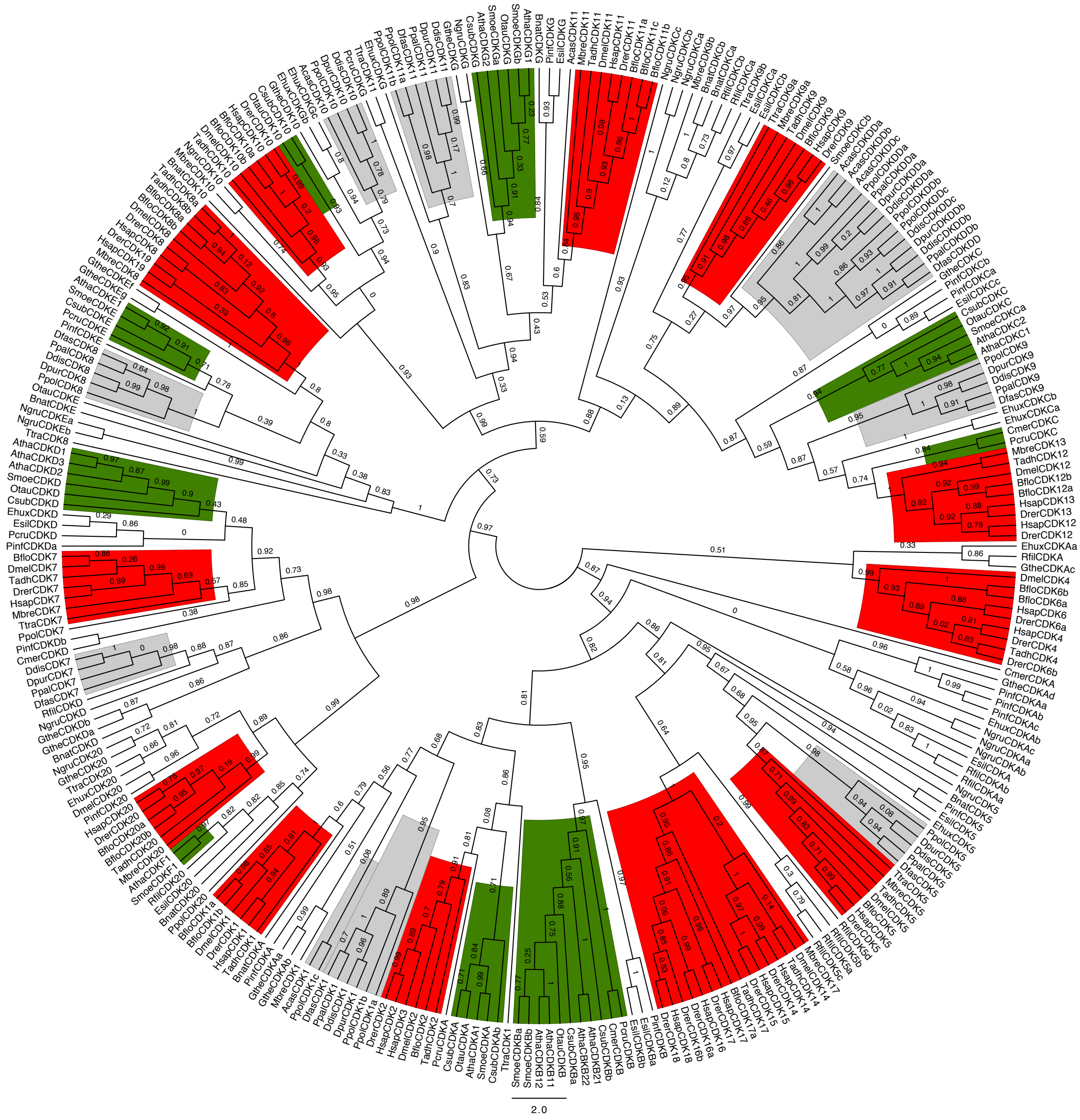

Figure S12. Molecular phylogeny of cell division kinases. We classified sequences into Cdk sub-families by first aligning sequences with t_coffee using the mcoffee option, and using this alignment to build a phylogenetic tree with PhyML using LG+F+G options. The confidence of each phylogenetic node was estimated using the Shimodaira-Hasegawa approximate likelihood ratio test (Gouy, et al. 2010). Opisthokonta (red), Amoebozoa (grey), Archaeplastida (green).

Table S6. Core cell cycle regulators across eukaryotic genomes. Each numerical entry specifies the number of family members discovered by our bioinformatic analysis. The cyclin homologues were further classified into sub-families based on molecular phylogeny (see Methods and Figure S11). The sub-family in each column is aligned with the gene name in *Homo sapiens* (top) and *Arabidopsis thaliana* (bottom). Opisthokonta (red), Amoebozoa (grey), Archaeplastida (green).

			(B3/O)		(E/F/J/G/I)		(Myt1)		(Cdh1)				
	(H. sapiens)	Cdk1-4,6	CycB	CycA	CycD	Cdc25	Wee1	Cdc20	Fzr1	E2F1-6	E2F7-8	DP	Rb
Metazoa	*H. sapiens (human)*	5	4	2	12	3	3	2	1	6	2	3	3
	D. rerio (zebrafish)	5	4	2	11	2	2	1	2	5	2	3	3
	B. floridae (lancelet)	5	2	1	5	1	2	1	2	2	2	1	2
	D. melanogaster (fly)	3	2	1	3	2	2	1	2	2		1	2
	T. adhaerens (placozoa)	3	2	1	3	1	1	1	1	2	1	1	2
Holozoa	*Monosiga brevicollis*	1	1	1	2	2	1	1	1	1	1	1	1
Apusozoa	*Thecamonas trahens*	1	1	1	3	2	1	1	1	1	1	1	1
Amoebozoa	*Dictyostelium discoideum*	1	1	1	1	1	3	1	1	1		1	1
	Dictyostelium purpureum	1	1	1	1	1	3	1	1	1		1	1
	Dictyostelium fasciculatum	1	1	1	1	1	3	1	1	1	1	1	1
	Polysphondylium pallidum	1	1	1		1	1	1	1	1	2		1
	Physarum polycephalum	3	1	1	2	3	4	2	1	1		1	1
	Acanthamoeba castellanii	1	2	1	2	2	2	5	1	1		1	1
Excavata	*Naegleria gruberi*	3	3	3		1	1	3	1	1	1	1	1
Cryptophyta	*Guillardia theta*	4	3	1	3	1	3	3		1	1	2	1
Haptophyta	*Emiliania huxleyi*	2	2	3	1		2	6	2	2			1
SAR	*Reticulomyxa filosa*	3	5	1		1	4	1	2				
	Bigelowiella natans	1	1	1	4	3	4	2	1	1	2		
	Phytophthora infestans	5	2	1	2	1	2	1	1	1	1	1	1
	Ectocarpus siliculosus	3	2	1	2		2	2	1	1	1	1	1
Archaeplastida	*Porphyridium cruentum*	2	1	1	1	1	3	1	1	1	1	1	
	Cyanidioschyzon merolae	2		1	1	1	1	1	1	1	1	2	1
	Ostreococcus tauri	2	1	1	2		1	1	1	1	1	1	1
	Coccomyxa subellipsoidea	4	1	1	1		2	1	1	1	1	1	1
	Selaginella moellendorffii	3	1	3	3		1	3	2	2	1	1	2
	Arabidopsis thaliana	5	11	10	11		1	6	3	3	3	2	1
	(A. thaliana)	CdkA/B	CycB	CycA	CycD		Wee1	Cdc20	Fzr1	E2FA-C	DEL	DP	RBR
					(SDS)								

Table S7. Cell division kinases across eukaryotic genomes. Each numerical entry specifies the number of CDKs discovered by our bioinformatic analysis. Cdk homologues were further classified into sub-families based on molecular phylogeny (see Methods and Figure S12). The sub-family in each column is aligned with the gene name in *Homo sapiens* (top) and *Arabidopsis thaliana* (bottom). Opisthokonta (red), Amoebozoa (grey), Archaeplastida (green).

	(H. sapiens)	Cdk1-4,6	Cdk14-18	Cdk5	Cdk7	Cdk20	Cdk8,19	Cdk10	Cdk11	Cdk9,12,13
Metazoa	*H. sapiens (human)*	5	5	1	1	1	2	1	1	3
	D. rerio (zebrafish)	5	6	1	1	1	2	1	1	3
	B. floridae (lancelet)	5	1	1	1	2	2	2	3	3
	D. melanogaster (fly)	3	1	1	1	1	1	1	1	2
	T. adhaerens (placozoa)	3	2	1	1	1	2	1	1	2
Holozoa	*Monosiga brevicollis*	1	1	1	1	1	1	1	1	3
Apusozoa	*Thecamonas trahens*	1		1	1	1	1		1	2
Amoebozoa	*Dictyostelium discoideum*	1		1	1		1	1	1	4
	Dictyostelium purpureum	1		1	1		1	1	1	3
	Dictyostelium fasciculatum	1		1	1		1		1	2
	Polysphondylium pallidum	1		1	1		1		1	3
	Physarum polycephalum	3		1	1	1	1	1	2	4
	Acanthamoeba castellanii	1						1	1	3
Excavata	*Naegleria gruberi*	3		1	1	1	2	1	1	3
Cryptophyta	*Guillardia theta*	4			2	1	2	1	1	1
Haptophyta	*Emiliania huxleyi*	2		1	1	1		2	1	2
SAR	*Reticulomyxa filosa*	3		4	1	1				2
	Bigelowiella natans	1		1	1	1	1	1	1	2
	Phytophthora infestans	5		1	2	1	1		1	2
	Ectocarpus siliculosus	3		1	1	1			1	3
Archaeplastida	*Porphyridium cruentum*	2			1		1		1	1
	Cyanidioschyzon merolae	2			1					1
	Ostreococcus tauri	2			1		1	1	1	1
	Coccomyxa subellipsoidea	4			1		1	1	1	1
	Selaginella moellendorffii	3			1	1	1		2	2
	Arabidopsis thaliana	5			3	1	1		2	2
	(A. thaliana)	CdkA/B			CdkD	CdkF1	CdkE		CdkG	CdkC

9. G-protein Coupled Receptors and Heterotrimeric G-proteins

Upon ligand binding G-protein coupled receptors (GPCRs) typically cause dissociation of an associated heterotrimeric G-protein into its alpha and beta-gamma subunits. Either of these subunits then interacts with a downstream component of the signal transduction cascade. GPCRs are subdivided into six families. The family 1 rhodopsin-like receptors consist, in addition to the light receptor rhodopsin, of receptors that bind chemical stimuli, ranging from cytokines to neuropeptides and opioids. The family 2 secretin-like receptors bind secretin and other hormones such as glucagon. The family 3 metabotropic glutamate receptors also contain Ca^{2+}-sensing receptors and GABA(B) receptors. Family 4 contains fungal pheromone receptors and family 5 the frizzled and smoothened receptors, involved in embryonic development. Family 6 contains the cAMP receptors (cARs) and cAR-like proteins (Bockaert and Pin 1999). Members from all six families were retrieved from a list of all transcribed *P. polycephalum* proteins using the Interpro identifier for each member. Phylogenetic trees for all families are shown in Fig. S13, with some well-characterized GPCRs from other organisms included in each family tree.

Compared to other amoebozoa, such as *D.discoideum* (Dd) and *A.castellani* (Ac), *P. polycephalum* has a very large number of GPCRs, exceeding Dd and Ac by factors of three and five, respectively (Table 3, main text). The large number of family 1 and 2 GPCRs (65 in total) is particularly striking, since these families have none to only few representatives in Dd and Ac. Family 3 is also well represented with 38 members against 0 in Ac. Of further interest are the 14 members of family 5. In Dd this family comprises 4 highly similar cAMP receptors and 9 cAR-like proteins, that bind other ligands. Only 1 *P. polycephalum* GPCR (Phypo_10246) groups with Dd cAR1 (Fig. S13E), but the long branch lengths separating the proteins highlight considerably sequence divergence. This reduces the likelihood that the *P. polycephalum* GPCR would detect cAMP. The large number of family 1 rhodopsin-like receptors suggest that light sensing rhodopsin could be present in *P. polycephalum*. However, none of the proteins is particularly close to bovine rhodopsin. In contrast to *D.discoideum*, *P. polycephalum* has no true multicellularity and therefore does not require its GPCRs for regulating its developmental programme. This implies that most of its very large array of GPCRs are used to detect environmental signals, such as chemicals secreted by cells of opposite mating type, prey, predators and putative symbionts, or physical stimuli such as light, humidity and mechanical perturbances. The most common target of GPCRs, the heterotrimeric G-proteins, are with 26 different alpha subunits also very abundant in *P. polycephalum,* compared to 12 in Dd and 5 in Ac (Table 3, main text). Phylogeny reconstruction with the 12 Dd alpha subunits, shows that the 9 of the Dd alpha subunits have likely orthologs in *P. polycephalum* (Fig. S14). These include GpaA, which affects the length/width ratio of fruiting body stalks (Dharmawardhane, et al. 1994), GpaB, which is required for both chemotaxis to cAMP and for generating cAMP pulses (Kumagai, et al. 1989) and GpaC, which is needed for gene expression induced by cAMP pulses (Brandon and Podgorski 1997). The other Dd orthologs have no or only subtle effects on the Dd developmental programme. The G-beta subunit consists of 6 WD40 repeats, which are also found in proteins unrelated to G-protein function. In Dd only GbpA has been proven to act as a G-protein beta subunit, and this protein has one ortholog in *P. polycephalum* (Fig. S14). There is also only a single G-protein gamma subunit in either organism (Fig. S14).

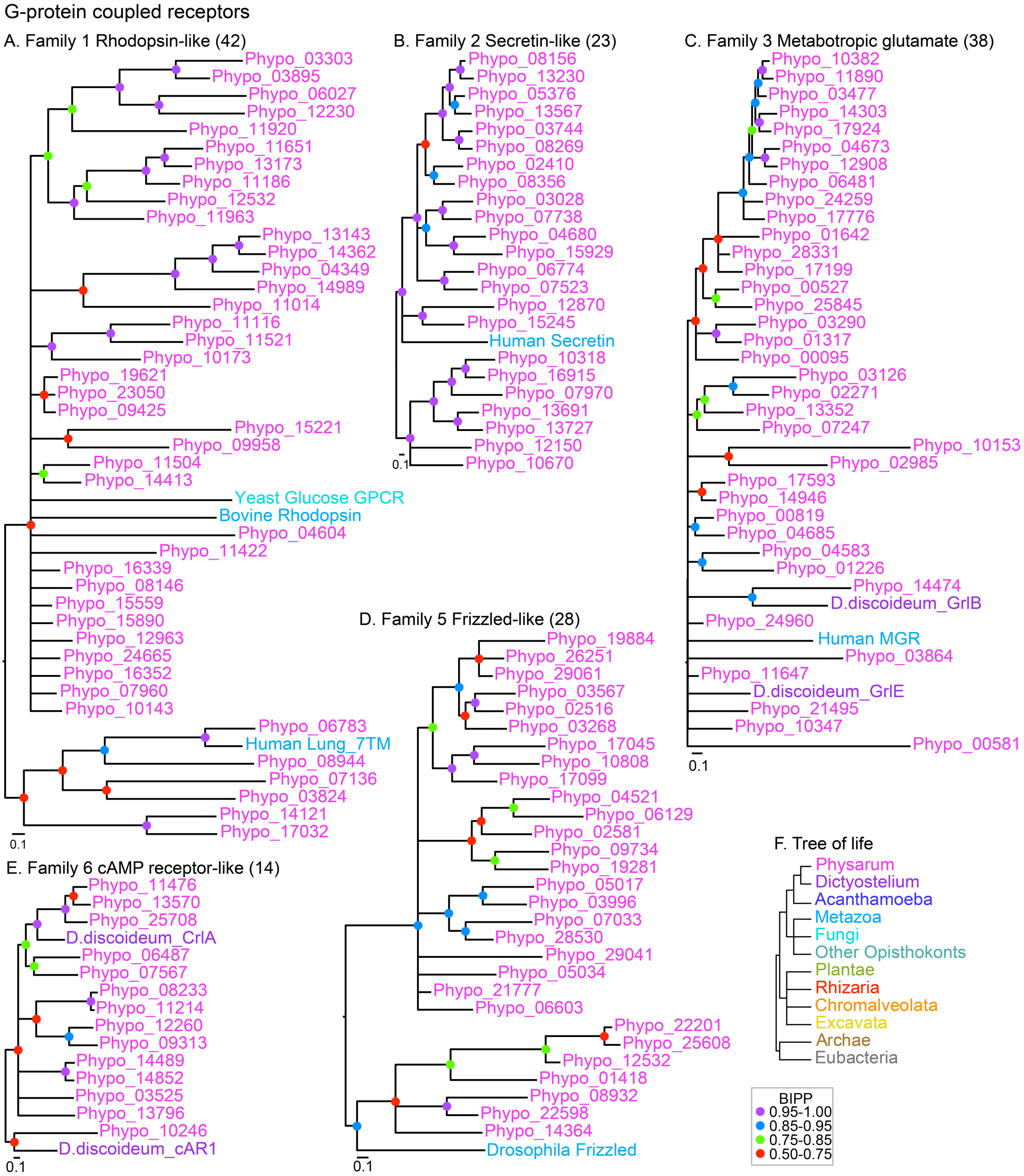

Figure S13. G-protein coupled receptor families. The list of 31708 translated *Physarum* transcripts was subjected to Interproscan (Jones, et al. 2014). The output was scanned for proteins harbouring Interpro domains IPR017452, IPR009637, IPR019336 and IPR023041 for GPCRs belonging to family 1, IPR000832 and IPR017981 for family 2, IPR000337, IPR002455, IPR017978 and IPR011500 for family 3, IPR020067 and IPR000539 for family 5 and IPR000848 for family 6. When proteins contained overlapping domain from different families, they were assigned to the family for which the domain scored the lowest E-value. For each family, protein sequences were aligned with some well-characterized members from other organisms using Clustal Omega with 5 combined iterations (Sievers and Higgins 2014). Large insertions and segments that did not align unambiguously were deleted. After selection of the amino-acid substitution model in TOPALi (Milne, et al. 2009), the alignments were subjected to Bayesian inference for phylogeny reconstruction (Ronquist and Huelsenbeck 2003). Analyses were run for 1 to 5 million generations and the resulting trees were rooted at midpoint (Hess and De Moares Russo 2007). Posterior probabilites (BIPP) of tree nodes are indicated by colored dots. Species names are color-coded as outlined in a cladogram for the tree of life in panel F. Genbank Accession: Yeast glucose GPCR: NP_010249; Bovine Rhodopsin: NP_001014890; Human Lung_7TM: Q5VW38;

Human secretin: NP_002971; Human metabotropic glutamate receptor (MGR): AAB05338; *D.discoideum* GrlE: XP_635160; *D.discoideum* grlB: XP_645480; *Drosophila* frizzled: CAA38458; *D.discoideum* CrlA: Q54U75; *D.discoideum* cAR1: P13773.

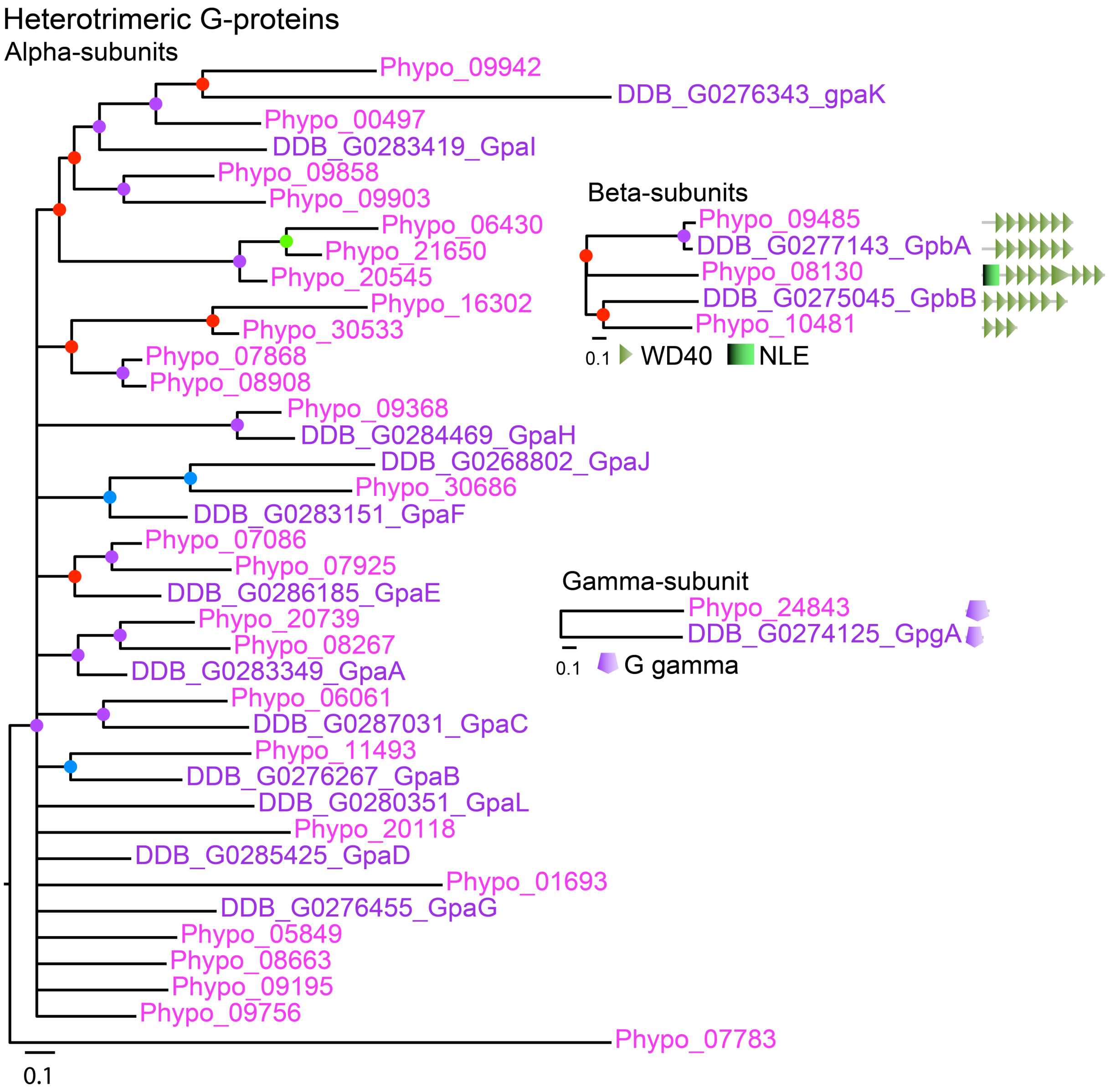

Figure S14. Heterotrimeric G-proteins. The sequences of proteins with Interpro domains IPR001019 and IPR001408 for G-alpha, IPR001632 and IPR016346 for G-beta, and IPR015898 for G-gamma subunits were individually aligned and subjected to phylogeny reconstruction as described in the legend to Figure SP1. Sequences for all *D. discoideum* G-protein alpha, beta and gamma subunits were included in the alignments. The G-beta and G-gamma phylogenies are annotated with the functional domain architectures of the proteins as determined by SMART (Schultz, et al. 1998).

10. Sensor Histidine Kinases and Phosphatases

A second important class of receptors for external stimuli are the sensor histidine kinases/phosphatases (SHKPs). SHKPs are most abundant in prokaryotes, where they detect a broad range of external and internal chemical or physical stimuli. To a much lesser extent, they are also used by plants and fungi, but are not found in metazoa (Wolanin, et al. 2002). We identified no less than 51 SHKPs in the *Physarum* genome (Fig 4, main text). This is significantly more than the 16 SHKPs present in Dd (Heidel, et al. 2011), but similar to the 48 SHKPs detected in Ac (Clarke, et al. 2013).

The majority of proteins harbour the bipartite HisKA phosphoryl acceptor/dimerization domain and the HATPase-c that make up the catalytic core of the SHKPs, well as one or more receiver domains and HPT (histidine phospho-transfer) domains, which serve as intermediates for forward or reverse histidine to aspartate phosphorelay that is initiated by respectively the histidine kinase or phosphatase activity of the catalytic core. In addition, most SHKPs contain a broad range of other functional domains that acts as receptors which regulated the activity of the catalytic core. Very prominent are GAF domains, which can sense cyclic nucleotides and light (Aravind and Ponting 1997) and PAS/PAC domains, which sense oxygen, redox potential and light, mostly by interacting with a haeme of a flavin molecule (Taylor and Zhulin 1999). Several of the SHKPs with GAF or PAS/PAC domains also harbour an N-terminal serine/threonine/tyrosine protein kinase domain and/or an AAA-ATPase domain. Since both protein kinases and AAA-ATPases regulate a broad range of cellular functions (Iyer, et al. 2004), the significance of the presence of these domains in SHKPs is not clear.

Of particular interest are two SHKPs with the red/far red light sensing phytochrome domain (Essen, et al. 2008). The process of sporulation in *P. polycephalum* is triggered by light and this response was previously shown the be mediated by a receptor with the properties of phytochrome (Marwan and Starostzik 2002; Starostzik and Marwan 1995b). Only 10 of the *P. polycephalum* SHPKs harbour transmembrane domains. This implies that the remaining 41 either detect membrane-permeant extracellular signals or signals that are generated inside the cell.

The target of SHKP-activated phosphorelay is usually a receiver linked to an effector, such as an enzyme or a transcription factor. We detected 10 proteins with receiver domains that were not SHKPs (Fig. S15). Only two of those carried an identifiable effector: the *P. polycephalum* RegA-like cAMP phosphodiesterases. This suggests that similar to the role of SHKPs in *Dictyostelium* (Schaap 2011a), the regulation of cAMP levels is a major target for the *P. polycephalum* SHKPs. However, it is quite possible that the other receivers regulate effectors by intermolecular interactions. At present we can also not rule out that some of the stand-alone receivers are incomplete sequences that are actually part of an SHKP.

Several SHKPs harbour an intramolecular enzyme domain, such as a serine/threonine/tyrosine protein kinase domain, a peptidase domain, an AAA-ATPase domain or a methylesterase domain. These SHKPs could regulate the activity of these intrinsic enzymes by intramolecular phosphorelay. Another point of interest is that only two of the *P. polycephalum* SHKPs have one of the 15 *Dictyostelium* SHKPs as their closest homolog, and that most *P. polycephalum* SHKPs are more closely related to each other than to SHKPs from other organisms. This suggests that the *P. polycephalum* SHKPs are the result of considerable gene family expansion within the myxogastrids.

In conclusion, it is likely that SHKPs play a major role in controlling *P. polycephalum* physiology and development in response to physical and chemical stimuli and that many of these responses are controlled by regulation of intracellular cAMP levels.

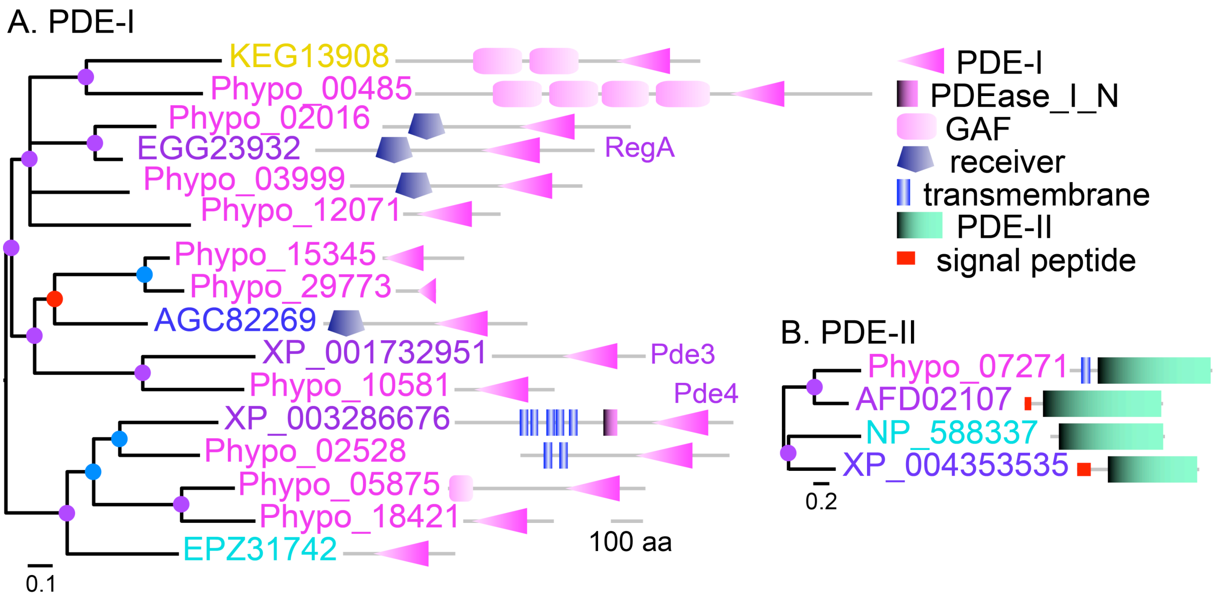

Figure S15. Cyclic nucleotide phosphodiesterases. Proteins sequences harbouring the IPR002073, IPR023174 and/or IPR023088 3'5'-cyclic nucleotide phosphodiesterase conserved and catalytic domains were retrieved from Interproscan of all transcribed coding sequence. The *Physarum* genome was additionally were queried by BLAST with all Dictyostelid PDE-I and PDE- II sequences and with metazoan PDE-I and fungal PDE-II sequences. Phylogenetic trees were constructed from the retrieved sequences and several of their closest homologs in Genbank as outlined in the legend to Figure S13. Gene identifiers are color coded as indicated in Figure S13.

11. Cyclic Nucleotide Signaling

The cyclic nucleotides cAMP and cGMP are well known intracellular signalling intermediates for extracellular signals in all domains of life. In particular, cAMP has major roles in controlling developmental processes, such as sporulation, encystation, aggregation, and morphogenesis in Dictyostelid social amoebas (Loomis 2014; Schaap 2011b), while cGMP has a more restricted function in cytoskeletal remodeling (Veltman, et al. 2008). At least some of the roles of cAMP or cGMP in Dictyostelids are likely to be conserved in *Physarum.*

11.1 Cyclic nucleotide synthesis: adenylate- and guanylate cyclases

cAMP and cGMP are synthesized from ATP and GTP by adenylate- and guanylate cyclases, respectively. In contrast to bacteria, which have five structurally distinct catalysts for cyclic nucleotide synthesis, eukaryotes are thus far only known to use the type III nucleotidyl cyclases, which are also abundantly present in prokaryotes (Linder and Schultz 2003).

To identify the full spectrum of nucleotidyl cyclases in *P. polycephalum*, we performed Blast searches of all transcriptome and genome sequences using the five Dictyostelid cyclases ACA, ACG, AcrA, GCA and SGC (Saran, et al. 2002) and the 10 most diverse cyclase

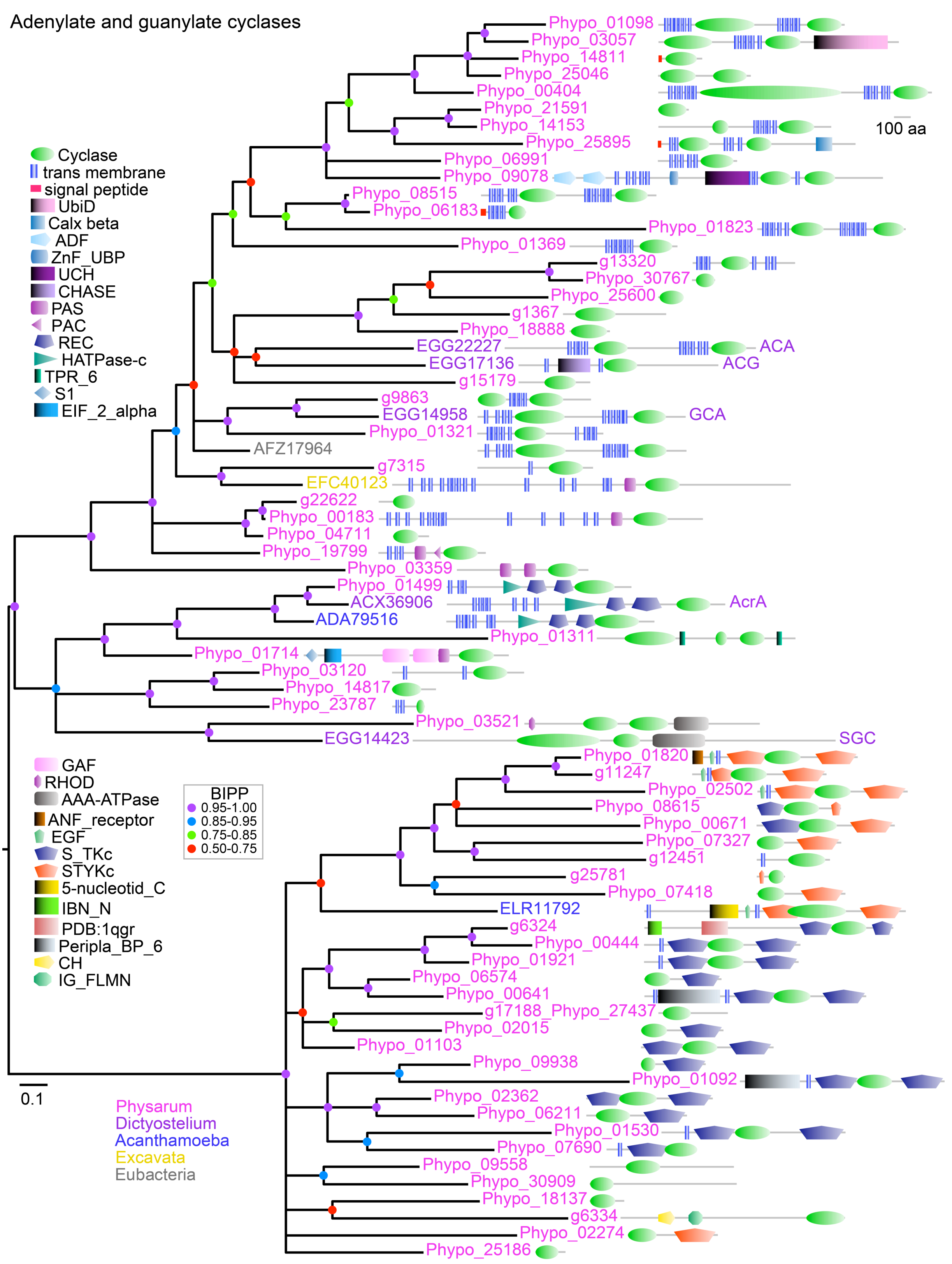

Figure S16. Nucleotidyl cyclases. The *P. polycephalum* genome was queried by BLAST with sequences encoding Dictyostelid cyclase domains and the 10 most divergent cyclase domains, while an Interproscan of all transcribed proteins was queried for proteins containing the Interpro IPR001054 adenylyl cyclase class-3/4/guanylyl cyclase domain. As for SHKPs, the retrieved sets overlapped, but the largest number of cyclases were found in

the transcriptome. An alignment of all identified *Physarum* cyclase proteins and some of their best homologs in other organisms was used for phylogeny reconstruction as described above. The tree is midpoint-rooted and annotated with the SMART domain architecture of the proteins.

domains in Genbank as bait and we retrieved all proteins containing the Interpro IPR001054 adenylyl cyclase class-3/4/guanylyl cyclase domain from an Interproscan of all translated transcripts. A total of 64 proteins with nucleotidyl cyclase domains were identified. Phylogenetic inference from the aligned protein sequences separates these protein in two major branches (Fig. S16). The lower branch consists mainly of complete and partial proteins in which the cyclase domain is flanked by two protein kinase domains. This region which likely resides in the cytosol is separated by a N-terminal transmembrane domain from a putative extracellular region, which often contains an EGF domain and a variety of other domains. The *Acanthamoeba* genome contains a heavily expanded family of 67 adenylate cyclases with a similar N-terminal extracellular region and a cytosolic region with two protein kinase domains flanking the cyclase domain (Clarke, et al. 2013). A representative of this family (ELR11792) groups closely with the 22 *P. polycephalum* cyclase/kinase proteins. The remaining proteins in the lower branch have mostly only a cyclase domain.

The upper branch consist of around 13 proteins that resemble the major class of metazoan adenylate cyclases with 2 cyclase domains, interspersed with 2 sets of 6 transmembrane domains. The *Dictyostelium* adenylate cyclase ACA and guanylate cyclase GCA also belong to this class. Additionally *P. polycephalum* has 3 cyclases with PAS, PAC or GAF sensor domains (Aravind and Ponting 1997; Ponting and Aravind 1997) and 4 cyclases with unusual domains (for cyclases) involved in either actin remodeling, ubiquitination or calcium export. Note that some of these exotic domain combinations could be due to errors in DNA sequence assembly or gene model prediction. No obvious homolog of *Dictyostelium* ACG was found, but an AcrA homolog (Phypo_01499), complete with additional histidine kinase and receiver domains is present in *P. polycephalum*. The *Dictyostelium* guanylate cyclase GCA has two close relatives in *P. polycephalum*, while SGC, another guanylate cyclase has one relative. In conclusion, *P. polycephalum* has an astonishing and unmatched number of architecturally different enzymes for cAMP and cGMP synthesis. cGMP has a substantiated role in the induction of sporulation in *P. polycephalum* (Golderer, et al. 2001) and there is sporadic evidence of roles for cAMP in cell division, motility and gravity sensing (Block, et al. 1998; Kuehn 1972; Matveeva, et al. 2012). However, the abundance and variety of nucleotidyl cyclases in *P. polycephalum* indicates that importance of cAMP and cGMP as second messengers in this system have been vastly underestimated.

11.2 Cyclic nucleotide detection: cNMP binding domains

Intracellular cAMP and cGMP are both mostly detected by the highly conserved cyclic nucleotide (cNMP) binding domain of target proteins. In prokaryotes this domain is part of the catabolite repressor, a transcription factor that is regulated by cAMP. In metazoa, two cNMP binding domains make up the regulatory (R) subunit of cAMP dependent protein kinase (PKA). However, the cNMP domain is also found as part of cNMP-regulated ion channels, GTP-exchange factors, called EPACs, and cNMP phosphodiesterases (Kopperud, et al. 2003; Meima, et al. 2002). Cyclic nucleotides can also be detected by GAF domains (Schultz 2009), but these domains also bind unrelated compounds and are therefore not diagnostic for cNMP signalling.

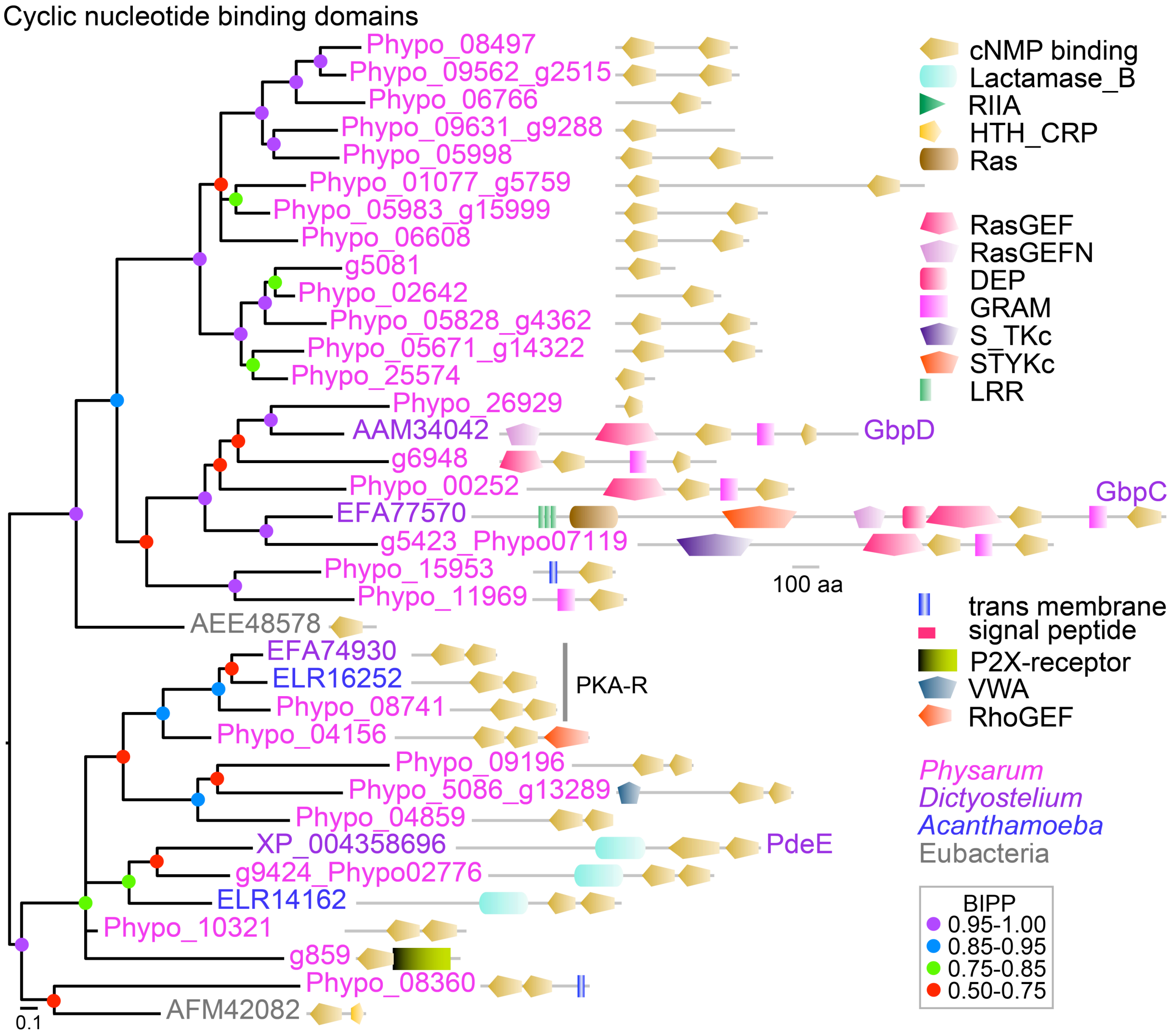

Figure S17. Cyclic nucleotide binding domains. The *P. polycephalum* genome was queried by BLAST with sequences encoding Dictyostelid cNMP domains, prokaryote CRP and mammalian PKA-R and EPAC domains. The output of Interproscan output of all transcribed proteins was queried for proteins containing the IPR000595 cyclic nucleotide-binding domain. The *Physarum* proteins were aligned with close homologs in other organisms and phylogenetic relationships between the sequences were determined by Bayesian inference (Ronquist and Huelsenbeck 2003). The resulting tree was rooted at mid-point and annotated with the domain architecture of the proteins as determined with SMART (Schultz, et al. 1998). Posterior probabilities of tree nodes are indicated by coloured dots.

The *P. polycephalum* genome contains no less than 28 cNMP binding domain proteins (Fig. S17), compared to 5 in *Dictyostelium* and 7 in *Acanthamoeba* (Clarke, et al. 2013; Heidel, et al. 2011). Among these proteins is one likely PKA-R candidate (Phypo_08741) and one protein similar to *Dictyostelium* PdeE, a cAMP stimulated cAMP phosphodiesterase (Meima, et al. 2003). There are also three proteins that contain a Ras-GTP exchange factor (Ras-GEF) domain and additional protein kinase (S_TKc, STYKc), Ras-GEFN or GRAM domains (g6948, Phypo_00252, g5423). This array of domains is also found in two Dictyostelid cGMP binding proteins, GbpC and GbpD, which are the closest current relatives of g5423 and g6948. The *Dictyostelium* proteins are involved in chemotaxis and response to osmotic stress, processes in which cGMP has a second messenger function (Marin, et al. 2008).

There are many novel arrangements, such as 8 proteins with two widely spaced cNMP domains, a PKA-R related protein (Phypo_04156) with additional RhoGEF domain and a protein combining two cNMP domains with a Von Willebrand Factor A (VWA) domain. The original VWA factor is a multimeric glycoprotein involved in blood clotting (Ruggeri and Ware 1993). In protein g859 the cNMP binding domain is combined with half of a purinergic P2X receptor region (MacKenzie, et al. 1999).

The number of cNMP domain proteins in *P. polycephalum* and the range of different combinations of cNMP domains with other protein functional domains is unparalleled in eukaryotes. Combined with the diversity and abundance of nucleotidyl cyclases, the diversity in cNMP domains proteins strongly suggests that cAMP and cGMP have many different roles in the *P. polycephalum* life cycle and that several of these roles may be unique to this organism.

11.3 Cyclic nucleotide degradation: phosphodiesterases

Cellular cAMP or cGMP levels are stringently controlled by cyclic nucleotide phosphodiesterases, which hydrolyse these molecules to 5'AMP and 5'GMP, respectively. Metazoa only use type I cyclic nucleotide phosphodiesterase with a conserved HDc domain. Other eukaryotes, such as fungi and Dictyostelids, additionally use the low affinity PDE-II type enzymes with HSHLDH motif (Conti and Beavo 2007), while the Dictyostelid PDE-III type enzymes, PdeD and PdeE, carry a related HCHADHDS motif that is however structurally more related to the β-lactamase_II protein family (Meima, et al. 2002). The latter proteins also harbour conserved cNMP binding domains and their single *P. polycephalum* homologue (g9424) is incorporated into Fig. S15.

We detected a single PDE-II (Phypo_07271) and 10 PDE-I type phosphodiesterases in the *P. polycephalum* genome. One gene was closely related to the *Dictyostelium* cAMP phosphodiesterase PDE4 and another to the *Dictyostelium* cGMP phosphodiesterase PDE3 (Bader, et al. 2007). One *Physarum* PDE-I protein harbours a single N-terminal GAF domain (Phypo_05875), while another (Phypo_00485) harbours four N-terminal GAF domains. In addition to other small molecules, GAF domains can selectively bind cAMP and/or cGMP. In mammalian PDE-I enzymes, the GAF domains function as allosteric regulators of the phosphodiesterase activity (Hurley 2003). The presence of two cNMP regulated PDE-I type enzymes, in addition to a likely cAMP-regulated PDE-III activity, highlights considerable fine-tuning of cyclic nucleotide hydrolysis in *P. polycephalum.*

Two *P. polycephalum* PDE-I genes harbour an N-terminal receiver domain and are closely related to the *Dictyostelium* cAMP phosphodiesterase RegA, which plays a pivotal role in controlling the encapsulation of stalk- and spore cells (Shaulsky, et al. 1998; Thomason, et al. 1998). The RegA receiver domain is the single characterized target for phosphorylation/dephosphorylation by sensor histidine kinases/phosphatases (SHKPs). In *Dictyostelium* several of these SHKPs are receptors for secreted signals that control timely spore- and stalk encapsulation as well as the germination of the spores (Singleton, et al. 1998; Wang, et al. 1996; Zinda and Singleton 1998). With this in mind, it is particularly interesting that two *Physarum* SHKPs harbour a phytochrome domain, which was previously shown to mediate light-induced sporulation (Starostzik and Marwan 1995b). It suggests that similar to *Dictyostelium* sporulation (Loomis 2014), *P. polycephalum* sporulation could also be triggered by increased cAMP levels and PKA activation.

12. Protein Kinases

In eukaryotes, the protein kinases that phosphorylate other proteins on serine/threonine (S/T) or tyrosine (Y) residues are the most ambiguous components of signal transduction cascades. Their substrate specificity is dictated by the peptide sequence flanking the S/T or Y residues, but also by other structural features of the target protein and its environment. Humans have a total of 518 different protein kinases and even yeast has over a 100 protein kinases (Hanks 2003). All expressed *P. polycephalum* proteins were analysed using Interproscan 5 (Jones, et al. 2014) for the presence of the S/T or Y kinase domains. A total of 1925 protein kinase domains were recognized using the PFAM, SMART, PrositePattern, PrositeProfile and PRINTS Hidden Markov Models for these domains, but this included many replicate entries of the same domain (Supplemental Spreadsheet 7, sheet 1 "Protein_kinases"). The number of unique proteins that harbour S/T, S/T/Y and/or Y domains is 447, which approaches the number of protein kinases in humans. Among other amoebozoa, *Dictyostelium* has 295 protein kinases (Eichinger, et al. 2005) and *Acanthamoeba* 377 (Clarke, et al. 2013). In addition to S/T and Y specific kinases, there are also protein kinases with dual specificity (S/T/Y) and we isolated the three subsets by their Interpro identifiers (Supplemental Spreadsheet 7, sheets 2-4). The number of proteins carrying these domains is listed in Table 3 (main text).

The total number of proteins with S/T, S/T/Y or Y domains adds up to 519 instead of 447, which means that there are 172 proteins with either multiple kinase domains or in which the same domain is recognized by both S/T, S/T/Y and/or Y models.

The presence of many serine/threonine or dual-specificity protein kinases is common to all eukaryotes and therefore does not stand out as a feature specific to *P. polycephalum*. However, the 29 proteins identified as tyrosine kinases deserve further scrutiny. Until recently tyrosine-specific protein kinases (TKs) were considered to be exclusively present in Metazoa, where they play a dominant role in regulating cellular responses to growth factors, cytokines and insulin (Lim and Pawson 2010). TKs are not present in *Dictyostelium*, but 21 TKs were recently detected in the *Acanthamoeba* genome (Clarke, et al. 2013).

The Interpro and other domain definitions fall short of distinguishing between true tyrosine kinase domains and tyrosine kinase like domains, and we therefore inspected a sequence alignment of the 29 putative *P. polycephalum* tyrosine kinases for conserved residues implicated in peptide-substrate recognition.

These residues are

```
HrDlAaRN
   RtA
```

and

```
lPikWmapE
f vr tpl
```

in subdomains VIB and VIII, respectively, with residues in upper-case being strictly conserved (Hanks 2003). Of the set of 29 tyrosine kinases, 4 conformed completely to the consensus and another 5 had less than 3 deviations (Supplemental Spreadsheet 7, sheet 4). For this set of 9 proteins, we retrieved their closest homologs in other organisms by BLASTp query of UniprotKB (Magrane and Consortium 2011) and aligned all proteins with the structurally characterized human tyrosine kinase ZAP-70. Fig. 5A (main text) shows the alignment of subdo-

mains VIB and VIII of all proteins, with tyrosine kinase specific residues outlined in red text. The full alignment was used to construct a phylogenetic tree of the proteins (Fig. 5B, main text), which was annotated with the protein domain architecture.

Three of the four *P. polycephalum* TKs with full TK consensus (underlined) grouped together with metazoan TKs. The *P. polycephalum* proteins all harboured a single transmembrane domain, suggesting that they could be receptor tyrosine kinases. One protein also contained a signal peptide and several parallel beta-helix (PbH1) repeats. The fourth protein grouped with an *Acanthamoeba* TK, and contained Ring and TRAF zinc finger domains and a SPRY domain of unknown function. The putative *Physarum* TKs that lacked full consensus grouped mostly with protein kinases of Dictyostelia or plants, neither of which is known to possess true TKs (Lim and Pawson 2010). The remaining protein belongs to a larger family of adenylate cyclases, flanked by protein kinase domains, that are very abundant in *Acanthamoeba* (Clarke, et al. 2013). Similar to its *Acanthamoeba* homolog, this protein actually contained a lysine at position 5 of subdomain VIB, which typifies S/T kinases, and it is therefore definitely not a tyrosine kinase. In conclusion, *Physarum* appears to have four genuine TKs, of which three bear greatest similarity to metazoan TKs.

In metazoa, the most common consequence of tyrosine phosporylation is binding of a src homology 2 (SH2) domain to the tyrosine phosphorylated protein. The SH2 domain is mostly associated with a range of other protein-protein interaction domains, enzymes or DNA binding domains, which further transduce the signal (Liu and Nash 2012). We detected a total of 18 proteins that contained an SH2 domain in *P. polycephalum* (Fig. 6, main text). Ten proteins contained an additional S/T kinase domain and three proteins contained a Dict_STAT-coil domain that is also present in the *Dictyostelium* STAT transcription factors and is similar to the DNA binding domain of metazoan STAT transcription factors. The metazoan STATs mainly mediate cell proliferation and immune response triggered by cytokines, while the *Dictyostelium* STATs regulate gene expression in response to external signals such as cAMP, DIF-1 and osmotic stress (Williams 2006). Three *Physarum* SH2 domain proteins have no additional domains, one protein harbours leucine-rich repeats (LRRs) that commonly function in protein-protein interactions and one protein is highly similar to the conserved SPT6 transcription elongation factors that have a similar array of functional domains (Kwak and Lis 2013).

In short, with 4 tyrosine kinases and 18 SH2 domain proteins, *Physarum* has well-developed tyrosine kinase signalling compared to *Dictyostelium,* which has no true tyrosine kinases and 15 SH2 domain proteins. Both *P. polycephalum* and *Dictyostelium* belong to Conosa, one of the two major subdivisions of Amoebozoa (Schilde and Schaap 2013). *Acanthamoeba*, which is a member of the other subdivision, Lobosa, has no less than 22 tyrosine kinases and 48 SH2 domain proteins. This means that tyrosine kinases and SH2 domain proteins were already present in the last common ancestor of Lobosa and Conosa and that tyrosine kinases were lost from the Dictyostelid lineage.

13. Photoreceptors

Although the control of cell motility and morphogenesis by light (photomovement, phototaxis) is well-known in *Dictyostelium* (Häder and Poff 1979a, b) and *P. polycephalum* (Häder and Schreckenbach 1984; Schreckenbach 1984), the molecular identification and functional assignment of active photoreceptors is pending. Surprisingly, the molecular identity of the photoreceptor mediating phototaxis or photomorphogenesis in *Dictyostelium* has not been revealed through the genome project (Eichinger, et al. 2005; Jékely 2009). The *Acanthamoeba* genome project identified two microbial-type rhodopsins that carry carboxy-terminal histidine kinase and receiver domains (Clarke, et al. 2013), clearly suggesting them to be sensory photoreceptors but their specific physiological function has not yet been demonstrated.

P. polycephalum photoreceptors have been relatively well characerized with respect to their spectral properties and physiological functions but hitherto not at the molecular level. There are at least two distinct photoreceptors that control sporulation of starving plasmodia, a constitutively present specific blue light photoreceptor (Starostzik and Marwan 1995a) and a phytochrome-like photoreceptor that is induced during starvation of plasmodial cells, which was the second phytochrome discovered outside the plant kingdom (Mooney and Yager 1990; Starostzik and Marwan 1995b). The *P. polycephalum* phytochrome with its far-red red reversible photocycle can be detected spectroscopically in intact plasmodial cells (Lamparter and Marwan 2001). A phytochrome-like photoreceptor also controls plasmodial phototaxis (Nakagaki, et al. 1996).

Sporulation of starved *P. polycephalum* plasmodia is induced by far-red light and the induction is reverted by red light (Starostzik and Marwan 1995b). However, spectroscopic evidence indicates that approximately 90 % of the photoreversible phytochrome is present in the red light-absorbing P_r form in plasmodia that have never been exposed to visible light (Lamparter and Marwan 2001). The effectiveness of far-red light in inducing sporulation and its complete reversibility by red suggests that a physiological relevant amount of phytochrome is syntesized in the dark in its P_{fr} form. It is noteworthy that the sensitivity of starved plasmodia to far-red light in triggering sporulation can be significantly enhanced by a pre-exposure to red light. One possible explanation would be the presence of two different phytochromes that act synergistically, one specifically synthesized as P_r the other as P_{fr} in the dark (Lamparter and Marwan 2001). Whether this is indeed the case for PhyA and PhyB and whether the deletion upstream of the conserved PASDIPPQARRL motif in PhyA as compared to PhyB is responsible for an according difference remains to be established. Sequence alignments of *P. polycephalum* phototropin, cryptochrome, and the phytochromes with their closest related orthologs in the uniprot database are shown in Figs. S18 to S20, respectively.

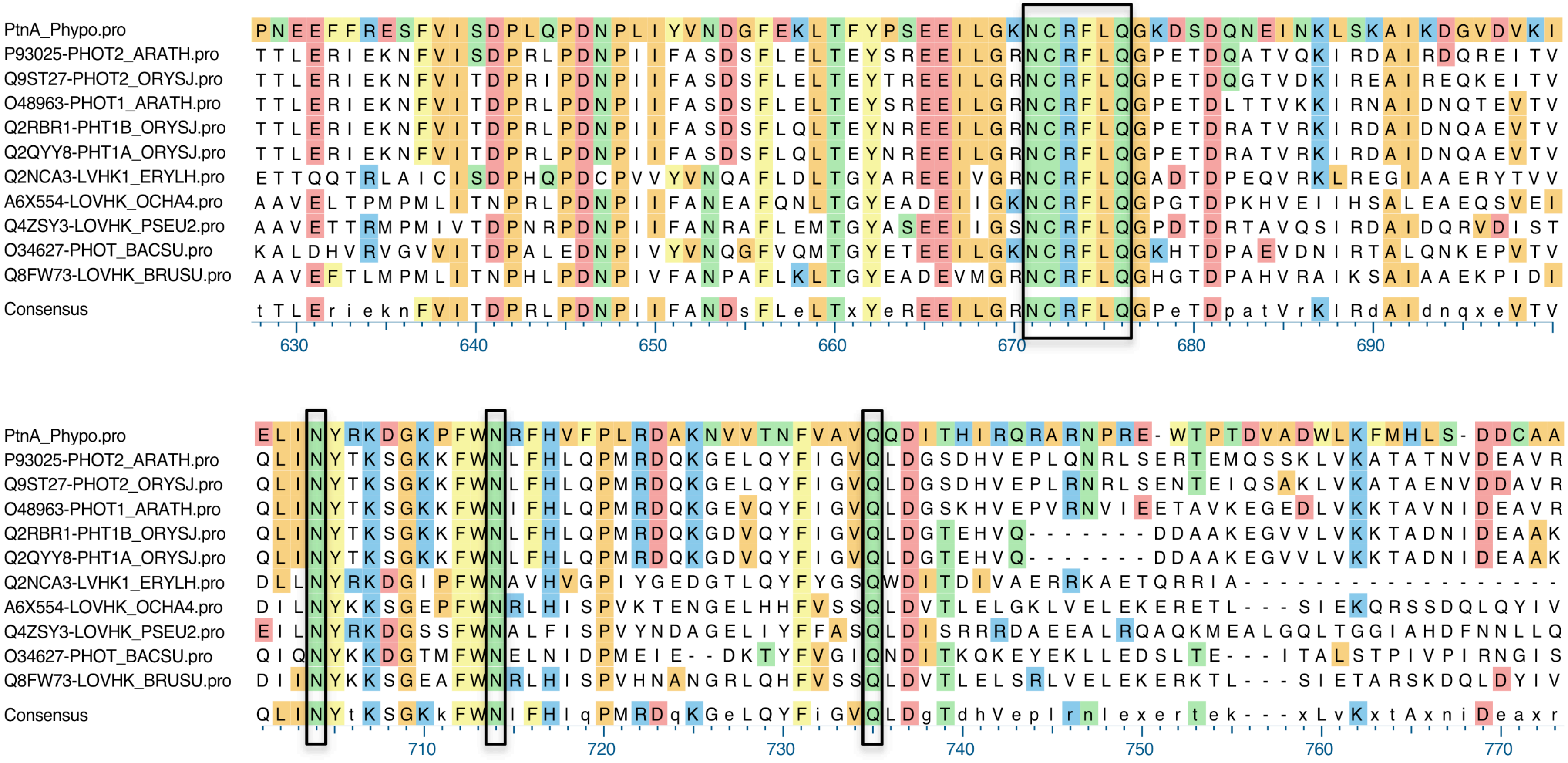

Figure S18. Putative *P. polycephalum* LOV domain protein LovA (PtnA_Phypo) chromophore binding region aligned with phototropins and their orthologs from *Arabidopsis thaliana*, *Oryza sativa subsp. japonica*, *Erythrobacter litoralis*, *Ochrobactrum anthropi*, *Pseudomonas syringae*, *Bacillus subtilis*, *Brucella suis*. Conserved amino acid residues involved in chromophore binding of phototropins (Heintzen 2012) are boxed.

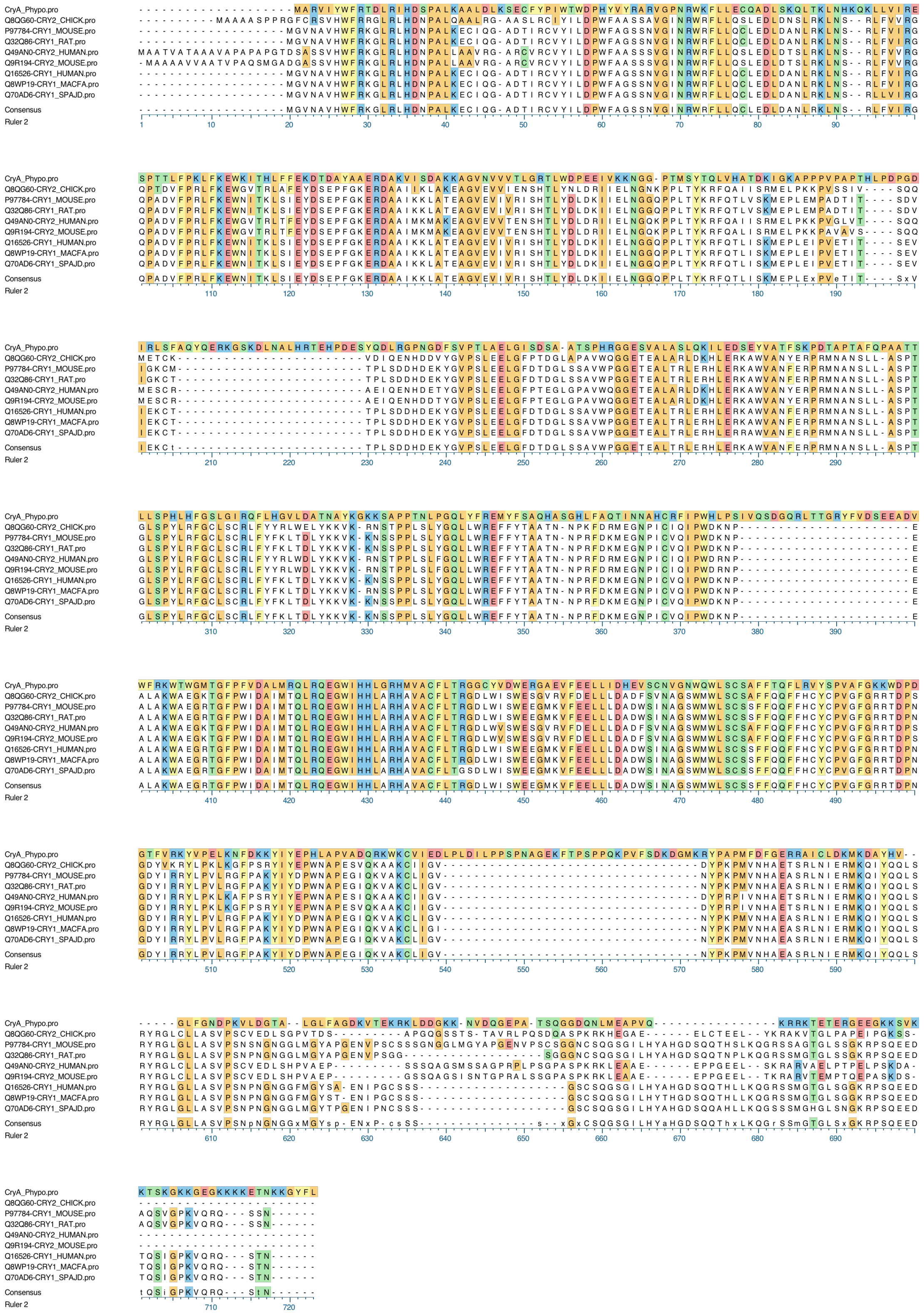

Figure S19. Predicted sequence of *P. polycephalum* cryptochrome CryA aligned with cryptochrome sequences of the vertebrate orthologs of *Gallus gallus*, *Mus musculus*, *Rattus norvegicus*, *Homo sapiens*, *Macaca fascicularis*, *Spalax judaei*.

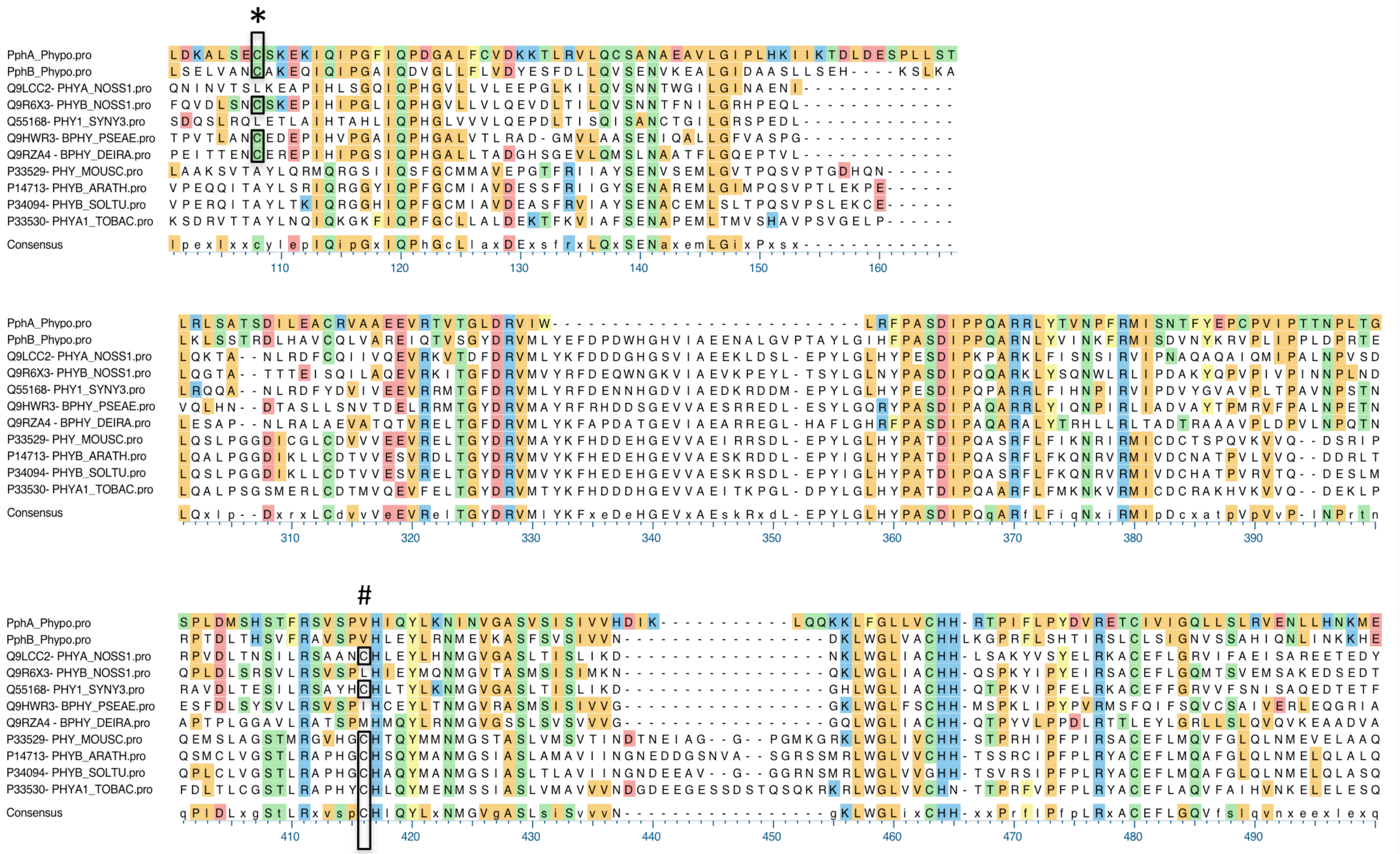

Figure S20. Alignment of the predicted *P. polycephalum* phytochromes PhyA (PphA_Phypo) and PhyB (PphB_Phypo) sequences with bacterial and plant orthologs of *Nostoc sp.*, *Synechocystis sp.*, *Pseudomonas aeruginosa*, *Deinococcus radiodurans*, *Mougeotia scalaris*, *Arabidopsis thaliana*, *Solanum tuberosum*, *Nicotiana tabacum*. Chromophore binding cysteines in bacterial and plant phytochromes are boxed (*,#). Amino acid positions refer to *Arabidopsis thaliana* phytochrome B (PHYB).

14. DYW-type PPR Proteins

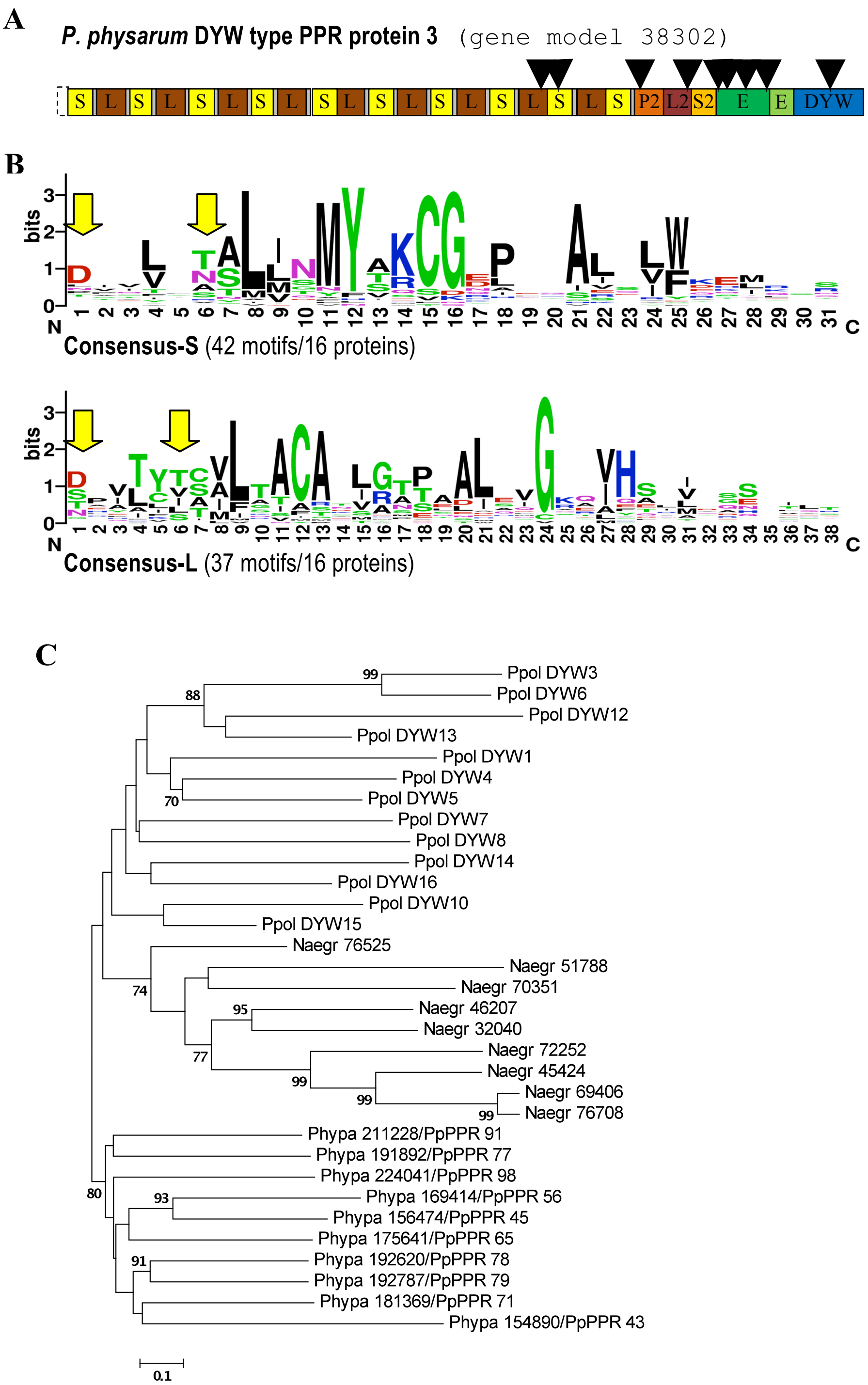

Figure S21. DYW-type PPR proteins in *P. polycephalum*. (A) Model structure of the longest of 16 DYW-type pentatricopeptide repeat (PPR) proteins (Gene ID: 38302) encoded in the genome of *Physarum polycephalum*. As in the land plant homologues, the *Physarum* proteins terminate with a C-terminal DYW domain with cytidine deaminase similarity, preceded by conserved E and E+ domains and the characteristic P2, L2 and S2-type PPR

repeats. In contrast to the plant homologues, however, only L ("long") and S-type ("short") but no classic P-type PPRs motif alternate in the upstream PPR array that is likely responsible for recognizing specific RNA targets. In additional contrast to their largely intron-free plant counterparts, the *Physarum* PPR protein genes are characterized by numerous intron insertions (black arrows). (B) Weblogo profiles (http://weblogo.berkeley.edu) for 42 L-type (top) and 37 S-type (bottom) PPR repeats in the 16 DYW proteins identified in *P. polycephalum*. The yellow arrows highlight amino acid positions 1 and 6 known to be crucial for RNA nucleotide recognition in the plant homologues. (C)Phylogenetic analyses of DYW-type PPR proteins in *Physarum polycephalum* (Ppol), *Naegleria gruberi* (Naegr) and the moss *Physcomitrella patens* (Phypa) show no evidence for evolutionary recent horizontal gene transfer but cluster taxon-wise. The phylogenetic tree shown is a Neighbor-Joining tree using Poisson-corrected distances with node support from 10,000 bootstrap replicates indicated where at least 70%. No differences in topology are found in Maximum Likelihood analyses for confidently supported nodes. Three particularly short DYW-type PPR coding sequences (DYW2, 9 and 11) were excluded from phylogenetic analysis.

15. Phylogenetic Position of *P. polycephalum* Within the Eukaryotes

Thirty highly conserved proteins (Table S8) were aligned using muscle with default parameters. Using Scafos (Roure, et al. 2007), the single protein alignments were concatenated. We identified the best matrix for the tree calculation using the test program implemented in Mega5 (Tamura, et al. 2011). The final maximum likelihood tree was calculated with the JTT matrix and partial deletion. Further calculation with an alignment purified with GBlocks (Talavera and Castresana 2007) using the LGF matrix yielded the same tree topology indicating the validity of the tree (Fig. S22). *Naegleria gruberi* as a representative of the Excavata is distantly related to Plants, Animals, Fungi, and Amoebozoa and thus was used as an outgroup. E. histolytica is not correctly placed in this tree, since it has an accellerated rate of evolution probably due to its endoparasitic life style.

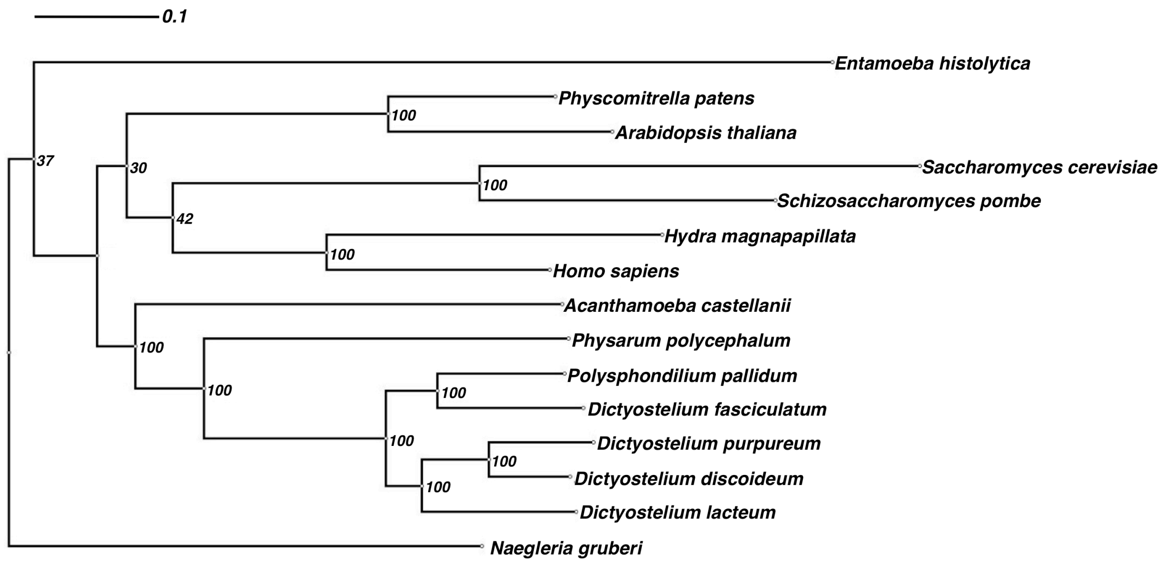

Figure S22. Alignment from a concatenated data set of 30 highly conserved proteins. The tree was rooted with *Naegleria gruberi*. The *Dictyostelium* lacteum genome manuscript is in preparation (Glöckner *et al.*, unpublished results). The subtree with only the Amoebozoa (excluding *Entamoeba*) was used for Figure 1B of the main manuscript.

Table S8. Set of *D. discoideum* genes highly conserved among the eukaryotes used for calculation of the phylogenetic tree shown in Fig. S22. Sequence ID numbers and gene names refer to the corresponding entries in the dictyBase (http://dictybase.org).

Sequence ID	Gene	Protein
DDB0185047	*hspE-1*	heat shock protein, heat shock cognate protein Hsc70-2
DDB0185063	*rps4*	40S ribosomal protein S4
DDB0185123	*catA*	catalase
DDB0185150	*gskA*	glycogen synthase kinase 3, protein serine/threonine kinase, protein kinase, CMGC group, GSK family protein kinase TBC domain protein
DDB0190714		TBC domain protein
DDB0191154	*cdcD*	cell division cycle protein 48, CDC48 family AAA ATPase
DDB0201646	*pyr1-3*	dihydroorotase, aspartate carbamoyltransferase, glutamine-dependent carbamoyl-phosphate synthase
DDB0205772	*devr1*	Developmentally regulated GTP-binding protein 2 (DRG 2).
DDB0215353	*eif2G*	translation initiation factor 2 gamma
DDB0215390	*dymB*	dynamin B
DDB0215391	*rps2*	ribosomal protein S2
DDB0216174	*kif3*	kinesin family member 3, kinesin-1
DDB0220638		citrate synthase, mitochondrial
DDB0229908	*aco1*	putative iron regulatory protein, aconitate hydratase, aconitase
DDB0230056	*rps14*	40S ribosomal protein S14
DDB0230193	*pdhA*	pyruvate dehydrogenase E1 alpha subunit
DDB0231242	*rpl5*	60S ribosomal protein L5
DDB0231248	*thrS1*	threonyl-tRNA synthetase, threonine-tRNA ligase
DDB0231269	*valS1*	valyl tRNA synthetase, valine-tRNA ligase
DDB0231288	*idhA*	isocitrate dehydrogenase (NAD+), isocitrate dehydrogenase (NAD+) alpha subunit
DDB0231317	*proS*	prolyl-tRNA synthetase, proline-tRNA ligase
DDB0231358	*scsB*	succinate-CoA ligase (GDP-forming), succinyl-CoA synthetase (GDP-forming), GTP-specific succinyl-CoA synthetase beta subunit
DDB0231400	*fumH*	fumarate hydratase, fumarase
DDB0231421	*pyk*	pyruvate kinase, phosphoenolpyruvate kinase
DDB0231665	*mpgA*	mannose-1-phosphate guanylyltransferase, GTP:alpha-D-mannose-1-phosphate guanylyltransferase
DDB0233131	*ascc3l*	DEAD/DEAH box helicase, U5 small nuclear ribonucleoprotein 200 kDa helicase, Sec63 domain-containing protein
DDB0233663		heat shock protein Hsp70 family protein
DDB0233910	*eIF3s2*	WD40 repeat-containing protein, eIF-3 beta, eukaryotic translation initiation factor 3 (eIF3) subunit 2
DDB0234259	*eIF5b*	eukaryotic translation initiation factor 5B
DDB0237576	*nol5a*	NOP5 family protein

16. Appendix A: Origin of LU352 and Other Strains Used in this Study

This chapter provides comprehensive information on the origin of strains employed for genome (strain LU352; Table S10, Fig. S23) and for transcriptome sequencing (Table S9). The overview is helpful to retrace the occurrence of natural single nucleotide polymorphisms (SNPs) between the natural isolates and the laboratory strains derived thereof. These SNPs provide a highly valuable ressource for the mapping of genes and for Mendelian analyses of mutated genes. Information on the genotype given for each strain is helpful for the design of genetic experiments.

The strains used in this study have been derived from three natural isolates of diploid plasmodial strains, Wisconsin 1 (Wis 1), Wisconsin 2 (Wis 2), and Indiana. LU352, LU897, and LU898 are highly isogenic with the Colonia Leicester strain CL (Tables S10, S11, Figs S23, S24).

The haploid amoebal strain LU352 (Dee, et al. 1989a) which was employed for genome sequencing is notionally 99% isogenic with the CL strain (see Table S10; Fig. S23). CL was derived by (Cooke and Dee 1975) and has become the reference strain for genetic analyses in *P. polycephalum*, on the basis that it shows the ability to develop into plasmodia either apogamically (Anderson, et al. 1976) or by heterothallic mating. CL was derived from spores of a plasmodium (C50) that was itself derived from spores of the "Colonia isolate" (Wheals 1970). In order to facilitate genetic analyses, it was necessary to create additional amoebal strains that were highly isogenic with CL, but were of different mating types, and carried other useful markers. Two programmes of inbreeding were carried out to introduce mating type and plasmodial fusion alleles from the heterothallic Wis 1 and Indiana isolates into the CL genetic background (Adler and Holt 1974; Cooke and Dee 1975). Inbreeding was carried out by repeatedly backcrossing heterothallic progeny in successive generations to CL (or, in the Wis 1 programme to CLd, a spontaneous mutant of CL in which apogamic development was less efficient, thus favouring plasmodium formation by mating). It emerged during these inbreeding programmes that where natural polymorphisms were identified, CL carried an allele that also occurred in Wis 1, and it has been suggested that the Colonia isolate may not be a natural isolate, but may instead have been derived in some way from Wis 1 (Honey, et al. 1979; Kawano, et al. 1987b). One of the parents of LU352 is LU213 (Dee, et al. 1989a), which is ultimately derived from both inbreeding programmes mentioned above, and carries the *matA3* allele from the Indiana isolate. The other parent is CLd-AXE (McCullough, et al. 1978), from which LU352 inherits at least one gene allowing it to proliferate in liquid, axenic medium (Dee, et al. 1989a), in contrast to other laboratory strains, which must be maintained in monoxenic culture, typically with *Escherichia coli* as the food source. CLd-AXE is a spontaneous mutant of CLd, derived by repeatedly selecting for the ability to proliferate under axenic conditions in liquid medium (Dee, et al. 1989a; McCullough, et al. 1978).

Table S9. Amoebal parents of plasmodial strains used for transcriptome sequencing. The plasmodial strains employed for transcriptome sequencing as listed in Table S1 were obtained by crossing or by apogamic development of amoebal strains. The genotype of each corresponding amoebal strain and its origin (its plasmodial or amoebal parent strain) from which it was derived is displayed.

**Strain**	**Reference**	**Known or deduced genotype**	**Derived as**	**Origin**	**Ploidity of origin**
AI35	(Moriyama and Kawano 2003)	*matA2 matB1 gadA*	Progeny clone	a x i	2n
DP246	(Moriyama and Kawano 2003)	*matA16 matB13*	Progeny clone	DP89 x DP90	2n
DP89	(Kirouac-Brunet, et al. 1981)	*matA15 matB12*	Progeny clone	Wis-2	2n
DP90	(Kirouac-Brunet, et al. 1981)	*matA16 matB13*	Progeny clone	Wis-2	2n
LU897	(Anderson, et al. 1989)	*matA1 matB1 matC1 fusA2 fusB1 fusC1 whiA1*	Progeny clone	LU853 x LU887	2n
LU898	(Kawano, et al. 1987a)	*matA2 matB1 matC1 fusA2 fusB1 fusC1 whiA1 leuA1*	Progeny clone	LU853 x LU887	2n
MA275	(Kirouac-Brunet, et al. 1981; Schedl, et al. 1984)	*matA15* or *matA16 gadA*$^{+}$ *npf*$^{+}$	Progeny clone	Wis-2	2n
PHO1	(Starostzik and Marwan 1998)	*matA2 fusA1 fusB1 fusC1 gadAh whiA*$^{+}$	Sporulation mutant	WT31	1n
PHO26	(Starostzik and Marwan 1998)	*matA2 fusA1 fusB1 fusC1 gadAh whiA*$^{+}$	Sporulation mutant	WT31	1n
PHO3	(Sujatha, et al. 2005)	*matA2 fusA1 fusB1 fusC1 gadAh whiA*$^{+}$	Sporulation mutant	WT31	1n
WT31	(Starostzik and Marwan 1998)	*matA2 fusA1 fusB1 fusC1 gadAh whiA*$^{+}$	Progeny clone	LU352 x LU897	2n

Table S10. Genotype and origin of *P. polycephalum* progenitor strains of LU352

Strain	Reference	Known or deduced genotype	Origin	Notional % isogenic with CL
a	(Dee 1960)	*matA1 fusA1 fusB1 fusC1*	Wisconsin 1 isolate spores	0
B173	(Dee 1966)	*matA3 matB3 matC2 fusA4 fusC2*	Indiana isolate B17 spores	0
C50	(Wheals 1970)	*matA2 gadAh matB1 matC1 fusA2 fusB1 fusC1*	Progeny of 'Colonia isolate'	~100
CH17	Adler, unpublished	*matA3 matB3 fusC2*	B173 x CL	50
CH20	(Adler and Holt 1974)	*matA3 matB3 fusC2*	CH17 x CL	75
CH21	(Adler and Holt 1975)	*matA3 matB3 fusC2*	CH20 x CL	88
CH36	(Adler and Holt 1974)	*matA2 gadAh matB1 matC1 fusA2 fusB1 fusC1 emeE4*	EMS mutagenesis of CL	~100
CH107	(Adler and Holt 1975)	*matA3 matB3 matC2 fusC2 emeE4*	CH21 x CH36	94
CH121	Adler, unpublished	*matA2 gadAh matB1 matC1 fusA2 fusB1 fusC1*	EMS mutagenesis of CL	~100
CH188	(Anderson and Dee 1977)	*matA3 matB3 fusA2 fusB1 fusC2 emeE4*	CH107 x CH121	97
CL	(Cooke and Dee 1975)	*matA2 gadAh matB1 matC1 fusA2 fusB1 fusC1*	Progeny of selfed C50	-
CL5001d	(Cooke and Dee 1975)	*matA2 gadAh* $npfC^{CLd}$ *matB1 matC1 fusA2 fusB1 fusC1 leuA1*	NMG mutagenesis of CLd	~100
CLd	(Cooke and Dee 1974)	*matA2 gadAh* $npfC^{CLd}$ *matB1 matC1 fusA2 fusB1 fusC1*	Spontaneous mutant of CL	~100
CLd-AXE	(McCullough, et al. 1978)	*matA2 gadAh* $npfC^{CLd}$ *matB1 matC1 fusA2 fusB1 fusC1 axe*	CLd 'adapted' to AXE medium	~100
E27	(Gingold, et al. 1976)	*matA2 gadAh* $npfC^{CLd}$ *matB1 matC1 fusA2 fusB1 fusC1 hts-1*	NMG mutagenesis of CLd	~100
LU173	(Cooke and Dee 1975)	*matA1 fusA1 fusB1 fusC1*	*a* x CLd	50
LU203	(Dee 1978)	*matA2 gadAh* $npfC^{CLd}$ *matB1 matC1 fusA2 fusB1 fusC1 hts-1 leuA1*	E27 x LU860	99
LU213	(Dee, et al. 1989a)	*matA3 matB3 fusA1 fusB1 fusC1 leuA1*	LU203 x LU862	98
LU352	(Dee, et al. 1989a)	*matA2 gadAh* $npfC^{CLd}$ *matB3 fusA1 fusB1 fusC1 axe*	CLd-AXE x LU213	99
LU523	(Cooke and Dee 1975)	*matA1 fusA1 fusB1 fusC1*	LU173 x CLd	75
LU574	(Cooke and Dee 1975)	*matA1 fusA1 fusB1 fusC1*	LU523 x CLd	88
LU624	(Cooke and Dee 1975)	*matA1 fusA1 fusB1 fusC1*	LU574 x CLd	94
LU640	(Dee 1978)	*matA2 gadAh* $npfC^{CLd}$ *fusA1*	LU624 x CLd	97
LU648	(Cooke and Dee 1975)	*matA1 matB1 matC1 fusA1 fusB1 fusC1*	LU624 x CLd	97
LU860	(Dee 1978)	*matA1 matB1 matC1 fusA1 fusB1 fusC1 leuA1*	LU648 x CL5001d	98
LU862	(Anderson and Dee 1977)	*matA3 matB3 fusA1 fusB1 fusC1 emeE4*	CH188 x LU640	97

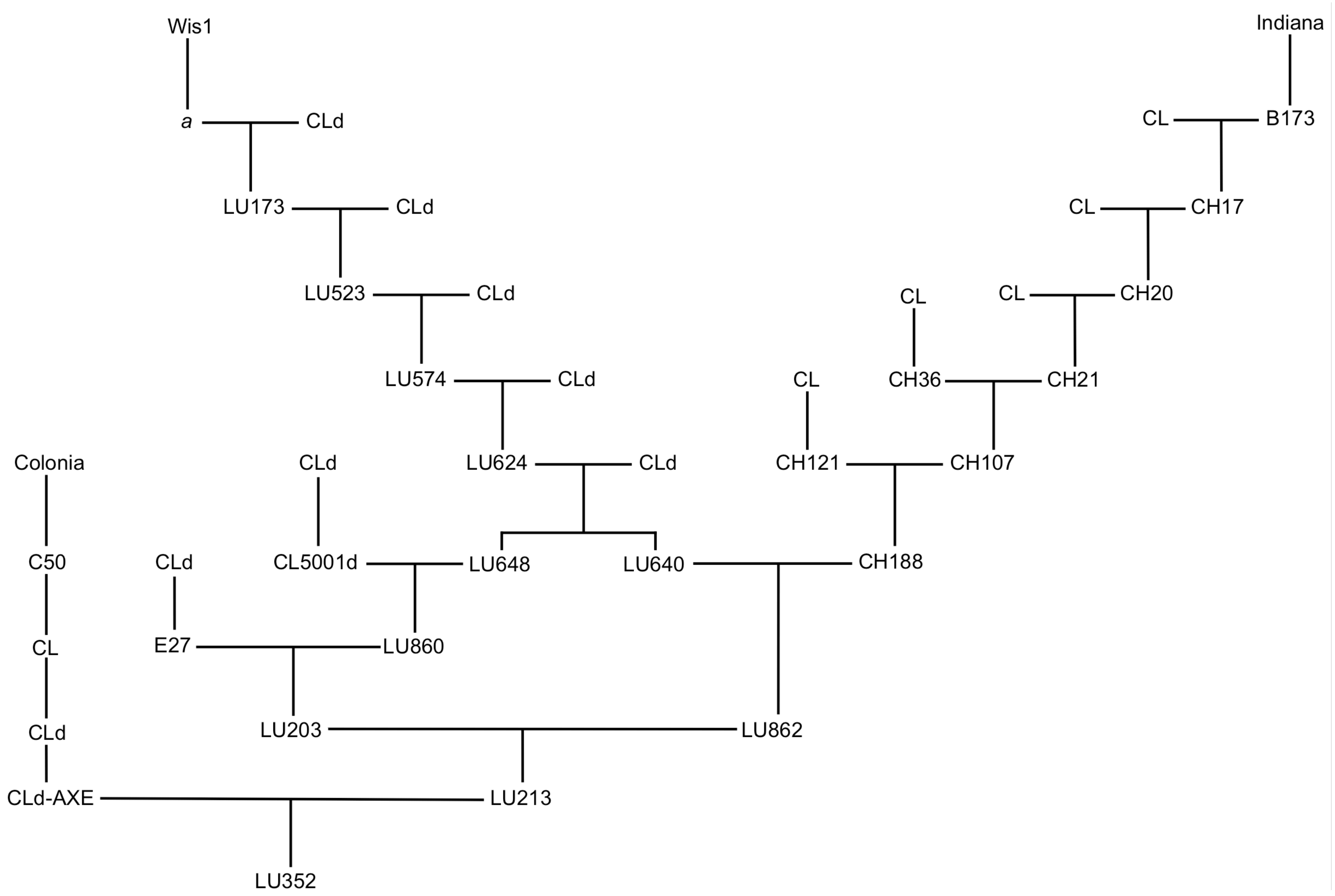

Figure S23. Pedigree of *P. polycephalum* strain LU352

Table S11. Genotype and origin of *P. polycephalum* progenitor strains of the albino strains LU897 and LU898

Strain	Reference	Known or deduced genotype	Origin	Notional % isogenic with CL
a	(Dee 1960)	*matA1 matB1 matC?* fusA1 fusB1 fusC1 saxA1*	Wisconsin 1 isolate spores	0
ai:36	(Cooke and Dee 1975)	*matA2 fusC1*	*a* x *i*	0
C50	(Wheals 1970)	*matA2 gadAh matB1 matC1 fusA2 fusB1 fusC1*	Progeny of 'Colonia isolate'	~100
CL	(Cooke and Dee 1975)	*matA2 gadAh matB1 matC1 fusA2 fusB1 fusC1*	Progeny of selfed C50	-
CL5001d	(Cooke and Dee 1975)	*matA2 gadAh* $npfC^{CLd}$ *matB1 matC1 fusA2 fusB1 fusC1 leuA1*	NMG mutagenesis of CLd	~100
CL6082	(Anderson and Dee 1977)	*matA2 gadAh npfB2 matB1 matC1 fusA2 fusB1 fusC1*	NMG mutagenesis of CL	~100
CLd	(Cooke and Dee 1974)	*matA2 gadAh* $npfC^{CLd}$ *matB1 matC1 fusA2 fusB1 fusC1*	Spontaneous mutant of CL	~100
i	(Dee 1960)	*matA2 matB4 matC?* fusA2 fusB2 fusC1*	Wisconsin 1 isolate spores	0
LU1	(Cooke and Dee 1975)	*matA2 fusA1 fusB1 fusC1 saxA1*	*ai*:36 x *a*	0
LU173	(Cooke and Dee 1975)	*matA1 fusA1 fusB1 fusC1*	*a* x CLd	50
LU418	(Cooke and Dee 1975)	*matA2 fusA1 fusB1 fusC1*	LU1 x CLd	50
LU523	(Cooke and Dee 1975)	*matA1 fusA1 fusB1 fusC1*	LU173 x CLd	75
LU574	(Cooke and Dee 1975)	*matA1 fusA1 fusB1 fusC1*	LU523 x CLd	88
LU586	(Cooke and Dee 1975)	*matA2 fusA1 fusB1 fusC1*	LU418 x LU523	63
LU599	(Cooke and Dee 1975)	*matA2 fusA1 fusB1 fusC1*	LU574 x LU586	75
LU624	(Cooke and Dee 1975)	*matA1 fusA1 fusB1 fusC1*	LU574 x CLd	94
LU635	(Cooke and Dee 1975)	*matA2 fusA1 fusB1 fusC1*	LU599 x LU624	84
LU648	(Cooke and Dee 1975)	*matA1 matB1 matC1 fusA1 fusB1 fusC1*	LU624 x CLd	97
LU677	(Cooke and Dee 1975)	*matA2 fusA1 fusB1 fusC1*	LU635 x LU648	91
LU688	(Cooke and Dee 1975)	*matA2 matB1 matC1 fusA1 fusB1 fusC1*	LU648 x LU677	94
LU853	(Cooke 1974)	*matA1 matB1 matC1 fusA2 fusB1 fusC1 leuA1*	LU648 x CL5001d	98
LU887	(Anderson 1977)	*matA2 matB1 matC1 fusA1 fusB1 fusC1 whiA1*	LU688**	94
LU897	(Anderson, et al. 1989)	*matA1 matB1 matC1 fusA2 fusB1 fusC1 whiA1*	LU853 x LU887	96
LU898	(Kawano, et al. 1987a)	*matA2 matB1 matC1 fusA2 fusB1 fusC1 whiA1 leuA1*	LU853 x LU887	96

**matC* alleles of *a* and *i* unknown but *matC1* and *matC3* segregated among the progeny of *a* x *i* (Kawano, et al. 1987b)

**selfed in a mixture with CL6082

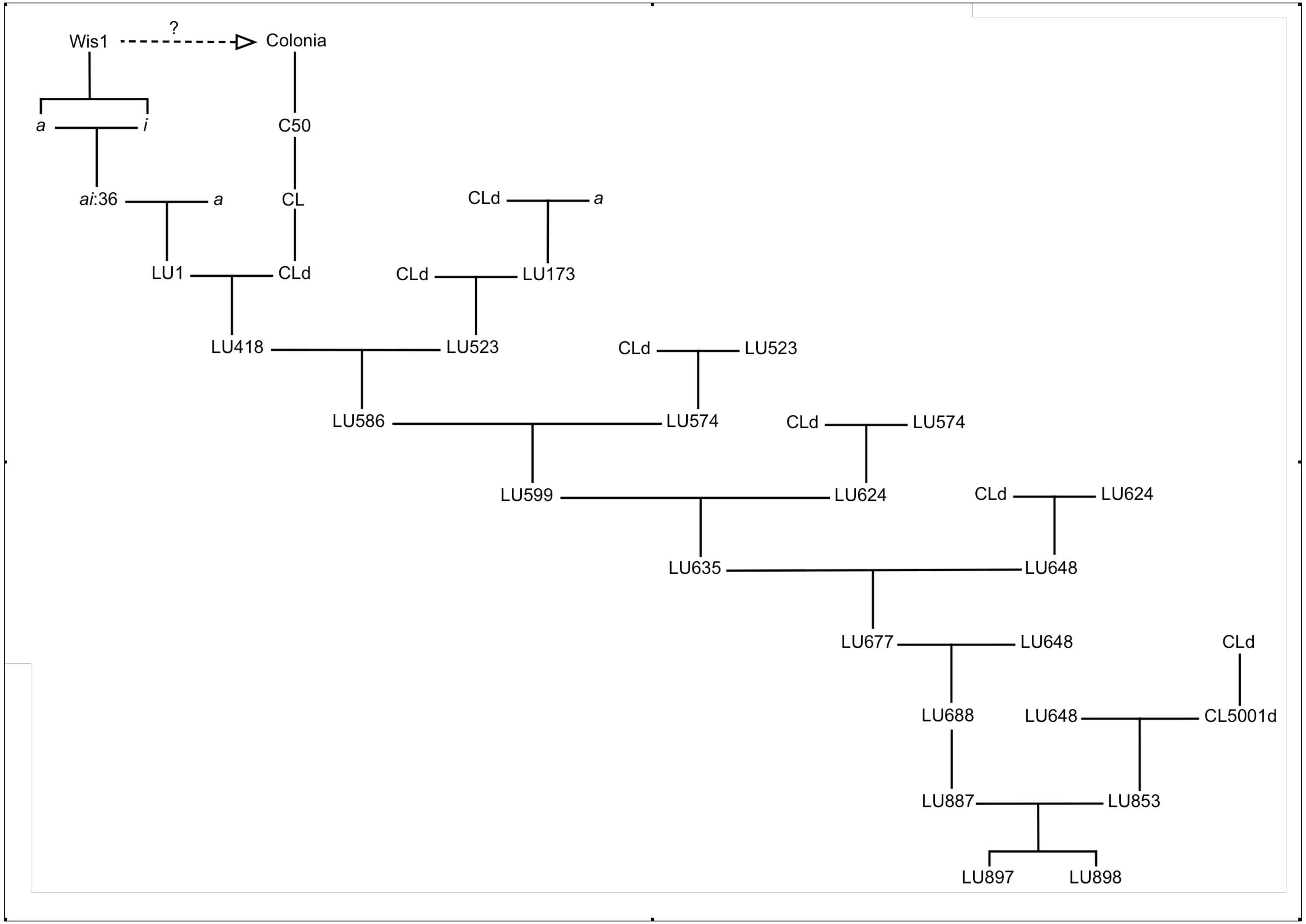

Figure S24. Pedigree of *P. polycephalum* albino strains LU897 and LU898

17. Appendix B: Rules for *P. polycephalum* Genetic Nomenclature

Reprinted from *Physarum* Newsletter PNL 27:1 (1995) with kind permission of the authors

Genetic Nomenclature for *Physarum* II

Timothy G. Burland[1] & Dominick Pallotta[2]

[1]McArdle Laboratory, 1400 University Avenue, Madison, WI 53706, USA

[2]Department of Biology, Laval University, Ste Foy, Quebec, Canada GIK 74P

Why make rules for genetic nomenclature?

Strict adherence to rules of nomenclature for genes and their products is essential for precision and clarity in discussion of gene action, both within a single piece of work and when comparing multiple pieces of work from diverse sources. For any particular organism, it is essential that only one set of rules be applied. Ideally, the same rules would apply to all organisms, but there are unfortunately a variety of different rules for different species. Even the rules of genetic nomenclature for budding yeast and fission yeast differ significantly.

Who makes the rules?

Proposals for genetic nomenclature rules for *Physarum polycephalum* were last updated in 1986 (Anderson, Burland & Dee., *Physarum* Newsletter **18**(2), 2-5). As far as we know, no objections to these proposals were raised, either with the original authors, or through the pages of *Physarum* Newsletter, or at *Physarum* workshops. We infer that there are no strong objections, and propose that these core rules be maintained. Since myxomycetes are quite similar to one another and since homologs and physiological systems are likely to be broadly conserved, we suggest these rules be adopted by those working with other myxomycetes, such as other species of *Physarum* and related genera such as *Didymium*.

What happens when the rules are not followed?

Since 1986, several *Physarum* genes described in the literature have been named using different nomenclature systems. These include Eα-Tu, *H41, H42,* Nα-Tu, P46, php, POL 3, Pprap1, Ppras1, Ppras2 and SSU, names that are consistent with neither the 1986 rules nor one-another. This can lead to confusion between locus names and alleles, between genes and products, and between similar genes isolated by different groups. Inconsistent nomenclature also looks bad to readers from outside the field – it's bad public relations. We hope that authors will consider renaming genes that don't comply to conform to the 1986 rules.

Why make more rules?

Due to progress in molecular genetic analysis of *Physarum*, extension of the genetic nomenclature rules is desirable. Our recent work necessitated introduction of specific elements of nomenclature not covered by the 1986 rules. We propose these here formally as additional rules and invite comments. We present the 1986 rules as a reminder.

THE 1986 RULES

1. Each gene should be designated by an italicized symbol comprising three lower-case letters followed by one uppercase letter.

For example, a gene controlling leucine biosynthesis would be designated *leuA* (and not *leu A, leu-A, leu*A, *LeuA*...)

2. Different genes that mutate to produce similar phenotypes should share the same tree-letter lower-case symbol but should be distinguished by different uppercase letters.

For example, different genes controlling leucine biosynthesis would be designated *leuA, leuB, leuC,* etc. Ras genes might be designated *rasA, rasB, rasC,* etc.

3. A mutant allele should be designated by placing an italicized number after the gene symbol. If it is not known in which of several genes the mutation has occurred, a hyphen should be used in place of the upper-case letter.

For example, when mutations affecting leucine biosynthesis are first isolated and their loci are unknown, they are designated *leu-1, leu-2, leu-3 .. leu-99.* If then *leu-1 and leu-3* map to the same locus, while *leu-2* maps to a second locus and *leu-99* maps to a third, those alleles would then be designated *leuA1, leuA3, leuB2,* and *leuC99.*

4. Wild-type alleles should be designated by the gene symbol followed by a superscript „plus" sign. For genes that show natural (including RFLP) polymorphism, alternate alleles should be represented by italicized numbers after the gene symbol.

For example, the wild-type allele of *leuA* would be designated *leuA*$^{+}$. However, since mating type loci show natural polymorphism, the different alleles are distinguished with a number, just as mutant alleles are, hence *matA1, matA2, matA3 ... matA16.* Similarly, the RFLP allele of *betC*$^{+}$ from CL is denoted *betC1* while the corresponding RFLP allele from MA275 is *betC2.*

5. Phenotypic traits should be described in words or by the use of the first three letters of the gene symbol, but without italics and with the first letter in upper case. A superscript „plus" sign should indicate the wild-type phenotype.

For example, a mutant that requires leucine as a result of a *leuA1* mutation would be designated Leu, while a strain that does not carry any *leu* mutations would be designated Leu^{+}.

6. Each amoebal strain should be given a two-letter upper-case code followed by a number. For newly-isolated mutants, a three-letter, upper-case code may precede the number. Strain names should not be italicized.

For example, LU648 and LU688 are strains constructed at Leicester University, MA275 and MA389 are strains constructed at the McArdle Laboratory, and DP72 is a strain constructed at Dominick Pallotta's laboratory. BEN41 and BEN210 are benzimidazole-resistant mutants.

7. Plasmodia should be named after the amoebae where they were derived. Crossed plasmodia are designated by the names of the two amoebal strains involved separated by a multiplication sign. Heterokaryotic plasmodia are designated by the names of the plasmodial strains involved separated by a plus sign.

For example, a plasmodium developing apogamically from CL amoebae is designated CL. A plasmodium resulting from a cross of LU648 with LU688 amoebae is designated LU648xLU688.

A heterokaryotic plasmodium resulting from fusion of a CL plasmodium with a BEN210 plasmodium would be designated CL+BEN210. A heterokaryon resulting from fusion of LU648xLU688 and LU352 plasmodia would be designated LU648xLU688+LU352.

PROPOSED ADDITIONS TO THE 1986 RULES

8. Amoebae constructed by fusion of two different strains of amoebae should be designated by the names of the two amoebal strains involved separated by a multiplication sign.

For example, a diploid amoebal strain obtained by abortive crossing of MA316 and BEN210 haploid amobae would be designated MA316xBEN210. This is the same nomenclature used for crossed plasmodia, as a selfed plasmodium arising from such a diploid amoebal strain would usually behave as if it were formed by a true sexual cross, if one could be performed.

9. A promoter should be designated by an italicized uppercase letter „P“ followed by the symbol for the gene whose transcription it promotes. Where a promoter drives expression of a specific gene, the promoter and gene symbols should be separated by a hyphen.

For example, the promoter of the *ardC* actin gene would be designated *PardC,* while the promoter of the *ardB* actin gene would be designated *PardB.* A construct where *PardC* is driving expression of the *leuA* gene would be designatd *PardC-leuA.*

When a *Physarum* promoter drives expression of a gene from another organism, the nomenclature for the other organism's gene is not changed, as genetic nomenclature is specific for the organism of origin. Thus when *PardC* drives expression of *hph*, the construct is called *PardC-HPH,* and not *PardC-hphA,* because *hph* is the accepted name of this bacterial gene. If *PardC* drives expression of the budding yeast wild-type *LEU1* gene, the construct would be designated *PardC-LEU1,* because wild-type alleles for budding yeast genes are capitalized.

10. A transcriptional terminator should be designated by an italicized uppercase letter „T“ followed by the symbol for the gene whose transcription it terminates.

For example, the terminator of the *ardC* actin gene would be designated *TardC,* while the terminator of the *ardB* actin gene would be designated *TardB.*

11. An enhancer element should be designated by an italicized uppercase letter „E“ followed by the symbol for the gene whose transcription it enhances.

For example, an enhancer of the *ardC* actin gene would be designated *EardC,* while an *ardB* enhancer would be designated *EardB.* If multiple enhancers are discovered for the same gene, an italicized number should follow the italicized upper-case E, for example *E1ardC, E2ardC,* etc.

12. Deletion of part or all of a specific gene should be designated by placing an upper-case italicized Greek „*delta*“ and an italicized number after the usual gene symbol.

For example, a deletion in the *ardD* actin gene would be designated *ardDΔ1*, and a different deletion in the same gene would be designated *ardDΔ2.*

13. An insertion in a specific gene should be designated by placing an upper-case italicized Greek „*sigma*“ and an italicized number or symbol after the usual gene symbol.

For example, an insertion of a piece of DNA into the *ardD* actin gene would be designated *ardDΣ1*, a different insertion in *ardD* would be designated *ardDΣ2.* If the insertion is another gene or known element, the symbol for the inserted gene may be used instead of the number. For example, insertion of the *matA16* allele into *ardD* would be denoted *ardDΣmatA16.*

We rejected the use of the uppercase Greek letters alpha („addition") and iota („insertion") as they are too similar to their Roman counterparts, which could lead to confusion between the gene name and the insertion designation.

14. A transgene should be designated by its usual gene symbol. Where the transgene inserts into a known gene, it should be designated by the transgene and target gene symbols separated by an uppercase italicized Greek „*sigma*".

For example, integration of a transforming *matB3* allele into *leuA* would be designated *matB3ΣleuA*. Integration of a transforming *PardC-hph-ardDΔ1* construct into *leuA* would be designated *PardC-hph-ardDΔ1ΣleuA*. If a transforming *ardDΔ1* allele integrated homologously INTO the *ardD* gene, it would be designated *ardDΔ1ΣardD.* However, so far the homologous integration observed have been replacement rather than addition events, and the replacement of the resident *ardD*$^{+}$ allele by the transforming *ardDΔ1* allele is simply designated *ardDΔ1* because the wild-type sequences are no longer there.

15. A polypeptide product should be designated using the same four letters of the gene symbol, but using Roman rather than italic characters, and the first letter should be capitalized. Products of mutant or polymorphic alleles should also carry the allele numbers, in non-italic form. Where the gene product has already been named, that name may be retained.

For example, the product of the *ardD* gene would be designated *ArdD.* It could also be called actin D, as actins had already been named before *ardD* was discovered. The product of the *ardDΔ1* mutant allele of the *ardD* gene would be designated *ArdDΔ1* or actin DΔ1. The product of the *matA1* allele of the *matA* gene would be MatA1, and the product of *matA2* would be MatA2. The product of the *benD* gene, however, is usually called β1B-tubulin rather than BenD, as the tubulin was already known before the *benD* gene was discovered.

We have not proposed spezial nomenclature for transcripts, whether nascent or mature. The transcript from *ardD* is simply referred to as the *ardD* transcript or *ardD* mRNA- This same convention could be applied where the gene product is RNA rather than protein.

16. Extrachromosomal genes should follow the same nomenclature as chromosomal genes.

The 1986 rules for *Physarum* nomenclature are based on proposals of Demerec *et al.* (Genetics **54,** 61-76, 1966). As Demerece *et al* said, „...loci on plasmids and episomes are not different in kind from loci on the chromosome, and – as part of the total genetic complement of the... cell – should be symbolized according to the unified system of nomenclature."

17. Genetic symbols that conform to agreed nomenclature should not be changed once published.

QUICK REFERENCE TO PHYSARUM NOMENCLATURE:

Amoebal strain...LU648
Phenotype...Wild-type; Leu$^+$, MatA1

Selfing amoebal strains................................CL; ATS23
Selfed plasmodia..CL; ATS23
Fused (heterokaryotic) plasmodium............CL+ATS23

Compatible amoebal strains.........................CLd; LU215
Crossed plasmodium...................................CLdxLU215

Haploid *matA2* amoebal strains..................LU352; MA460
Diploid amoebal strain................................LU352xMA460
Plasmodium from LU352xMA460.............LU352xMA460

Mutant amoebal strain.................................BEN210
Mutant phenotype..BEN; benzimidazole-resistant
Mutant allele before locus is known...........*ben-210*
Mutant allele when locus is known............*benD210*
Gene...*benD*
Deletions...*benDΔ1, benDΔ2...*
Insertions..*benDΣ1, benDΣ2, benDΣleuAΔ54...*
Promoter...*PbenD*
Terminator..*TbenD*
Enhancer..*EbenD; E1benD, E2benD...*
Transgene insertion.....................................*benD210ΣardD*
Wild-type gene product..............................BenD or β1B-tubulin
Mutant gene product...................................BenD210 or β1B-210-tubulin

SUGGESTIONS FOR NAMING GENES

We provide the following for the purpose of illumination rather than dictation.

Genes known only from nucleic acid sequence

For a gene of unknown function, whose sequence is unlike any known gene, the best solution is to choose a name based on the phenotype of the disruptant. Choosing a name based on expression pattern seems easier – such as *„apt"* for genes expressed only during the amoeba-plasmodium transition, or *„dev"* for genes expressed preferentially during development. But there are probably more than 26 genes expressed preferentially during development, and surely more than that expressed preferentially in plasmodia. Thus a name like *„pla"* would be used up on discovery of the 27th gene expressed only in plasmodia (see also the next point).

Expresson in amoebae versus plasmodia

When mutation of a set of genes gives a similar phenotype, it is usual to use the same three-letter lowercase code and go through the alphabet in steps for the fourth, uppercase letter (*genA, genB, genC, etc.*; rule 2). Where two similar genes are found and one appears to be amoeba-specific and the other plasmodium-specific, it is tempting to call one *genA* (amoebal) and the other *genP* (plasmodial). But *genP* might also be expressed in flagellates, or there may be a very low level of expression of *genP* in amoebae, which you discover when you disrupt *genP* and find it is lethal for amoebae. Better to avoid assumptions: even if you are sure there is no expression of *genP* at any developmental or vegetative stage of the life cycle except plasmodia, there might be a second homolog, and you can't call the second one *genP.*

Homologs

When choosing a name for a *Physarum* homolog of a gene known from some other organism, preserving gene names across species boundaries is neither necessary nor desirable. Recall that the budding yeast homolog of the fission yeast cell cycle regulator *cdc2*$^{+}$ is CDC28; this distinct nomenclature has not caused any problems in understanding cell cycle biology. Further, unless the „homolog" from another species is replaced with the *Physarum* gene and the *Physarum* gene performs all the same functions, it's hard to be sure it's a true homolog. Thus, a prudent course for naming *Physarum* homologs is to use a name that conforms to the *Physarum* rules. For example, a *Physarum* homolog of a *ras* gene might be called *rasA,* and a *Physarum* gene for a KANN polymerase 3 – like protein might be called *po1A.*

Mutants

When starting from a phenotype of a mutant that is defective for some function, a three-letter gene symbol that is pronouncable as well as descriptive of the phenotype is preferable. For example, *aft-1 aft-2 etc.* („amoeba-flagellate transition") would be a good way to name mutations that prevent flagellate development; *„nff"* („no flagellate formation") would be inferior because you can't pronounce *„nff"* (notice how well *Drosophila* does with its cute, pronouncable gene names. Further, the *„n"* description might not be appropriate for all alleles – some alleles may be leaky so that some flagellate development might occur; other alleles of *nff* genes might even improve efficency of flagellate development. The *aft* choice makes fewer assumptions.

Acknowledgements: TGB is supported by grant #MCB-94-9405605 from the NSF; DP is supported by NSERC of Canada and FCAR de Québec.

18. References

Adler PN, Holt CE 1974. Genetic analysis in the Colonia strain of *Physarum polycephalum*: heterothallic strains that mate with and are partially isogenic to the Colonia strain. Genetics 78: 1051-1062.

Adler PN, Holt CE 1975. Mating type and the differentiated state in *Physarum polycephalum*. Developmental Biology 43: 240-253.

Altschul SF, et al. 1997. Gapped BLAST and PSI-BLAST: a new generation of protein database search programs. Nucleic Acids Research 25: 3389-3402.

Anderson RW 1977. A plasmodial color mutation in the myxomycete *Physarum polycephalum*. Genetical Research 30: 301-306.

Anderson RW, Cooke DJ, Dee J 1976. Apogamic development of plasmodia in the Myxomycete *Physarum polycephalum*: A cinematographic analysis. Protoplasma 89: 29-40.

Anderson RW, Dee J 1977. Isolation and analysis of amoebal-plasmodial transition mutants in the Myxomycete *Physarum polycephalum*. Genetical Research 29: 21-34.

Anderson RW, Hutchins G, Gray A, Price J, Anderson SE 1989. Regulation of development by the *matA* complex locus in *Physarum polycephalum*. Journal of general microbiology 135: 1347-1359.

Anisimova M, Gascuel O 2006. Approximate likelihood-ratio test for branches: A fast, accurate, and powerful alternative. Systematic biology 55: 539-552.

Aravind L, Ponting CP 1997. The GAF domain: an evolutionary link between diverse phototransducing proteins. Trends in Biochemical Sciences 22: 458-459.

Avesson L, Reimegard J, Wagner EGH, Soderborn F 2012. MicroRNAs in Amoebozoa: Deep sequencing of the small RNA population in the social amoeba *Dictyostelium discoideum* reveals developmentally regulated microRNAs. RNA 18: 1771 - 1782.

Bader S, Kortholt A, Van Haastert PJM 2007. Seven *Dictyostelium discoideum* phosphodiesterases degrade three pools of cAMP and cGMP. Biochem. J. 402: 153-161.

Barrantes I, Leipzig J, Marwan W 2012. A next-generation sequencing approach to study the transcriptomic changes during the differentiation of physarum at the single-cell level. Gene regulation and systems biology 6: 127-137.

Bénard M, Maric C, Pierron G 2007. Low rate of replication fork progression lengthens the replication timing of a locus containing an early firing origin. Nucleic Acids Res. 35: 5763-5774.

Bentley DR, et al. 2008. Accurate whole human genome sequencing using reversible terminator chemistry. Nature 456: 53-59.

Block I, Rabien H, Ivanova K 1998. Involvement of the second messenger cAMP in gravity-signal transduction in *Physarum*. Adv Space Res 21: 1311-1314.

Bockaert J, Pin JP 1999. Molecular tinkering of G protein-coupled receptors: an evolutionary success. The EMBO journal 18: 1723-1729.

Brandon MA, Podgorski GJ 1997. G alpha 3 regulates the cAMP signaling system in *Dictyostelium*. Molecular Biology of the Cell 8: 1677-1685.

Bundschuh R, Altmüller J, Becker C, Nürnberg P, Gott JM 2011. Complete characterization of the edited transcriptome of the mitochondrion of *Physarum polycephalum* using deep sequencing of RNA. Nucleic Acids Research 39: 6044-6055.

Chang JB, Ferrell JE 2013. Mitotic trigger waves and the spatial coordination of the *Xenopus* cell cycle. Nature 500: 603-607.

Chih-Chung C, Chih-Jen L 2011. LIBSVM: a library for support vector machines. ACM Transactions on Intelligent Systems and Technology 2: 1 - 27.

Chirgwin JM, Przybyla AE, McDonald RJ, Rutter WJ 1979. Isolation of biologically active ribonucleic acid from sources enriched in ribonuclease. Biochem. 18: 5294-5299.

Clarke M, et al. 2013. Genome of *Acanthamoeba castellanii* highlights extensive lateral gene transfer and early evolution of tyrosine kinase signaling. Genome Biol 14: R11.

Clutterbuck AJ 1970. Synchronous nuclear division and septation in *Aspergillus nidulans*. Journal of general microbiology 60: 133-135.

Conti M, Beavo J 2007. Biochemistry and physiology of cyclic nucleotide phosphodiesterases: essential components in cyclic nucleotide signaling. Ann. Rev. Biochem. 76: 481-511.

Cooke DJ 1974. Studies on the Colonia isolate of *Physarum polycephalum*. [PhD thesis]: PhD thesis, University of Leicester.

Cooke DJ, Dee J 1975. Methods for the isolation and analysis of plasmodial mutants in *Physarum polycephalum* Genetical Research 24: 175-187.

Cooke DJ, Dee J 1974. Plasmodium formation without change in nuclear DNA content in *Physarum polycephalum*. Genetical Research 23: 307-317.

De Souza CPC, Osmani SA 2007. Mitosis, not just open or closed. Eukaryotic cell 6: 1521-1527.

Dee J 1978. A gene unlinked to mating-type affecting crossing between strains of *Physarum polycephalum*. Genetical Research 31: 85-92.

Dee J 1960. A mating-type system in an acellular slime-mould. Nature (London) 185: 780-781.

Dee J 1966. Multiple alleles and other factors affecting plasmodium formation in the true slime mold *Physarum polycephalum* Schw. Journal of Eukaryotic Microbiology 13: 610-616.

Dee J, Foxon JL, Anderson RW 1989a. Growth, development and genetic characteristics of *Physarum polycephalum* amoebae able to grow in liquid axenic medium. J. Gen. Microbiol. 135: 1567-1588.

Dee J, Foxon JL, Anderson RW 1989b. Growth, development and genetic characteristics of *Physarum polycephalum* amoebae able to grow in liquid, axenic medium. J. Gen. Microbiol. 135: 1567-1588.

Dharmawardhane S, Cubitt AB, Clark AM, Firtel RA 1994. Regulatory role of the Ga1 subunit in controlling cellular morphogenesis in *Dictyostelium*. Development 120: 3549-3561.

Eddy SR 2011. Accelerated profile HMM searches. PLoS Computational Biology 7: e1002195.

Edgar RC 2010. Search and clustering orders of magnitude faster than BLAST. Bioinformatics (Oxford, England) 26: 2460-2461.

Eichinger L, et al. 2005. The genome of the social amoeba *Dictyostelium discoideum*. Nature 435: 43-57.

El Yacoubi B, et al. 2006. Discovery of a new prokaryotic type I GTP cyclohydrolase family. J. Biol. Chem. 281: 37586-37593.

Enright AJ, et al. 2003. MicroRNA targets in *Drosophila*. Genome Biology 5.

Essen LO, Mailliet J, Hughes J 2008. The structure of a complete phytochrome sensory module in the Pr ground state. Proc Natl Acad Sci U S A 105: 14709-14714.

Felsenstein J 1985. Confidence limits on phylogenies: An approach using the bootstrap. Evolution 39: 783 - 791.

Finn RD, Clements J, Eddy SR 2011. HMMER web server: interactive sequence similarity searching. Nucleic Acids Research 39: W29-37.

Foe VE, Alberts BM 1983. Studies of nuclear and cytoplasmic behaviour during the five mitotic cycles that precede gastrulation in *Drosophila* embryogenesis. Journal of Cell Science 61: 31-70.

Gingold EC, Grant WD, Wheals AE, Wren M 1976. Temperature-sensitive mutants of the slime mould *Physarum polycephalum*. II. Mutants of the plasmodial phase. Molecular and General Genetics 149: 115-119.

Glöckner G, et al. 2001. The complex repeats of *Dictyostelium discoideum*. Genome Research 11: 585 - 594.

Golderer G, Werner ER, Leitner S, Grobner P, Werner-Felmayer G 2001. Nitric oxide synthase is induced in sporulation of *Physarum polycephalum*. Genes Dev 15: 1299-1309.

Gordon A 2008. FASTX toolkit, Version 0.013.

Gorren AC, Mayer B 2007. Nitric.oxide synthase: a cytochrome P450 family foster child. Biochim. Biophys. Acta 1770: 432-445.

Gouy M, Guindon S, Gascuel O 2010. SeaView version 4: A multiplatform graphical user interface for sequence alignment and phylogenetic tree building. Molecular biology and evolution 27: 221-224.

Grabherr MG, et al. 2011. Full-length transcriptome assembly from RNA-Seq data without a reference genome. Nature Biotechnology 29: 644-652.

Häder D-P, Poff KL 1979a. Inhibition of aggregation by light in the cellular slime mold *Dictyostelium discoideum*. Archives of microbiology 123: 281-285.

Häder D-P, Poff KL 1979b. Light-induced accumulations of *Dictyostelium discoideum* amoebae. Photochemistry and Photobiology 29: 1157-1162.

Häder DP, Schreckenbach T 1984. Phototactic orientation in plasmodia of the acellular slime mold, *Physarum polycephalum*. Plant and Cell Physiology 25: 55-61.

Hanks SK 2003. Genomic analysis of the eukaryotic protein kinase superfamily: a perspective. Genome Biology 4: 111.

Haskins EF 2000. *Stemonitis flavogenita* (*Myxomycetes*) - plasmodial phase (Aphanoplasmodium). Film E: 1-14.

Heath IB 1980. Variant mitoses in lower eukaryotes: indicators of the evolution of mitosis? International review of cytology 64: 1-80.

Heidel A, et al. 2011. Phylogeny-wide analysis of social amoeba genomes highlights ancient origins for complex intercellular communication. Genome Res.: 1882-1891.

Heintzen C 2012. Plant and fungal photopigments. Wiley Interdisciplinary Reviews: Membrane Transport and Signaling 1: 411-432.

Hess PN, De Moares Russo CA 2007. An empirical test of the midpoint rooting method. Biological Journal of the Linnean Society 92: 669-674.

Hofactor I, Fontana W 1994. Fast folding and comparison of RNA secondary structures. Chemical Monthly 125: 167 - 188.

Honey NK, Poulter RTM, Teale DM 1979. Genetic regulation of differentiation in *Physarum polycephalum*. Genetical Research 34: 131-142.

Huang X, Madan A 1999. CAP3: A DNA sequence assembly program. Genome Research 9: 868-877.

Hurley JH 2003. GAF domains: cyclic nucleotides come full circle. Sci STKE 2003: PE1.

Iyer LM, Leipe DD, Koonin EV, Aravind L 2004. Evolutionary history and higher order classification of AAA+ ATPases. J Struct Biol 146: 11-31.

Jékely G 2009. Evolution of phototaxis. Philosophical transactions of the Royal Society of London. Series B, Biological sciences 364: 2795-2808.

Jones DC 2011. Fastq-tools.

Jones P, et al. 2014. InterProScan 5: genome-scale protein function classification. Bioinformatics 30: 1236-1240.

Kawano S, Anderson RW, Nanba T, Kuroiwa T 1987a. Polymorphism and uniparental inheritance of mitochondrial DNA in *Physarum polycephalum*. Journal of general microbiology 133: 3175-3182.

Kawano S, Kuroiwa T, Anderson RW 1987b. A third multiallelic mating-type locus in *Physarum polycephalum*. Journal of general microbiology 133: 2539-2546.

Kirouac-Brunet J, Masson S, Pallotta D 1981. Multiple allelism at the *matB* locus in *Physarum polycephalum*. Canadian Journal of Genetics and Cytology 23: 9-16.

Kopperud R, Krakstad C, Selheim F, Doskeland SO 2003. cAMP effector mechanisms. Novel twists for an 'old' signaling system. FEBS Lett 546: 121-126.

Kubbies M, Pierron G 1983. Mitotic cell cycle control in *Physarum*: Unprecedented insights via flow-cytometry. Experimental Cell Research 149: 57-67.

Kuehn GD 1972. Cell cycle variation in cyclic adenosine 3',5'-monophosphate-dependent inhibition of protein kinase from *Physarum polycephalum*. Biochem Biophys Res Commun 49: 414-419.

Kumagai A, et al. 1989. Regulation and function of Ga protein subunits in *Dictyostelium*. Cell 57: 265-275.

Kwak H, Lis JT 2013. Control of transcriptional elongation. Annu Rev Genet 47: 483-508.

Lamparter T, Marwan W 2001. Spectroscopic detection of a phytochrome-like photoreceptor in the Myxomycete *Physarum polycephalum* and the kinetic mechanism for the photocontrol of sporulation by P_{fr}. Photochem. Photobiol. 73: 697-702.

Leclercq M, Banire Diallo A, Blanchette M 2013. Computational prediction of the localization of microRNAs within their pre-miRNA. Nucl. Acids Res. 41: 7200 - 7211.

Leinonen R, et al. 2011. The European Nucleotide Archive. Nucleic Acids Research 39: D28-31.

Lim WA, Pawson T 2010. Phosphotyrosine signaling: evolving a new cellular communication system. Cell 142: 661-667.

Linder JU, Schultz JE 2003. The class III adenylyl cyclases: multi-purpose signalling modules. Cellular Signalling 15: 1081-1089.

Liu BA, Nash PD 2012. Evolution of SH2 domains and phosphotyrosine signalling networks. Philosophical Transactions of the Royal Society B: Biological Sciences 367: 2556-2573.

Loomis WF 2014. Cell signaling during development of *Dictyostelium*. Developmental Biology 391: 1-16.

MacKenzie AB, Surprenant A, North RA 1999. Functional and molecular diversity of purinergic ion channel receptors. Ann N Y Acad Sci 868: 716-729.

Magrane M, Consortium U 2011. UniProt Knowledgebase: a hub of integrated protein data. Database (Oxford) 2011.

Malik HS, Burke WD, Eickbush TH 1999. The age and evolution of non-LTR retrotransposable elements. Mol. Biol. Evol. 16: 793-805.

Malik HS, Eickbush TH 1999. Modular evolution of the integrase domain in the Ty3/gypsy class of LTR retrotransposons. J. Virol. 73: 5186-5190.

Malik HS, Eickbush TH 2001. Phylogenetic analysis of ribonuclease H domains suggests a late, chimeric origin of LTR retrotransposable elements and retroviruses. Genome Res. 11: 1187-1197.

Marchler-Bauer A, et al. 2015. CDD: NCBI's conserved domain database. Nucleic Acids Research 43.

Maric C, Bénard M, Pierron G 2003. Developmentally-regulated usage of *Physarum* DNA replication origins. EMBO Rep. 4: 474-478.

Marin I, van Egmond WN, van Haastert PJ 2008. The Roco protein family: a functional perspective. FASEB J 22: 3103-3110.

Marwan W, Starostzik C 2002. The sequence of regulatory events in the sporulation control network of *Physarum polycephalum* analysed by time-resolved somatic complementation of mutants. Protist 153: 391-400.

Matveeva NB, Teplov VA, Nezvetskii AR, Orlova TG, Beilina SI 2012. Involvement of cyclic adenosine monophosphate in the control of motile behavior of *Physarum polycephalum* plasmodium. Biofizika 57: 832-839.

McCullough CHR, Dee J, Foxon JL 1978. Genetic factors determining the growth of *Physarum polycephalum* amoebae in axenic medium. Journal of general microbiology 106: 297-306.

Meima ME, Biondi RM, Schaap P 2002. Identification of a novel type of cGMP phosphodiesterase that is defective in the chemotactic *stmF* mutants. Mol. Biol. Cell 13: 3870-3877.

Meima ME, Weening KE, Schaap P 2003. Characterization of a cAMP-stimulated cAMP phosphodiesterase in *Dictyostelium discoideum*. J Biol Chem 278: 14356-14362.

Messner S, et al. 2009. *Physarum* nitric oxide synthases: Genomic structures and enzymology of recombinant proteins. Biochem. J. 418: 691-700.

Milne I, et al. 2009. TOPALi v2: a rich graphical interface for evolutionary analyses of multiple alignments on HPC clusters and multi-core desktops. Bioinformatics 25: 126-127.

Mooney JL, Yager LN 1990. Light is required for conidiation in *Aspergillus nidulans*. Genes & Development 4: 1473-1482.

Moriyama Y, Kawano S 2003. Rapid, selective digestion of mitochondrial DNA in accordance with the matA hierarchy of multiallelic mating types in the mitochondrial inheritance of *Physarum polycephalum*. Genetics 164: 963-975.

Morrison SD, Roberts SA, Zegeer AM, Montfort WR, Bandarian V 2008. A new use for a familiar fold: the X-ray crystal structure of GTP-bound GTP cyclohydrolase III from *Methanocaldococcus jannaschii* reveals a two metal ion catalytic mechanism. Biochemistry 47: 230-242.

Nakagaki T, Umemura S, Kakiuchi Y, Ueda T 1996. Action spectrum for sporulation and photoavoidance in the plasmodium of *Physarum polycephalum*, as modified differentially by temperature and starvation. Photochem. Photobiol. 64: 859-862.

Nei M, Kumar S. 2000. Molecular Evolution and Phylogenetics. New York: Oxford University Press.

Novak B, Tyson JJ 1993. Modeling the cell division cycle: M-phase trigger, oscillations, and size control. Journal of theoretical biology 165: 101-134.

Paddy MR, Saumweber H, Agard DA, Sedat JW 1996. Time-resolved, in vivo studies of mitotic spindle formation and nuclear lamina breakdown in *Drosophila* early embryos. Journal of Cell Science 109: 591-607.

Ponting CP, Aravind L 1997. PAS: a multifunctional domain family comes to light. Current Biology 7: R674-R677.

Rakovich T, et al. 2011. Queuosine deficiency in eukaryotes compromises tyrosine production through increased tetrahydrobiopterin oxidation. J. Biol. Chem. 286: 19354-19363.

Ronquist F, Huelsenbeck JP 2003. MrBayes 3: Bayesian phylogenetic inference under mixed models. Bioinformatics 19: 1572-1574.

Roure B, Rodriguez-Ezpeleta N, Philippe H 2007. SCaFoS: a tool for selection, concatenation and fusion of sequences for phylogenomics. BMC Evol Biol 7 Suppl 1: S2.

Ruggeri ZM, Ware J 1993. von Willebrand factor. FASEB J 7: 308-316.

Rusch HP, Sachsenmaier W, Behrens K, Gruter V 1966. Synchronization of Mitosis by the Fusion of the Plasmodia of *Physarum polycephalum*. The Journal of cell biology 31: 204-209.

Saitou N, Nei M 1987. The neighbor-joining method: A new method for reconstructing phylogenetic trees. Mol. Biol. Evol. 4: 406 - 425.

Saran S, et al. 2002. cAMP signaling in *Dictyostelium* - Complexity of cAMP synthesis, degradation and detection. Journal of Muscle Research and Cell Motility 23: 793-802.

Schaap P 2011a. Evolution of developmental cyclic adenosine monophosphate signaling in the Dictyostelia from an amoebozoan stress response. Dev Growth Differ 53: 452-462.

Schaap P 2011b. Evolutionary crossroads in developmental biology: *Dictyostelium discoideum*. Development 138: 387-396.

Schedl T, Owens J, Dove WF, Burland TG 1984. Genetics of the tubulin gene families of *Physarum*. Genetics 108: 143-164.

Schilde C, Schaap P 2013. The Amoebozoa. Methods Mol Biol 983: 1-15.

Schreckenbach T. 1984. Phototaxis and photomorphogenesis in *Physarum polycephalum* plasmodia. In: Senger H, editor. Blue light effects in biological systems. Berlin: Springer. p. 463-475.

Schultz J, Milpetz F, Bork P, Ponting CP 1998. SMART, a simple modular architecture research tool: identification of signaling domains. Proc. Natl. Acad. Sci. USA 95: 5857-5864.

Schultz JE 2009. Structural and biochemical aspects of tandem GAF domains. Handb Exp Pharmacol: 93-109.

Schulz MH, Zerbino DR, Vingron M, Birney E 2012. Oases: robust de novo RNA-seq assembly across the dynamic range of expression levels. Bioinformatics (Oxford, England) 28: 1086-1092.

Schwarz G, Mendel RR, Ribbe MW 2009. Molybdenum cofactors, enzymes and pathways. Nature 460: 839-847.

Shaulsky G, Fuller D, Loomis WF 1998. A cAMP-phosphodiesterase controls PKA-dependent differentiation. Development 125: 691-699.

Sievers F, Higgins DG 2014. Clustal omega, accurate alignment of very large numbers of sequences. Methods in molecular biology 1079: 105-116.

Singleton CK, Zinda MJ, Mykytka B, Yang P 1998. The histidine kinase dhkC regulates the choice between migrating slugs and terminal differentiation in *Dictyostelium discoideum*. Dev. Biol. 203: 345-357.

Slany RK, Kersten H 1994. Genes, enzymes and coenzymes of queuosine biosynthesis in prokaryotes. Biochemie 76: 1178-1182.

Sneath P, Sokal R. 1973. Numerical Taxonomy. San Francisco: Freeman.

Solnica-Krezel L, Burland TG, Dove WF 1991. Variable pathways for developmental changes of mitosis and cytokinesis in *Physarum polycephalum*. The Journal of cell biology 113: 591-604.

Stallmeyer B, Drugeon G, Reiss J, Haenni AL, Mendel RR 1999. Human molybdopterin synthase gene: identification of a bicistronic transcript with overlapping reading frames. Am. J. Hum. Genet. 64: 698-705.

Starostzik C, Marwan W 1995a. Functional mapping of the branched signal transduction pathway that controls sporulation in *Physarum polycephalum*. Photochem. Photobiol. 62: 930-933.

Starostzik C, Marwan W 1998. Kinetic analysis of a signal transduction pathway by time-resolved somatic complementation of mutants. J. Exp. Biol. 201: 1991-1999.

Starostzik C, Marwan W 1995b. A photoreceptor with characteristics of phytochrome triggers sporulation in the true slime mould *Physarum polycephalum*. FEBS Lett 370: 146-148.

Stover PJ 2011. Polymorphisms in 1-carbon metabolism, epigenetics and folate-related pathologies. J. Nutrigenet. Nutrigenomics 4: 293-305.

Sujatha A, Balaji S, Devi R, Marwan W 2005. Isolation of *Physarum polycephalum* plasmodial mutants altered in sporulation by chemical mutagenesis of flagellates. Eur. J. Protistol. 41: 19-27.

Takano H, et al. 2001. The complete DNA sequence of the mitochondrial genome of *Physarum polycephalum*. Mol. Gen. Genet. 264: 539-545.

Talavera G, Castresana J 2007. Improvement of phylogenies after removing divergent and ambiguously aligned blocks from protein sequence alignments. Syst Biol 56: 564-577.

Tamura K, Dudley J, Nei M, Kumar S 2007. Molecular Evolutionary Genetics Analysis (MEGA) software version 4.0. Mol. Biol. Evol. 24: 1596 - 1599.

Tamura K, et al. 2011. MEGA5: molecular evolutionary genetics analysis using maximum likelihood, evolutionary distance, and maximum parsimony methods. Mol Biol Evol 28: 2731-2739.

Taylor BL, Zhulin IB 1999. PAS domains: internal sensors of oxygen, redox potential, and light. Microbiol Mol Biol Rev 63: 479-506.

Thomason PA, et al. 1998. An intersection of the cAMP/PKA and two-component signal transduction systems in *Dictyostelium*. EMBO J. 17: 2838-2845.

Thompson J, Gibson T, Plewniak F, Jeanmougin F, Higgins D 1997. The ClustalX windows interface: flexible strategies for multiple sequence alignment aided by quality analysis tools. Nuc. Acids Res. 24: 4876 - 4882.

Veltman DM, Keizer-Gunnik I, Van Haastert PJ 2008. Four key signaling pathways mediating chemotaxis in *Dictyostelium discoideum*. J Cell Biol 180: 747-753.

Wallace IM, O'Sullivan O, Higgins DG, Notredame C 2006. M-Coffee: combining multiple sequence alignment methods with T-Coffee. Nucleic Acids Research 34: 1692-1699.

Wang N, Shaulsky G, Escalante R, Loomis WF 1996. A two-component histidine kinase gene that functions in *Dictyostelium* development. EMBO J. 15: 3890-3898.

Watschinger K, et al. 2010. Identification of the gene encoding alkylglycerol monooxygenase defines a third class of tetrahydrobiopterin-dependent enzymes. Proc. Natl. Acad. Sci. U.S.A. 107: 13672-13677.

Werner ER, Blau N, Thony B 2011. Tetrahydrobiopterin: biochemistry and pathophysiology. Biochem. J. 438: 397-414.

Wheals AE 1970. A homothallic strain of the myxomycete *Physarum polycephalum*. Genetics 66: 623-633.

Williams JG 2006. Transcriptional regulation of *Dictyostelium* pattern formation. EMBO Rep 7: 694-698.

Wolanin P, Thomason P, Stock J 2002. Histidine protein kinases: key signal transducers outside the animal kingdom. Genome Biology 3: reviews3013.3011 - reviews3013.3018.

Zdobnov EM, Apweiler R 2001. InterProScan--an integration platform for the signature-recognition methods in InterPro. Bioinformatics 17: 847 - 848.

Zerbino DR, Birney E 2008. Velvet: algorithms for de novo short read assembly using de Bruijn graphs. Genome Research 18: 821-829.

Zinda MJ, Singleton CK 1998. The hybrid histidine kinase dhkB regulates spore germination in *Dictyostelium discoideum*. Dev. Biol. 196: 171-183.

Zuckerkandl E, Pauling L. 1965. Evolutionary divergence and convergence in proteins. In: Bryson V, Vogel HJ, editor. Evolving Genes and Proteins. New York: Academic Press.
